# Conserved structure and inferred evolutionary history of long terminal repeats (LTRs)

**DOI:** 10.1186/1759-8753-4-5

**Published:** 2013-02-01

**Authors:** Farid Benachenhou, Göran O Sperber, Erik Bongcam-Rudloff, Göran Andersson, Jef D Boeke, Jonas Blomberg

**Affiliations:** 1 Section of Virology, Department of Medical Sciences, Uppsala University, Uppsala, Sweden; 2 Unit of Physiology, Department of Neuroscience, Uppsala University, Uppsala, Sweden; 3 Department of Animal Breeding and Genetics, Swedish University of Agricultural Sciences, Uppsala, Sweden; 4 High Throughput Biology Center, Johns Hopkins University School of Medicine, Baltimore, MD 21205, USA; 5 Section of Virology, Department of Medical Sciences, Academic Hospital, Uppsala 751 85, Sweden

**Keywords:** LTR, Long terminal repeat, Retrotransposon, Retrovirus, Phylogeny, Genome evolution

## Abstract

**Background:**

Long terminal repeats (LTRs, consisting of U3-R-U5 portions) are important elements of retroviruses and related retrotransposons. They are difficult to analyse due to their variability.

The aim was to obtain a more comprehensive view of structure, diversity and phylogeny of LTRs than hitherto possible.

**Results:**

Hidden Markov models (HMM) were created for 11 clades of LTRs belonging to *Retroviridae* (class III retroviruses), animal *Metaviridae* (Gypsy/Ty3) elements and plant *Pseudoviridae* (Copia/Ty1) elements, complementing our work with *Orthoretrovirus* HMMs. The great variation in LTR length of plant *Metaviridae* and the few divergent animal *Pseudoviridae* prevented building HMMs from both of these groups.

Animal *Metaviridae* LTRs had the same conserved motifs as retroviral LTRs, confirming that the two groups are closely related. The conserved motifs were the short inverted repeats (SIRs), integrase recognition signals (5´TGTTRNR…YNYAACA 3´); the polyadenylation signal or AATAAA motif; a GT-rich stretch downstream of the polyadenylation signal; and a less conserved AT-rich stretch corresponding to the core promoter element, the TATA box. Plant *Pseudoviridae* LTRs differed slightly in having a conserved TATA-box, TATATA, but no conserved polyadenylation signal, plus a much shorter R region.

The sensitivity of the HMMs for detection in genomic sequences was around 50% for most models, at a relatively high specificity, suitable for genome screening.

The HMMs yielded consensus sequences, which were aligned by creating an HMM model (a ‘Superviterbi’ alignment). This yielded a phylogenetic tree that was compared with a Pol-based tree. Both LTR and Pol trees supported monophyly of retroviruses. In both, *Pseudoviridae* was ancestral to all other LTR retrotransposons. However, the LTR trees showed the chromovirus portion of *Metaviridae* clustering together with *Pseudoviridae*, dividing *Metaviridae* into two portions with distinct phylogeny.

**Conclusion:**

The HMMs clearly demonstrated a unitary conserved structure of LTRs, supporting that they arose once during evolution. We attempted to follow the evolution of LTRs by tracing their functional foundations, that is, acquisition of RNAse H, a combined promoter/ polyadenylation site, integrase, hairpin priming and the primer binding site (PBS). Available information did not support a simple evolutionary chain of events.

## Background

Retroviruses are positive strand RNA-viruses which infect vertebrates [1,2]. After reverse transcription to a DNA form (a provirus) they can integrate in a host cell chromosome. If this cell belongs to the germ line integrated proviruses can thereafter be inherited in a Mendelian fashion and thereby become endogenous retroviruses (ERVs). Retroviruses contain at least four protein-coding genes: the *gag*, *pro*, *pol* and *env* genes. These genes are flanked by two identical direct repeats, the long terminal repeats (LTRs) that contain regulatory elements for proviral integration and transcription as well as retroviral mRNA processing. Retroviruses are here divided into three main groups: class I including Gammaretroviruses and Epsilonretroviruses, class II including Betaretroviruses and Lentiviruses and class III including Spumaretroviruses [3,4]. This classification, originally based on human endogenous retrovirus (HERV) studies [5], can be extended to include all retroviruses (ERVs and exogenous retroviruses (XRVs)). As more genomes are sequenced, it becomes obvious that much of retroviral diversity is not yet covered by existing classifications. However, in the classification of the International Committee on the Taxonomy of Viruses (ICTV) [6] the retroviruses belong to the family *Retroviridae* with class I and II in the subfamily *Orthoretrovirinae* and class III mainly in *Spumaretrovirinae*. Here, we use the ICTV nomenclature together with the older retrotransposon nomenclature.

The genomes of non-vertebrate eukaryotic phyla also harbour retrovirus-like LTR-containing elements called LTR retrotransposons [7]. They fall into three distinct groups: the *Pseudoviridae* (Copia/Ty1) group, present in plants, fungi and metazoans [8,9], the *Metaviridae* (Gypsy/Ty3), found also in plants, fungi and metazoans ([10,11] and the *Semotivirus* (Bel/Pao) group found exclusively in metazoans [12]. The most diverse group is *Metaviridae*, which consists of around 10 subgroups [12]. One of them, the chromoviruses, has a wider host range, being found in plants, fungi and vertebrates. Chromoviruses got their name because their *pol* gene encodes an integrase with a chromodomain (‘chromatin organization modifier domain’), a nucleosome-binding integrase portion which can mediate sequence specific integration ([10,13-15]. Ty3 of yeast is part of the chromovirus clade even though some members of this clade, including Ty3, do not have a chromodomain in their integrase [13]. *Pseudoviridae* can be divided into at least six main groups [12]. According to the ICTV classification, *Metaviridae* contains three genera; the *Semotivirus* corresponding to Bel/Pao, the *Metavirus* (represented by Ty3) and *Errantivirus* (Gypsy). *Pseudoviridae*, is also divided into three genera; the *Sireviru*s, *Hemivirus* (Copia) and *Pseudovirus* (Ty1). The ICTV classification is in need of revision to account for the diversity of LTR retrotransposons [12]. The LTR retrotransposons are important elements of plant genomes. In both maize (*Zea mays*) and broad bean (*Vicia faba*), for example, LTR retrotransposons account for more than 50% of the respective genomes [8].

The relationships of LTR retrotransposons have primarily been studied by constructing phylogenetic trees based on the reverse transcriptase (RT)-domain of Pol, the most conserved retroelement domain [16,17]. According to the RT phylogeny, *Pseudoviridae* is the ancestral group, and *Metaviridae* and vertebrate retroviruses are sister groups. *Semotivirus*, *Metaviridae* and retroviruses may have arisen from the same ancestor because most of them share the same domain arrangement in Pol, with the integrase (IN) domain coming after RT and RNAse H. In Copia/Ty1 and the rGmr1 member of *Metaviridae*, IN comes before RT and RNAse H [7]. In spite of *Pseudoviridae* being ancestral it has apparently diversified less than *Metaviridae*. In recent years, however, more *Pseudoviridae* have been discovered in basal organisms such as diatoms [18].

In addition, phylogenies of the RNAse H and IN domains of Pol were previously reported [13]. No major disagreement was found among them, indicating that these domains were not exchanged between groups, even though the retroviral RNAse H seems to have been independently acquired [19].

The evolutionary relationships among different subgroups of *Metaviridae* remain to be resolved. Even for retroviruses, the relative tree positions of class I and class III retroviruses is uncertain but they seem to have branched off earlier during evolution than class II retroviruses. This is consistent with the wider distribution of gamma- and epsilonretroviruses which are highly represented in fish [20]. Epsilon- and gammaretroviruses share several taxonomic traits, and are on the same major branch in a general retroviral tree [4].

The common structure of retroviral LTRs was recently investigated using Hidden Markov Models (HMMs) [21]. LTRs can be divided into two unique portions (U3 and U5), and a repeated (R) region in between them. R and U5 are generally more conserved than U3. The higher variability of U3 may be due to adaptation to varying tissue environments. In the HMMs, the conservation was highest for the Short Inverted Repeat (SIR) motifs TG… and …CA at both ends of the LTR, plus one to three AT-rich regions providing the LTRs with one or two TATA-boxes and a polyadenylation signal (AATAAA motif). The precise delineation of U3/R/U5 borders depends on sequencing of retrotransposon RNA, critical information that is often missing. Moreover, none, one or several TATA boxes may exist. Initiator (INR) motifs (TCAKTY) may or may not be present. Alternative transcriptional start sites (TSSes) and antisense transcription are also common [21]. Thus, LTR structure and function are complex and often cannot be encapsulated by simple schemes.

Three groups of retroviral LTRs were earlier modeled by means of HMMs in [21,22]; alignments and phylogenetic trees were generated for the human betaretroviral mouse mammary tumor virus (MMTV)-like (HML), the lentiviral and the gammaretroviral genera. The aim of this study was to extend the analysis to groups of LTRs belonging to *Pseudoviridae* and *Metaviridae* making it possible to uncover the putative conserved structure of all major groups of LTRs and to study their phylogeny.

## Results

### HMMs, regularisation and phylogeny

In Benachenhou *et al*. [21] and Blikstad *et al*. [22], HMMs were used to align and construct phylogenies of LTRs for the HML, the lentiviral and the gammaretroviral genera. The LTR phylogenies were largely congruent with the phylogenies of their RT domains. The HMMs were created by using a set of sequences, which was a representative sample of the family of interest, the so-called training set. A well-known problem in HMM-modelling is that the HMMs become too specialised to the training set. To alleviate this problem one has to regularise the HMMs, which amounts to adding or removing random noise from the data. It turned out that removing random noise produced worse HMMs. It is a common experience in pattern recognition algorithms that adding noise to the training set may diminish the tendency to over-learning and the tendency to lock on to local maxima.

A test set containing sequences not present in the training set was then used to evaluate the regularised HMMs. The method was subsequently improved to systematically search for the best phylogenetic tree, that is, the one with the highest mean bootstrap value [23].

### Model building

The HMMs for the *Metaviridae* LTRs were obtained as follows: first, the internal coding sequences were clustered into 14 clusters (Additional file 1: Table S1). For each cluster the corresponding LTRs were then selected. Each LTR cluster was randomly divided into a training set comprising 80% of the sequences and a test set with the remaining sequences. The training set was used to calculate the many parameters of the HMM. The HMM enables one to assign a probability or score for any given sequence. Sequences from the training set will usually get a high score. That is why the average score of the test set was calculated in order to evaluate the HMM. If it was high enough (Table 1) then the HMM was considered a ‘good’ model of the LTR group. Many clusters were too divergent to directly yield such ‘good’ HMMs but it was nevertheless possible to construct six HMMs for the *Metaviridae* LTRs (see Table 1). They modelled the following six clades: Zam, belonging to the Errantiviruses (found in insects), Mag C (in metazoans, including vertebrates), part of Mag A (in the mosquito *Anopheles gambiae*), CsRN1 (in metazoans excluding vertebrates), Sushi, which are chromoviruses related to the Metavirus Ty3 (in fungi and fish) and, finally, rGmr1 (in fish). The Zam clade was one of three distinct subgroups in the Errantivirus cluster based on Pol amino acids. Mag C (containing SURL [12]), CsRN1 and rGmr1 HMMs were based on the original clusters. The Mag A cluster (containing Mag proper [12]) did not produce a good HMM, however it was possible to build an HMM trained on the subset of Mag A LTRs from *Anopheles gambiae* (here called Mag A even if restricted to *Anopheles gambiae*). Finally, the chromovirus cluster was by far the most diverse; an HMM trained on one of its well-defined subgroups, mainly containing LTRs from *Danio rerio*, was successfully built (Sushi). The Zam, Mag C and CsRN1 training sets contained sequences from different hosts whereas the training set from Mag A, Sushi and rGmr1 were dominated by sequences from a single host (Additional file 1: Table S2).

**Table 1:** Description of models

**Name**	**Taxon**	**Host**	**Number in training set**	**Train**_**length**	**M**	**Score**_**test**
Zam	Gypsy/Ty3	Insects	8	318	150	33
Mag C	Gypsy/Ty3	Metazoans	16	174	50	21
Mag A	Gypsy/Ty3	Mosquito	20	213	90	41
CsRN1	Gypsy/Ty3	Metazoans	13	330	90	27
Sushi	Gypsy/Ty3	Fungi/Metazoans	24	572	150	46
rGmr1	Gypsy/Ty3	Fish	34	695	150	42
Sire	Copia/Ty1	Plants	68	373	150	49
Retrofit	Copia/Ty1	Plants	32	220	150	74
HML	Retroviruses	Primates	23	390	150	81^a^
Gamma	Retroviruses	Vertebrates	72	521	150	50^a^
Class III endogenous retroviruses	Retroviruses	Vertebrates	70	504	150	51

Name, taxonomic group, host, number of training sequences, average length of training sequences, chosen number of match states *M* and score of test set are given for each HMM model. The constituents of the training sets are shown in Additional file 1.

^a^No test set; score of training set is shown.

These clades cover some of the diversity of animal *Metaviridae*. The alignments generated by the corresponding models were also visually inspected. The six models all had conserved SIRs (TG…CA), except for most LTRs in the Zam clade (which had 5^′^5'AGTTA .. 3^′^TAATT or .. the imperfect inverted repeat 3^′^TAACT) and an AATAAA motif.

In the same way, the internal coding sequences from *Pseudoviridae* fell into two main groups which could be subdivided into five clusters in total (Additional file 1: Table S1). Two clusters generated convergent HMMs: Sire (a *Sirevirus*) and Retrofit (a *Pseudovirus*), both in plants [8]. Most of the Sire cluster was used for the Sire HMM whereas a subgroup comprising half of the sequences in the Retrofit cluster was used for the corresponding HMM. Both training sets contained many sequences from *Sorghum bicolor* (about 60%). The better known Copia *sensu stricto*, which is a *Hemivirus* of insects and Ty1, a *Pseudovirus* in yeast, did not yield convergent models because the sequence sets were highly diverse and/or contained too few LTRs. The two plant LTR models both displayed SIRs and a TATATA motif.

Finally, two retroviral LTR models (HML and gammaretroviruses) were taken from [21,22] to which a class III retroviral model was added (Table 1). In comparison to *Metaviridae* it was relatively easy to build HMMs for those retroviral LTRs. Like for *Metaviridae*, the retroviral LTRs had an AATAAA motif in addition to SIRs.

### Detection

To further evaluate the models, genomic DNA sequences of *Drosophila melanogaster*, *Anopheles gambiae*, *Danio rerio*, and *Oryza sativa* was screened for occurrence of LTRs and compared to the RepeatMasker output for the chromosome. The number of LTRs detected and the number of LTRs missed are shown in Table 2 for each *Metaviridae* and Pseudoviridae clade (detection of retroviral LTRs was investigated in [22]). Two sets of LTRs were searched for: all LTRs in the clade and only the LTRs not already belonging to the training set. This distinction was done because LTRs from the training set are expected to be detected more easily due to overfitting. The sensitivities ranged from 8% to 75% except for the Mag C model which had 0% sensitivity, probably because its HMM had too few match states (50). The threshold was chosen in such a way that the sensitivity was as high as possible, still limiting the number of additional positives to at most 100. Additional positives are those LTR candidates detected by the HMM but not by RepeatMasker. Most were random non-LTR elements but in some cases a few percent were other more or less related LTRs. LTR fragments reported by RepeatMasker were discarded unless they were at least 100 bp long and ending at most 100 bp from the 3^′^ end of the LTR consensus; the latter requirement was imposed because the 3^′^ end is where most of the conservation resides (see [21] and below). HMMs with more match states were preferred if they yielded significantly higher sensitivities.

**Table 2:** Detection performance of HMMs

**Name**	**M**	**Organism**	**Chromosome**	**Threshold**	**Detected ( *n *)**	**Missed ( *n *)**	**Additional positive ( *n *)**	**Sensitivity (%)**
Zam^a^	290	*Drosophila melanogaster*	3L	23	6 (13)	4 (1)	80 (84)	60 (93)
		*Anopheles gambiae*	2R					
Mag C^b^	50	*Anopheles gambiae*	2R	14	0 (0)	0 (12)	155	NA (0)
Mag A	90	*Anopheles gambiae*	2R	20	3 (25)	1 (34)	36	75 (42)
CsRN1	90	*Anopheles gambiae*	2R	12	1 (31)	0 (11)	117	100 (74)
Sushi	270	*Danio rerio*	7	35	8 (46)	24 (78)	122	25 (37)
rGmr1	150	*Danio rerio*	7	20	7 (35)	78 (260)	38	8 (12)
Sire	150	*Oryza sativa*	1	20	3 (10)	1 (3)	53	75 (77)
Retrofit	150	*Oryza sativa*	1	35	4 (8)	2 (2)	4	67 (80)

The detection performance was not extensively evaluated. The number of LTRs detected in one chromosome of a suitable eukaryotic organism, the number of LTRs missed and the number of additional positives as compared to the RepeatMasker output for the chosen chromosome are shown. The threshold, number of match states *M* and sensitivity are also tabulated. Two numbers for the sensitivity are reported, first the sensitivity for LTRs not belonging to the training set and second the sensitivity for all LTRs in the clade of interest between parentheses.

^a^Two chromosomes from different organisms were screened.

^b^All LTRs in the clade belonged to the training set.

Previous studies [21,23] have shown that the HMMs can be used to detect solo LTRs and even detect new groups if they are not too distantly related; for example an HMM trained on HML2-10 can detect 52% of HML1. However, the more general the HMM the less sensitive and specific it becomes. For efficient detection one needs sufficiently specialised HMMs which also implies more of them. The focus of this paper was however to show that it is possible to build HMMs for *Metaviridae* and *Pseudoviridae* LTRs. The detection aspect was considered mainly as a way of validating the HMMs. In particular many *Metaviridae* HMMs in Table 2 had quite poor detection capabilities.

### Conserved LTR structure

A major challenge in determining the evolutionary trajectory of LTRs relates to the definition of the three segments U3, R and U5. This is a trivial matter for those elements for which the 5^′^ terminus and site(s) of polyadenylation of the RNA have been experimentally determined. Regrettably, although such data are available for most retroviruses for which RNA can readily be extracted in pure form from virions, equivalent data do not exist for the majority of retrotransposons. While it may be possible in some cases to extract such information from high throughput RNASeq datasets, preliminary studies indicate that the precision of mapping by this method ranges from moderately high (the highly expressed Ty1 in *Saccharomyces cerevisiae*) to non-existing (very poorly expressed Ty4 in *S*. *cerevisiae*) (Yizhi Cai and JD Boeke, unpublished data). Therefore, the ability to accurately predict such boundaries from primary sequence data combined with sophisticated alignment algorithms is potentially very valuable in understanding LTR structure and as an adjunct to RNASeq analyses.

**Figure 1:**
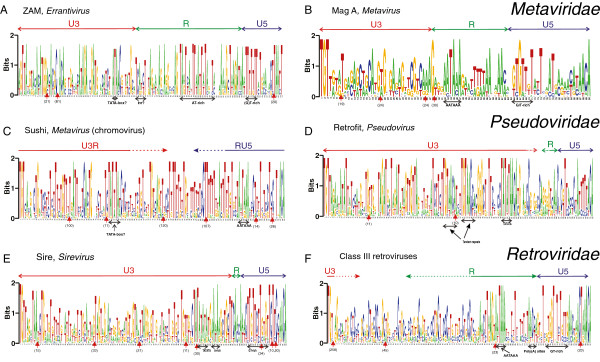
**Weblogos of *Metaviridae, Pseudoviridae *and *Retroviridae *LTRs. **(**A**) Weblogo for a Viterbi alignment of the Zam training set. Major insertions are indicated as red triangles with the number of inserts below them. The heights of the letters are a measure of how well conserved the residues are. Two bits correspond to 100% conservation. (**B**) Weblogo for a Viterbi alignment of the Mag A training set. (**C**)Weblogo for a Viterbi alignment of the Sushi training set. (**D**) Weblogo for a Viterbi alignment of the Retrofit training set. (**E**) Weblogo for a Viterbi alignment of the Sire training set. (**F**)Weblogo for a Viterbi alignment of the training set of class III retroviruses.

Weblogos corresponding to HMM-generated alignments and the inferred U3/R and R/U5 boundaries are shown for Zam, Mag A, Sushi, Sire, Retrofit and class III retroviruses in Figure 1A-F. Precise location of the U3/R and R/U5 boundaries requires RNA sequencing. As stated above, such data are not available for most of the LTRs.

#### General remarks on the HMMs

The conserved elements common to most groups are the TATA box and in some clades TGTAA upstream of the TATA box, the AATAAA motif, the GT-rich area downstream of the polyadenylation site, and the SIRs at both ends of the LTR. The TATA motif is more conserved for the plant retrotransposons than for the metazoan retrotransposons whereas the opposite is true for the AATAAA motif. Although ‘TG’ and ‘CA’ are the most conserved portions of the SIRs, the conservation of the SIRs extends approximately seven bp into the LTR. The SIRs are somewhat longer in *Pseudoviridae*. The general consensus is TGTTRNR at the 5^′^end and YNYAACA at the 3^′^ end, in perfect complementarity. The SIRs bind to the integrase enzyme; therefore their conservation is presumed to reflect the specificity of the bound protein. From previous studies it is known that the integrase binding specificity resides in the terminal eight to fifteen bp [24], in agreement with the HMM models. The reason for the variation in SIR length is unknown.

The U3 region in the weblogos is proportionally smaller than the true length of U3; this is because its sequence is much less well conserved with few recognizable motifs (excepting the TATA box). The latter is also true for the R region whenever it is long such as in gammaretroviruses, class III endogenous retroviruses/spumaviruses and lentiviruses. This ‘residual’ conservation in the longer R-regions can be linked to stem-loop structures [21]. Stem-loop structures favour conservation in both complementary parts of the stem. The HMMs have proven to be apt for finding conservation in LTRs despite their immense variability in length and conserved elements. As explained in Benachenhou *et al*. [21], the X axes in the HMMs are ‘match states’, a conserved subset of the nucleotides in the training LTRs. Less conserved nucleotides (‘insert states’) are not shown in the HMM, but are displayed in a Viterbi alignment of LTRs analysed with the HMMs. Depending on the training parameters, the HMM length is somewhat arbitrary but the conserved motifs in the shorter HMMs are always found in the longer ones. Beyond a certain length, the HMMs merely expand the length of the quasi-random regions in the LTR and thus provide limited additional information. If the HMMs are too short, some conserved motifs can be missed as was observed for class III retroviruses. In contrast, longer HMMs may display all conserved motifs but at the expense of unnecessarily long stretches of quasi-randomness, that is, variable nucleotides artificially elevated to the status of ‘match states’. This is an especially severe problem when modelling long LTRs (>1,000 bp). The subject of building LTR HMMs is further described in Benachenhou *et al*. [21]. The match and insert states are shown for six HMMs in Additional file 2.

#### Zam

The approximate locations of U3, R and U5 of these *Errantivirus* elements, belonging to *Metaviridae*, in Figure 1A were determined using experimental results for the TED element [25] which is part of the training set. The AATAAA signal is not very clear but a relatively long AT-rich stretch is apparent in R (pos. 92–111).

The U5 region begins with a GT-rich stretch, a probable polyadenylation downstream element. Another conserved AT-rich stretch is found immediately upstream of the Transcriptional Start Site (TSS) and is therefore probably an analogue of a TATA box. The TSS may possibly be part of an INR at pos. 67–72. Its short sequence (TCAT(C or T)T) closely resembles the INR consensus of *Drosophila* (TCA(G or T)T(T or C)) [26]. The INR element is a core promoter element overlapping the TSS and commonly found in LTRs, which can initiate transcription in the absence of a TATA box [26-28].

The SIRs are shown in Table 3. The LTRs of the Zam group thus have the same overall structure as retroviral LTRs and are similar to gammaretroviral LTRs [21], a fact noted long ago [29]. However, the Zam SIRs lack the consensus TG..CA of other LTRs.

**Table 3:** Integrase recognition motifs

**Name**	**5**^′^**INT motif**	**3**^′^**INT motif**
Zam	AGTTAYRK	TAAYT
Mag C	TGTNATRT	AYANAACA
Mag A	TGTTRKR	RMRWMYAYAACA
CsRN1	TGTGGYG	MSYYAMA
Sushi	TGTYANR	CNTNACA
rGmr1	TGTNANR	CGTNACA
Sire	TGTTRRN(3)TAA	TT(20)CYAACA
Retrofit	TGTTARAMNAT(1)AT	ATT(1)TTYA(1)CA
HML	TGTNGGGRAARG	CCCNRCA
Gamma	TGWAGNMRR	NNYNACA
Class III endogenous retroviruses	TGT	ACA

Integrase recognition motifs (also called *att* sites) at the 5^′^ and 3^′^ ends of LTRs are shown in Table 3. The IUPAC code for nucleic acids is used. The number of inserts is shown between parentheses.

Compared to the other weblogos below, Zam has a less clear AATAAA motif but is otherwise similar to the other weblogos.

#### Mag A

This *Metaviridae* clade (belonging to genus *Metavirus*) has a clear AATAAA signal (Figure 1B) but no conserved TATA-box. Because of lack of experimental evidence, the division into U3, R and U5 cannot be clearly defined for this clade. The beginning of U5 was chosen to coincide with a G/T-rich stretch, a probable polyadenylation downstream element [21]. The border between U3 and R cannot be located with precision but it should be upstream of the AATAAA signal.

#### Sushi

The weblogo of this chromoviral clade (Figure 1C) has a clear AATAAA motif and a conserved AT-rich stretch at pos. 51–57 which could serve as a TATA-containing promoter. Two differences from other retroviruses and most *Metaviridae* LTR retrotransposons are noticeable. Firstly, the AATAAA motif is significantly closer to the 3^′^ end of the LTR and secondly, U3 is more T-rich. This last feature is shared by the non-chromoviral rGmr1 LTRs (not shown).

#### Retrofit and Sire

LTRs of Retrofit and Sire, two of the main groups (*Pseudovirus* and *Sirevirus*, respectively) of *Pseudoviridae*, have similar structures and are clearly different from retroviral and *Metaviridae* LTRs. Retrofit and Sire are shown in Figure 1D and E. The most striking feature is a highly conserved TATATA motif. This motif has previously been found in Bare-1 [30], Tnt1 [31], both related to Sire; and another clade of Sireviruses [32], phylogenetically distinct from the ones used in the present study. The TATATA motif is known to function as a TATA box [30].

The CAACAAA motif at pos. 120–126 in Sire (Figure 1E) is shared by Tnt1 where it serves as a polyadenylation site [33,34]. Retrofit has a similar CAA motif at pos. 127–129 (Figure 1D). In Sire, the polyadenylation site is surrounded by T-rich stretches as is typical of plant genomes [34].

Retrofit (Figure 1D) and Tnt1 [33] completely lack an AATAAA motif, suggesting that the TATATA motif has a dual role both as promoter and poly(A) signal as has been established previously for the particular case of HML retroviruses (but not for other retroviruses) [21]. Plant genomes generally have fewer constraints on the polyadenylation signal than animal genomes [34]; any A-rich motif may do. The same applies to yeast genomes [35]. Sire has however an additional A-rich motif immediately following the TATATA motif (Figure 1E). The endpoints of the R region in Sire in Figure 1E were estimated by comparing it with the related tnt1 [31,36] whereas the beginning of R in Retrofit could not be located. It is however clear that R in both Sire and Retrofit is very short (for Sire 10 bp long) because of the proximity of the TATA box to the polyadenylation signal. This is in contrast to retroviruses where the size of R varies a lot: MMTV (mouse mammary tumour virus) 11 bp [37]; RSV (Rous sarcoma virus) 21 bp [37]; ERV gammaretroviruses 70 bp and lentiviruses 150 bp (calculated from the average length of the corresponding training sets in Benachenhou *et al*. [21]).

Retrofit has two well-conserved TGTAAC(C)A sequences upstream of the TATATA (Figure 1D). Tandem repeats of various sizes are often found in the U3 region of retroviruses [38,39], where they can play a role in transcription regulation. Such tandem repeats were discovered almost 20 years ago in tobacco Tnt1 [31]. A TGTAA motif is also found in a weblogo of Sire with more match states (see discussion of longer HMMs below under Class III retroviruses, and Additional file 2: Figure S1) and in gammaretroviruses (Additional file 2: Figure S2), it also lies upstream of the TATA box.

Most of the U3 region in Retrofit and Sire consists of a seemingly random region depleted of Cs (Figure 1D and E). This contrasts with the frequent occurrence of conserved cytosines in U3s of class III ERVs, spumaviruses and gammaretroviruses, especially close to the U3/R border (Figure 1F, and Benachenhou *et al*. [21]). Finally, the 5^′^ integrase recognition motifs are very similar in Retrofit, Sire and also in Ty1 from yeast: TGTTARAMNAT(1)AT, TGTTRRN(3)TAA and TGTTGGAATA, respectively, where (1) and (3) are the average lengths of non-conserved insertions (cf. Table 3).

#### Class III endogenous retroviruses

As for animal *Metaviridae* and other retroviral elements the best conserved motif is the AATAAA motif (Figure 1F). Not apparent in Figure 1F but visible in HMMs with more match states (Additional file 2: Figure S3) is a less-conserved TATA box. The nucleotide composition of the 180 bp region between the probable TATA box and the AATAAA motif is depleted of As; this is also a feature of other retroviruses such as lentiviruses and gammaretroviruses (see Additional file 2: Figure S2 for gammaretroviruses). There are also strong similarities with the *Metaviridae* element Mag A downstream of the polyadenylation signal (compare Figure 1B and F).

### LTR phylogeny

**Figure 2:**
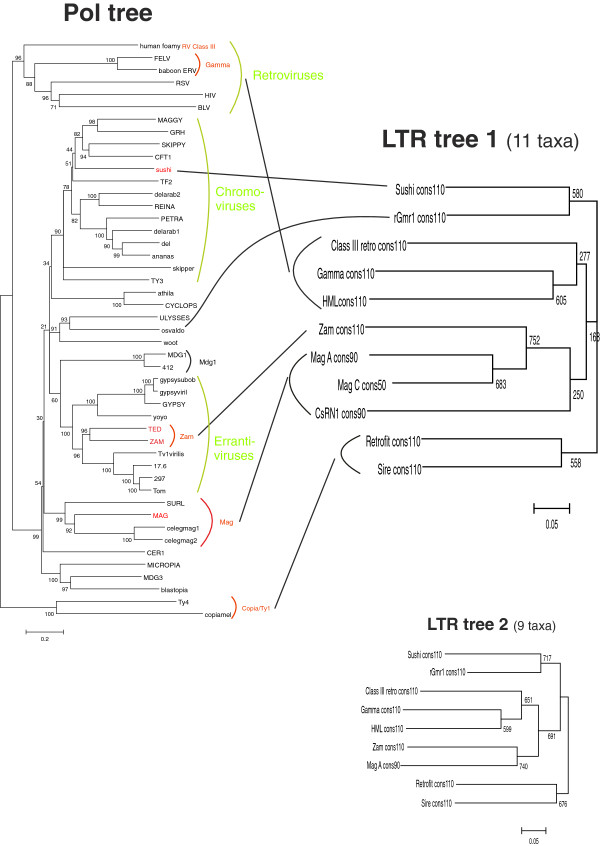
**Pol tree *versus *LTR tree.** (Left) Neighbour-joining tree based on a concatenated alignment of RT- RNAse H- and IN- sequences coming from 47 LTR retrotransposons. (Right) Two neighbour-joining trees generated from Viterbi alignments of LTR HMMs trained on sets containing HMM consensuses from Table 1. The upper tree is based on 11 consensuses whereas the lower tree is based on nine. Both are congruent, but the second has better bootstrap support. ClustalW [40] was used with 1,000 bootstrap replicates and default parameters.

To further investigate the relationships between different LTR groups, a general HMM describing all LTRs was built as follows: for each LTR group a consensus was generated by the corresponding HMM and the set of all group consensuses was used to train a general LTR HMM. The resulting ‘Superviterbi’ alignment yielded a neighbour-joining tree. The substitution model used was p-distance, that is, the proportion of nucleotide differences between a pair of sequences. This is the simplest substitution model and it was chosen because the LTR consensus alignments cannot be considered accurate except for the SIRs. The number of match states of the group consensuses was varied as was the number of match states in the general HMM and the regularisation parameter *z*[22]. The trees with higher mean bootstrap values were selected. Two LTR trees are shown in Figure 2. The first one has 11 taxa whereas the second one has nine taxa but better bootstrap support. Both trees are congruent.

The LTR tree can be compared to a neighbour-joining tree obtained from an alignment, which is a concatenation of the three Pol domains RT, RNAse H and INT (see Figure 2). The alignments are from [13] and are available at the EMBL online database (accession numbers DS36733, DS36732 and DS36734).

Four LTR groups were apparent: (1) The two *Pseudoviridae* LTRs Retrofit and Sire; (2) The retroviruses; (3) The *Metaviridae* LTRs, Zam, Mag C, Mag A and CsRN1; and (4) a more heterogeneous second group of *Metaviridae*, Sushi and rGmr1. Inspection of the Weblogos gives further support for these groups: Retrofit/Sire, and to a lesser degree Sushi and rGmr1, are different from the other LTRs with respect to conserved motifs and/or nucleotide composition. Note that the retroviruses cluster with the first *Metaviridae* group although at low support in the larger LTR tree. Most high bootstrap trees tended to give the same topology as the tree shown in Figure 2.

**Figure 3:**
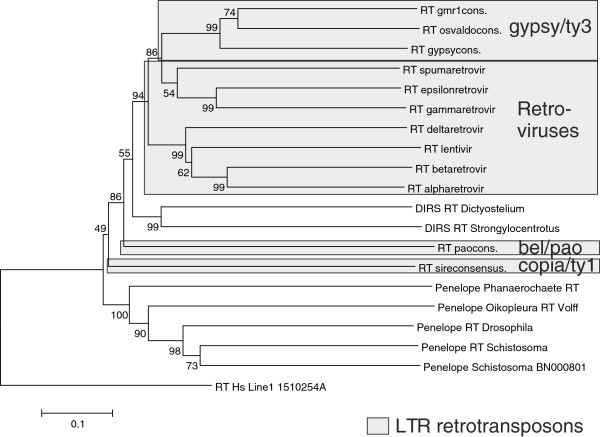
**RT**-**based inference of retroelement phylogeny.** ClustalW [40], and the maximum likelihood algorithm, as embodied in the Mega program package [41], was used with 500 bootstrap replicates and default parameters. The bootstrap percentages are shown at each bifurcation. RT consensus sequences were obtained from the Gypsy database (LTR retroelements), or from GenBank (Line1 and Penelope).

**Figure 4:**
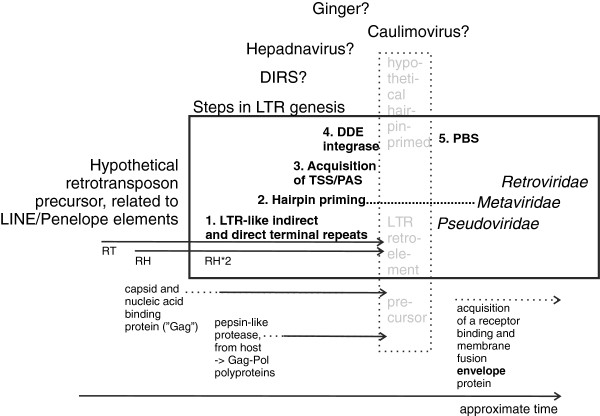
**A model for LTR retrotransposon evolution.** The figure is an attempt to reconstruct a parsimonious sequence of events leading to LTR retrotransposons. It is based on the RT trees shown in Figure 3 and Additional file 2: Figure S4. Five stages of LTR genesis are postulated: 1. Addition of LTR-like terminal repeats which 2. can hairpin prime, 3. A promoter structure next to one of these repeats, in the vicinity of a polyadenylation signal/site. 4. A DDE integrase, acquired in several independent events. 5. A PBS which replaced the hairpin primer. This led to full LTR function. The addition of capsid, protease and envelope protein genes are also marked. PAS, Polyadenylation signal and site; PBS, Primer binding site; RH, RNAse H; RT, Reverse transcriptase; RV, Retroviruses (Classes I, II and III); TSS, Transcriptional start site.

In an attempt to further trace the origins of LTRs and LTR retrotransposons, we constructed trees of reverse transcriptases from the RNA transposons LINE1, Penelope and DIRS, as well as the hepadna and caulimo DNA viruses. Although the trees had relatively low bootstrap values, the branch patterns were as in Figure 3 (cf. Additional file 2: Figure S4). Like in the polymerase-based tree of Figure 2, among LTR transposons Pseudoviridae is the most ancestral, followed by Retroviridae and Metaviridae. The positions of DIRS elements, and caulimo and hepadna viruses relative to the LTR transposons differ, illustrating the complexity of phylogenetic inference for retrotransposons and reverse transcribing viruses. We tried to reconcile this with a successive addition of features necessary for creation of LTRs, that is, RNAse H, a combined promoter and polyadenylation site (TSS/PAS), primer binding site (PBS) and an integrase, (Figure 4). The uncertain evolutionary position of the related DIRS, DNA viruses and Ginger DNA transposon is symbolised with question marks.

## Discussion

Our LTR structure analysis did not cover all LTR-retrotransposons, either because of LTR length, profound variation or scarcity of sequences in some clades. However, the commonality of structure of those from which we succeeded in building HMMs was striking. It was possible to construct models of LTRs from some groups of LTR retrotransposons and retroviruses, fathoming much of the LTR diversity. This allowed scrutiny of their phylogeny in a rather comprehensive way, and comparison with phylogenies of other retrotransposon genes. The HMMs should be useful for detection of both complete LTR retrotransposons and single LTRs. However, the focus of this study was not on detection *per se* but rather on assessing conservation. We assessed the possible conservation of structural features of LTRs of LTR retrotransposons from non-vertebrates and vertebrates (mainly retroviruses), in an effort to trace LTR evolution in a broad context of LTR retrotransposon evolution.

In a previous paper [21] we noted a common LTR structure among the orthoretroviruses. The present work shows a unity of LTR structure among a wide variety of LTR retrotransposons. LTRs are complex structures, and have a complex ontogeny. In spite of this they have a unitary structure. This indicates that the basic LTR structure was created once in a prototypic retrotransposon precursor, an argument for LTR monophyly, contrasting with the polyphyletic model of LTR retrotransposon evolution [12]. When LTRs are SuperViterbi aligned, they tend to cluster similarly to the clustering of other retroviral sequences (RT, *gag*, PRO and IN) [22]. There are however, notable exceptions, which will be discussed below.

LTR evolution must be seen in the context of evolution of host promoters. For example, the gradual development of epigenetic transcriptional regulation by cytosine methylation may have led to a selection for or against cytosines, involving negative or positive regulatory elements in the expression controlling U3 region. As shown here, class I and III retroviruses are especially rich in conserved cytosines in U3. The evolution of epigenetics will also have influenced the use of retrotransposon integrase chromodomains which bind to posttranslationally modified histones. In Ty3 it recognizes H3 methylated heterochromatin [10,13-15]. Furthermore, evolution of CpG methylation to silence LTR-driven transcription may have influenced U3 sequence diversity.

A feature of Sire LTRs is that part of the 5^′^end of U3 contains inverted repeats, different from SIRs, which together with complementary repeats outside of the LTR, upstream of PPT, form a probable stem loop with PPT exposed in the loop [32]. It was also found in HIV [42]. A systematic search for such PPT-containing hairpins in other LTR retroelements is warranted. Such a 3´terminal stem-loop is analogous to the U5-IR loop in the 5^′^end of the retroviral genome [43]. Stem loops involving base-pairing between LTR and LTR-adjacent sequences are of interest both from the aspect of LTR sequence conservation, but also of the origin of LTRs. It was shown that several chromoviruses use a 5′hairpin structure for priming, instead of a tRNA [44,45]. Moreover, DIRS RNA was postulated to use stem-loop structures for the same purpose [46]. It is uncertain if the terminal direct and indirect repeats found in Penelope elements, which seem to use target priming [47-49], may have been embryos of present-day LTRs. Both Penelope and DIRS elements do not have a DDE integrase. The presence of this integrase thus is not a prerequisite for their terminal repeats.

When only LTR retrotransposons are compared, LTR and Pol trees are in broad agreement (Figure 2) except that retroviruses cluster with a subset of *Metaviridae* in the LTR tree. If the LTR tree was an accurate representation of reality this would imply that *Metaviridae* is not a homogeneous clade. The occurrence of elements with inverted order of the RT and IN and reverse transcriptase priming support that *Metaviridae* has had a complex evolution. Another aspect is that the number of informative sites of the SuperViterbi alignment is limited, often less than 100. It is based on the match states of the constituent HMMs, of which some are almost invariable. Therefore, although the bootstrap support of the LTR-based trees indicated that they were robust, the fidelity of phylogenetic reconstruction from the HMMs must have limitations. Other arguments are:

First, according to the LTR tree, the rGmr1 clade is, together with the Sushi clade, basal to the other *Metaviridae* clades and retroviruses. The rGmr1 clade is unique among *Metaviridae* in having the same order between the RT and IN domains as *Pseudoviridae*[50]. This is consistent with rGmr1 branching off after *Pseudoviridae* but before the other *Metaviridae* and retrovirus clades as in the LTR tree (except for Sushi). rGMr1 is most similar to Osvaldo and Ulysses in the Pol trees.

Second, Llorens and colleagues [11], noted a close similarity between class III retroviruses and Errantiviruses (which consist of Zam and Gypsy *sensu stricto*, see Figure 2) by comparing the *gag* and *pro* genes of both groups. Furthermore, Mag and other non-chromoviral clades such as Micropia and Mdg3 of insects, and class II retroviruses (which include HMLs and Lentiviruses) have features in common in their *gag* and *pro* genes [11]. Altogether this is consistent with the sister relationship between retroviruses and some non-chromoviral *Metaviridae* clades.

Third, the weblogos of retroviral LTRs have more in common with some non-chromoviral *Metaviridae* clades than with Sushi and rGmr1, as noted above for class III retroviruses and Mag A. This is evident in the Gammaretroviral, the Zam and the Mdg1 weblogos with 300 match states (data not shown): They all contain long stretches based on CA or CAA in U3.

Why does the Pol tree of Figure 2 show a monophyletic *Metaviridae*? It could result from a summative effect of independently evolving RT, RH and IN modules. Alternatively, it could be the result of (artefactual) long-branch attraction between *Pseudoviridae* and retroviruses since both have long branches compared to Gypsy/Ty3 in Pol trees (see Figure 2). Long-branch attraction is well known to lead to inaccurate trees (see for example [51,52]) in the context of bird phylogenetics); it occurs when the mutation rate varies extensively between different clades.

The Pol and RT trees (Figures 2 and 3, and Additional file 2: Figure S4) indicate different phylogenies of retrotranscribing elements and viruses. The non-LTR using DNA viruses hepadna and caulimo are interspersed among the retrotransposons. This, and the existence of an R-U5 like structure in hepatitis B virus [53], create difficulties for a simplistic LTR and retrovirus phylogeny. It is not possible to claim monophyly of all retrotranscribing viruses and elements

In Llorens *et al*. [11], the authors proposed ‘the three kings hypothesis’ according to which the three classes of retroviruses originated from three *Metaviridae* ancestors. Their conclusions were based on Gag phylogenies and sequence elements in other proteins such as the flap motif embedded in the Pro coding region. The divergent results shown in Figures 2, 3 and 4, and Additional file 2: Figure S4, illustrate that when a retroelement is reconstructed results can differ, indicating that polymerase evolution was complex, with instances of rather drastic cross-element and host-element modular transfers. In a similar vein, a network hypothesis of LTR retrotransposon evolution was proposed [12]. However, all previously published Pol phylogenies [13], as well as phylogenies based on three independent trees of distinct Pol domains, support the monophyly of retroviruses. Our incomplete evidence from the LTR tree also indicates that retroviruses are monophyletic. On the other hand, the tree of Figure 3 indicates that the gamma, epsilon and spumaretroviruses are more related to *Metaviridae* than the other retroviruses are. More information is needed.

In the broader context of LTR retrotransposons, it is to be expected that different genes yield somewhat different tree topologies and as a consequence there is no single retroelement tree. Indications for a mosaic origin of LTR retroelements are the independent acquisitions of retroviral RNase H [19] and possibly also of the *Pseudoviridae* and rGmr1 IN, as suggested by their unique genomic position. The *Pseudoviridae* IN shares the HHCC and DDE motifs with retroviral and *Metaviridae* retroelements but also has a unique C terminal motif, the GKGY motif [9]. On the other hand, gammaretroviral and some *Metaviridae* INs (including chromoviruses) have the GPY/F motif in the IN C terminus [13]. The newly discovered Ginger 1 DNA transposon has a DDE integrase which seems more closely related to certain *Metaviridae* integrases [54] than to integrases from other *Metaviridae*, retroviruses or *Pseudoviridae*. It also has a GPY/F domain. This can be interpreted as supporting multiple origins for IN in LTR retrotransposons but it could also be due to an exchange in the other direction, that is, from *Metaviridae* to Ginger 1. It is interesting that Ginger 1 has terminal inverted repeats (TIRs), but not LTRs. Its TIRs begin with the sequence TGTNR which is close to the SIR TGTTRNR found in LTRs. Maybe LTRs arose from such TIRs. As mentioned above, the retroviral Gag is not monophyletic according to Llorens’ Gag phylogeny [11]. Another sign of Gag ancestry is the presence of CCHC zinc fingers in both Errantivirus Gag and capsid proteins of caulimoviruses [55].

A third explanation for the limited discrepancy between the RT- and LTR-based trees is the occurrence of a recombination event between a retrovirus and a non-chromoviral *Metaviridae* retrotransposon so that the retroviral LTRs are derived from the latter but the retroviral RT is not.

Based on RT similarity and a gradual acquisition of functionally important structures, we suggest a complex series of events during the evolution of LTR retrotransposons (Figure 3), highlighting the intertwined relation between LTR and non-LTR retrotransposons. A similar tree was earlier presented by [19]. A somewhat different branching order was seen in Additional file 2: Figure S4. These trees contain relatively few branches, and are not intended as ‘final’ phylogenetic reconstructions.

Although the exact sequence of events during retroviral evolution is difficult to unambiguously reconstruct at this stage, several lines of evidence can be drawn from sequence and structural similarities. The starting point of LTR retrotransposon evolution (Figure 4) may have been from non-LTR transposons related to LINE and Penelope elements. The latter have terminal repeats, which may have been precursors of LTRs. RH was acquired at least twice [19]. Because of the varying position of integrase relative to reverse transcriptase, several horizontal transfers of integrase, maybe involving a DNA transposon, are postulated. A hypothetical LTR retrotransposon precursor may have been self-priming, via a 5′ hairpin [45]. A similar mechanism has been proposed for DIRS retrotransposons [46]. Some chromoviruses still use hairpin priming. tRNA priming via the PBS seems to be a rather late event. Judging from the RT-based trees, *Pseudoviridae* seems to be the oldest LTR retrotransposon group, but the relation between their reverse transcriptases and those of non-LTR retrotransposons like DIRS, and of hepadna and caulimoviruses is uncertain. Other events during LTR retrotransposon genesis were acquisition of a capsid and nucleic acid binding protein (‘Gag’), a pepsin-related aspartic protease and a membrane glycoprotein. It is likely that further search in the rapidly expanding base of host genomic sequences will reveal other retroelement intermediates, which will clarify the complex sequence of events.

The selective pressures acting on the host species set the stage for the evolutionary scenario of retrotransposons. Both *Pseudoviridae* and *Metaviridae* are widespread in eukaryotes, while retroviruses are confined to vertebrates. It is likely that retroviral evolution started from a *Metaviridae* precursor, in an early vertebrate [12,45].

The prerequisites for the evolutionary assembly of LTRs are:

(1) The existence of an RNAse H coding region in the element along with its site of action, the PPT. RNAse H was apparently acquired twice during evolution, and from distinct sources, first in LINE elements, and later in retroviruses [19].

(2) A polymerase II (RNA Pol II) dependent promoter (which often involves a hairpin structure) in close proximity to a polyadenylation signal.

(3) Presence of an integrase. Perhaps a selection for a new type of integration guidance favoured the acquisition of a DDE integrase, in at least three separate events. Alternatively, since IN has a similar folding as RH [56], it is conceivable that it originally arose as a gene duplication of RH. The DDE integrase of the Ginger DNA transposon is highly similar to that of some gypsy elements [54]. The integrase was taken up in *pol*, just after the RT-RH sequence. However, a similar but separate acquisition must also have occurred in a precursor of *copia* and rGmr1 retroelements. In this case, the integrase may have been positioned before RT-RH. The order and direction of these sequence exchanges are uncertain.

(4) The use of tRNA priming through a PBS probably is a relatively late evolutionary event. It is likely that the progenitors of LTR retrotransposons used hairpin priming instead.

LTRs may have arisen from a complex sequence of contributions from several types of retrotranscribing elements and viruses. In addition, specific regulatory motifs probably accumulated in the U3 region in response to adaptive selection to allow tissue-tropic transcription and in response to CpG methylation. The close relationship between packaged (viral) and unpackaged ‘selfish nucleic acid’ based on RNA and DNA during retrotransposon evolution is remarkable. Although difficult to trace, both could have co-existed and exchanged structures during evolution of multicellular organisms.

## Conclusion

We have demonstrated that retroviruses and *Metaviridae* elements share the same conserved motifs but that *Pseudoviridae* elements differ slightly. Nearly all LTR retrotransposons, including plant *Metaviridae* and *Semotivirus* (Bel/Pao), which were not modelled in this study, have conserved SIRs. Some *Metaviridae* of *Drosophila* were however an exception. All investigated *Metaviridae* and retroviruses have a well-conserved AATAAA but a less conserved TATA box whereas the opposite is true for *Pseudoviridae* (Copia/Ty1) elements of plants, reflecting that the polyadenylation signal is less conserved in plants and demonstrating how well LTRs can mimic the promoters and regulatory elements of their hosts.

Surprisingly, conserved features other than promoter elements and the 5′ SIR are present in U3: Closely related LTRs such as Retrofit/Sire or Zam/Mdg1 have the same kind of low complexity regions in U3. The LTR alignments seem to favour paraphyly of *Metaviridae* and monophyly of retroviruses, agreeing partly with Llorens *et al*. [11].

As for retroviruses, the HMMs constructed here can also be used for detection of many groups of LTR retrotransposons if they are combined with detection of other motifs as is done by the RetroTector^©^ program [57,58]. Implementation of large-scale parallel execution of HMM detection is required, because of speed limitations of HMM algorithms.

## Methods

Reference sequences from *Metaviridae* (Gypsy/Ty3) and *Pseudoviridae* (Copia/Ty1) were collected from Genbank, following Llorens *et al*. [12]. In addition, all available Gypsy/Ty3 and Copia/Ty1 sequences were retrieved from RepBase [5]. All class III retroviral sequences were obtained from RepBase.

The internal coding parts of all reference and all RepBase sequences were clustered by means of BLASTP and the CLANS software [59]. E values <1E-200 were chosen in order to produce as many groups as possible. This resulted in 14 well-separated clusters for Gypsy/Ty3. The coding sequences of Copia/Ty1 fell into two main groups that could be further subdivided into a total of five groups. For each group the corresponding LTRs were selected. This assumes that LTRs and coding retrotransposon genes have co-evolved, which may often be the case as suggested by Benachenhou *et al*. [22].

HMMs were constructed for each LTR group, which was divided into a training set and a test set containing approximately 80/20% of the LTRs, respectively. The HMMs were selected based on score with the test set and/or presence of conserved motifs in the corresponding alignments. In some cases it was necessary to subdivide the coding sequence clusters to fulfil our HMM selection criteria. For example our Zam HMM describes only a subclade of Errantiviruses. The HMMs were used for detection in chromosomes from four different organisms: *Drosophila melanogaster*, *Anopheles gambiae*, *Danio rerio* and *Oryza sativa*. For comparison, RepeatMasker was run on each chromosome using the RepBase library version 090604.

The HMM algorithms were implemented in C by Panu Somervuo and FB. The software for detection was parallelised using Message Passing Interface (MPI), and run on a cluster of computers with 22 nodes. By parallelization the execution times could be reduced to a few hours for a genome size of 70 Mbp instead of 2 to 3 days. Other software used were ClustalW [40], Mega version 4.1 [60] for phylogenetic trees, and Bioedit [61] and Weblogo [62] for visualisation of alignments. Phylogenetic trees were either neighbour-joining, maximum likelihood or minimum evolution, with bootstrap values from 1,000, 500 and 1,000 replications, respectively.

As described under ‘model building’ above, the profile HMM system cannot accommodate large variations in LTR length. It presupposes a certain number of match states. However, as described we systematically tested many different match states before settling for an optimal HMM, and therefore this source of bias was minimised.

## Availability of supporting data

Additional file figures and HMM alignments are in Additional file 2.

HMM training sets and *Metaviridae*/*Pseudoviridae* clusters are detailed in Additional file 1.

## Abbreviations

ERV: Endogenous retrovirus; gag: Group antigen gene, encoding structural proteins; Gag: Group antigen protein; GPY/F domain: A portion of the integrase C-terminal domain; HIV: Human immunodeficiency virus; HERV: Human endogenous retrovirus; HML: Human MMTV-like sequence; HMM: Hidden Markov model; ICTV: International Commission for Taxonomy of Viruses; INR: Initiator of transcription; IN, INT: Integrase; IR: Inverted repeat; LTR: Long terminal repeat; MMTV: Mouse mammary tumour virus; PAS: Polyadenylation site; PBS: Primer binding site; Pol: Polymerase protein; PRO: Protease domain; PPT: Polypurine tract; R: Repeat portion of LTR; RH: Rnase H; RT: Reverse transcriptase; SIR: Short inverted repeat; TIR: Terminal inverted repeat; TSD: Target site duplication; TSS: Transcriptional start site; U3: Unique 3^′^ LTR portion; U5: Unique 5^′^ LTR portion; XRV: Exogenous retrovirus.

## Competing interests

The authors have no competing interests.

## Authors' contributions

FB programmed the HMMs, conducted the experiments and participated in the writing. JB conceived of the paper, guided the development of the work, arranged funding and participated in the writing. GA and EBR participated in the development of the work, arranged funding and participated in the writing. GS participated in the development of the work and in the writing. JDB participated in the development and in the writing. All authors read and approved the final manuscript.

## Supplementary Material

Additional file 1: Table S1 Constituents of the internal coding sequence clusters for *Metaviridae* and *Pseudoviridae* as generated by the CLANS software. Table S2. Constituents of the training set for the LTR HMMs presented in this paper, and the previous paper (Benachenhou *et al*. [21]). Click here for file

Additional file 2: Figure S1 Long weblogo of Sire. Long weblogo for a Viterbi alignment of the Sire training set. Conventions as in Figure 1. Figure S2. Long weblogo of Gamma. Long weblogo for a Viterbi alignment of the Gamma training set. Conventions as in Figure 1. Figure S3. Long weblogo of class III retroviruses. Long weblogo for a Viterbi alignment of the training set of class III retroviruses. Conventions as in Figure 1. Figure S4. Alternative minimum evolution tree of reverse transcriptases of retrotranscribing elements and viruses. DNA viruses are shown in magenta. DHV, Duck Hepatitis Virus; MLV,_Mouse Leukemia Virus. GenBank ID numbers are also given. Supplementary HMM alignments. Alignments for the HMM training sets, detailing match and insert states. Click here for file
