# Supplementary material for: Conserved structure and inferred evolutionary history of long terminal repeats (LTRs)
Source: Mob DNA. 2013 Feb 1;4:5. doi: 10.1186/1759-8753-4-5 (PMC3601003; doi:10.1186/1759-8753-4-5)
Supplement: Additional file 1: Table S1 — Constituents of the internal coding sequence clusters for Metaviridae and Pseudoviridae as generated by the CLANS software. Table S2. Constituents of the training set for the LTR HMMs presented in this paper, and the previous paper (Benachenhou et al. [21]). [file 1759-8753-4-5-S1.pdf]

The last two letters refer to host species,
see abbreviations at the bottom of the table

Zam

Name	Length
GYPSY43_LTR_AG	196
TED_LTR	273
GYPSY41_LTR_AG	311
GYPSY40_LTR_AG	167
GYPSY39_LTR_AG	353
GYPSY42_LTR_AG	251
PIFO_LTR	444
GYPSY8_LTR_DYA	337
Median length	292

Mag C

Name	Length
GYPSY-7_LTR_HM	305
GYPSY-16_LTR_HM	136
GYPSY58_LTR_DR	144
GYPSY143_LTR_DR	148
GYPSY14_LTR_SP	118
GYPSY1_LTR_SP	159
GYPSY6_LTR_SP	160
GYPSY-15_LTR_HM	86
GYPSY3_LTR_SP	168
GYPSY55_LTR_DR	240
GYPSY141_LTR_DR	197
GYPSY21_LTR_SP	180
GYPSY68_LTR_DR	190
GYPSY93_LTR_DR	249
GYPSY12_LTR_SP	125
GYPSY53_LTR_DR	222
Median length	164

Mag A

GYPSY59_LTR_AG	288
GYPSY58_LTR_AG	289
GYPSY28_LTR_AG	219
GYPSY19_LTR_AG	136
GYPSY66_LTR_AG	197
GYPSY57_LTR_AG	296
GYPSY24_LTR_AG	192
GYPSY64_LTR_AG	187
GYPSY23_LTR_AG	162
GYPSY25_LTR_AG	234
GYPSY62_LTR_AG	178
GYPSY3_LTR_AG	141
GYPSY69_LTR_AG	441
GYPSY21_LTR_AG	167
GYPSY60_LTR_AG	220
GYPSY67_LTR_AG	236
GYPSY63_LTR_AG	144
GYPSY68_LTR_AG	108

GYPSY20_LTR_AG	270
GYPSY65_LTR_AG	140
Median length	195
**CsRN1**	
GYPSY70_LTR_AG	355
GYPSY2_LTR_AG	368
GYPSY1-NVI_LTR	259
GYPSY1_LTR_AP	321
GYPSY53_LTR_AG	300
GYPSY48_LTR_AG	374
GYPSY9_LTR_AP	385
BOUDICCA_LTR	327
GYPSY11-NVI_LTR	306
GYPSY2_LTR_AP	407
GYPSY1_LTR_AG	146
GYPSY-29_LTR_NVI	409
GYPSY52_LTR_AG	393
Median length	355
**Sushi**	
GYPSY70_LTR_DR	640
GYPSY72_LTR_DR	596
GYPSY76_LTR_DR	460
GYPSY107_LTR_DR	770
GYPSY105_LTR_DR	782
GYPSY62_LTR_DR	475
GYPSY1_LTR_GA	527
GYPSY4_LTR_AO	669
CGRET_LTR	544
GYPSY5_LTR_DR	458
GYPSY89_LTR_DR	559
GYPSY96_LTR_DR	816
GYPSY103_LTR_DR	819
GYPSY88_LTR_DR	521
MARY1_LTR	426
GYPSY104_LTR_DR	870
GYPSY-115_LTR_DR	378
GYPSY-170_LTR_DR	898
GYPSY145_LTR_DR	311
GYPSY164_LTR_DR	381
GYPSY159_LTR_DR	458
GYPSY69_LTR_DR	408
GYPSY138_LTR_DR	137
GYPSY64_LTR_DR	835
Median length	536
**rGmr1**	
GYPSY-34_LTR_DR	960
GYPSY-167_LTR_DR	878
GYPSY90_LTR_DR	385
GYPSY47_LTR_DR	969
GYPSY123_LTR_DR	320

GYPSY-171_LTR_DR	824
GYPSY41_LTR_DR	890
GYPSY48_LTR_DR	967
GYPSY44_LTR_DR	699
GYPSYDR1_LTR	387
GYPSY36_LTR_DR	390
GYPSY-116_LTR_DR	738
GYPSY49_LTR_DR	360
GYPSY-169_LTR_DR	306
GYPSY-24_LTR_SP	1023
GYPSY38_LTR_DR	416
GYPSY40_LTR_DR	936
GYPSY43_LTR_DR	794
GYPSY139_LTR_DR	399
GYPSY46_LTR_DR	331
GYPSY-33_LTR_DR	1080
GYPSY120_LTR_DR	1036
GYPSY121_LTR_DR	327
GYPSY91_LTR_DR	294
GYPSY-32_LTR_DR	1160
GYPSOL_LTR	692
GYPSY37_LTR_DR	372
GYPSY124_LTR_DR	623
GYPSY155_LTR_DR	780
GYPSY17_LTR_SP	810
GYPSY-117_LTR_DR	748
GYPSY-31_LTR_DR	954
GYPSY45_LTR_DR	898
GYPSY35_LTR_DR	900
Median length	764

Abbreviations

AG: Anopheles Gambiae
DYA: Drosophila yakuba
HM: Hydra magnipapillata
DR: Danio rerio
SP: Strongylocentrotus purpuratus
NVI: Nasonia vitripennis
AP: Acyrthosiphon pisum
GA: Gasterosteus aculeatus
AO: Aspergillus oryzae

These are the Metaviridae (gypsy/Ty3) clusters based on Pol similarity, as described in the main text.

CsRN1

GYPSY49-I_AG_2
GYPSY52-I_AG_1
GYPSY70-I_AG_2
GYPSY2-I_AG
GYPSY1-I_AG_2
GYPSY48-I_AG_2
boudicca_I
SACI-7_2
Gypsy9-I_AP_2
Gypsy1-I_AP
Chimpo_I_2
GYPSY53-I_AG_1
Gypsy-29-I_NVi_2
Gypsy16-I_SP
Gypsy7-I_AP
Gypsy-25-I_NVi
Gypsy1-NVi_I
Gypsy2-I_AP
Gypsy11-NVi_I_2

mdg1_AG

GYPSY10-I_AG_2
GYPSY9-I_AG_2
GYPSY13-I_AG_2
GYPSY17-I_AG_2
GYPSY16-I_AG_2
GYPSY14-I_AG_2
GYPSY8-I_AG_1
GYPSY12-I_AG_2
GYPSY11-I_AG_2
GYPSY15-I_AG_1

rGmr1

Gypsy39-I_DR
Gypsy-169-I_DR
Gypsy44-I_DR
Gypsy-34-I_DR
Gypsy47-I_DR
Gypsy40-I_DR
Gypsy37-I_DR
Gypsy43-I_DR
Gypsy-24-I_SP
Gypsy-116-I_DR
GYPSYDR1
Gypsy148-I_DR
Gypsy-117-I_DR
Gypsy48-I_DR
rGmr1_I
Gypsy90-I_DR
Gypsy41-I_DR
Gypsy46-I_DR

Gypsy139-I_DR_2
Gypsy36-I_DR
Gypsy-171-I_DR
Gypsy-33-I_DR
Gypsy38-I_DR
Gypsy-167-I_DR_2
Gypsy127-I_DR
Gypsy45-I_DR
Gypsy35-I_DR
Gypsy-32-I_DR
Gypsy152-I_DR_2
Gypsy50-I_DR
Gypsy49-I_DR
Gypsy155-I_DR
Gypsy122-I_DR
Gypsy120-I_DR
GYPSOL_I
Gypsy121-I_DR
Gypsy123-I_DR
Gypsy2-I_SP
Gypsy161-I_DR
Gypsy124-I_DR
Gypsy-31-I_DR
Gypsy91-I_DR
Gypsy42-I_DR
Gypsy17-I_SP
Gypsy119-I_DR_2
Gypsy128-I_DR

Athila
GYPSO_I
Gypsy7-VV_I_1
Gypsy19-VV_I
DIASPORA_I
Gypsy1-PTR_I
POPGY1_I
Gypsy-34_SB-I
ATHILA6B_I
Gypsy11-VV_I
Gypsy6-VV_I_2
ATHILA4C_I_1
Gypsy2-PP_I_2
Gypsy18-PTR_I_2
Gypsy12-VV_I
Gypsy20-VV_I_2
Gypsy13-VV_I_2
ATHILA6C_I_1
Gypsy17-VV_I_2

Errantivirus
ted_I
GYPSY40-I_AG_2
Pifo_I_2
GYPSY39-I_AG_2
176_I

zam_I
297_I
GYPSY5_I_2
Gypsy8-I_Dya_2
idefix_I
GYPSY47-I_AG_2
yoyo_I
GYPSY44-I_AG_2
Gypsy7-I_Dmoj
GYPSY45-I_AG_1
GYPSY7-I_AG_2
tom_I
Gypsy2-I_DM_2
GYPSY42-I_AG_2
Gypsy20-I_Dya
burdock_I
HMS_beagle_I
GYPSY41-I_AG_3
GYPSY43-I_AG_1
Chouto_I_2
tv1_I
Gypsy1-I_DM_2
Gypsy_6B_3
Gypsy17-I_Dpse_1
gypsyvir_I
springer_I
Gypsy10-I_Dpse
Gypsy17-I_Dpse_3
Gypsy6-I_Dpse
Gypsy12-I_Dpse_1
Gypsy16-I_Dya
Gypsy11-I_Dya_1

mag A-clade
GYPSY22-I_AG
Gypsy56-I_DR
GYPSY19-I_AG
Gypsy153-I_DR_2
GYPSY20-I_AG
GYPSY28-I_AG
Gypsy142-I_DR
DRM_I
Gypsy67-I_DR
GYPSY23-I_AG
GYPSY27-I_AG
Gypsy3_MH-I
GYPSY26-I_AG
CFG1_I
Gypsy10-NVi_I
GYPSY6-I_CB
Gypsy158-I_DR
GYPSY63-I_AG
Gypsy-14-I_HM
Gypsy-9-I_HM
GYPSY67-I_AG
hydra_I

Gypsy92-I_DR
GYPSY64-I_AG
GYPSY55-I_AG
GYPSY3-I_AG
GYPSY21-I_AG
Gypsy-17-I_HM
GYPSY58-I_AG_2
GYPSY25-I_AG
GYPSY24-I_AG
NONAUT-5
GYPSY59-I_AG
Gypsy-28-I_NVi
Gypsy12-NVi_I
GYPSY60-I_AG_2
Gypsy163-I_DR_1
Gypsy13-NVi_I_2
Gypsy2_MH-I_2
NONAUT-3
GYPSY5-I_CB_1

mag C-clade

Gypsy5-I_SP
Gypsy86-I_DR
GYPS1_I
Gypsy143-I_DR
Gypsy-15-I_HM
Gypsy6-I_SP
Gypsy21-I_SP
Gypsy93-I_DR
Gypsy141-I_DR_2
Gypsy58-I_DR
Gypsy1-I_SP
Gypsy53-I_DR
Gypsy-16-I_HM
Gypsy-7-I_HM
Gypsy11-I_SP
Gypsy55-I_DR
Gypsy19-I_SP
Gypsy3-I_SP
Gypsy14-I_SP
Gypsy12-I_SP
Gypsy68-I_DR

mdg3

Gypsy3-I_AP
Gypsy6-I_AP
GYPSY32-I_AG
GYPSY36-I_AG
GYPSY33-I_AG
GYPSY38-I_AG
GYPSY37-I_AG
GYPSY35-I_AG
Gypsy21-I_Dpse
GYPSY29-I_AG
GYPSY5-I_AG

blastopia_I
GYPSY6-I_AG
mdg3_I
INVADER1-I_AG
Gypsy19-I_Dpse
Gypsy22-I_Dpse_1
Gypsy18-I_Dpse
Gypsy12-I_Dpse_3
Gypsy20-I_Dpse
Gypsy19-I_Dya
Gypsy22-I_Dpse_2
Gypsy12-I_Dya
Gypsy15-I_Dya
GYPSY4-I_AG
Gypsy2-I_Dya
Gypsy2-I_Dpse
Gypsy1-I_Dya_1
Gypsy14-I_Dpse_2

danio

Gypsy98-I_Dr_2
Gypsy157-I_DR_3
Gypsy59-I_DR
Gypsy10-I_DR
Gypsy-21-I_DR_1
Gypsy-17-I_DR_2
Gypsy-15-I_DR_2
Gypsy-16-I_DR_2
Gypsy-19-I_DR_1
Gypsy20-I_SP_2
Gypsy6-I_DR_1
Gypsy52-I_DR
Gypsy144-I_DR_2
Gypsy82-I_Dr_2
Gypsy13-I_DR_1
Gypsy8-I_DR_1
Gypsy100-I_DR_2
Gypsy-18-I_DR_1
Gypsy12-I_DR_2
Gypsy10-I_SP_2
Gypsy-14-I_DR_1
Gypsy151-I_DR
Gypsy63-I_DR
Gypsy149-I_DR
GYPS2_I
Gypsy4-I_DR_2
Gyp_MD_I
Gypsy134-I_DR_2
Gypsy78-I_DR_2
Gypsy65-I_DR_2
Gypsy156-I_DR_2
Gypsy146-I_DR_2
Gypsy84-I_DR
Gypsy66-I_DR_2
Gypsy118-I_DR_2
Gypsy106-I_DR_2

Gypsy85-I_DR_2
Gypsy81-I_Dr_2
Gypsy71-I_DR_2
Gypsy137-I_DR_2
Gypsy8-I_SP_2
Gypsy136-I_DR_2
Gypsy75-I_DR_2
Gypsy80-I_DR_2
Gypsy94-I_DR_2
Gypsy147-I_DR_2
Gypsy60-I_DR_2
Gypsy132-I_DR_2
Gypsy102-I_DR_2
Gypsy140-I_DR_2
Gypsy83-I_DR_2
Gypsy74-I_DR_2
Gypsy54-I_DR_2
Gypsy117-I_DR_2
Gypsy51-I_DR_2
Gypsy133-I_DR_1
Gypsy57-I_DR_1
Gypsy116-I_DR_1
Gypsy116-I_DR_2
Gypsy133-I_DR_2
Gypsy144-I_DR_3
Gypsy57-I_DR_2
Gypsy94-I_DR_3

tat
Gypsy-14_SB-I_3
ATLANTYS1_I_1
Gypsy3-VV_I_2
Ogre-PT3_I_2
Gypsy-18_SB-I_2
Gret1_I
Ogre-VP1_I_2
GYPSI_I_2
Ogre-PT2_I_2
Gypsy-30_SB-I_2
Gypsy-71-I_ZM
Gypsy-10_SB-I
GYPSIA_I_3
ATLANTYS_LC_I_3
Gypsy3-SB_I_2
Ogre-LE1_I_2
GmOgre_I_2
Ogre-MT3_I_2
Gyp_I_MT_2
retrosor1_I
Ogre-MT4_I_2
GYPSY3-I_MT_2
Gypsy27-ZM_I
Gypsy12-ZM_I_3
Ogre-SD1_I_2
Gypsy-22_SB-I
Ogre-PT1_I_2

RETRO2_I
RETRO2A_I_2
Gypsy2-SB_I
Gypsy29-ZM_I_2
Gypsy-3-I_TA
Gypsy11-ZM_I
CALYPSHAN2_I_MT_2
CINFUL2A_I
cinful1_I
Gypsy-74-I_ZM
Atlantys-2-I_OS_2
Gypsy16-VV_I_2
HUCK1-I_ZM_2
Gypsy-92_SB-I_2
Gypsy-2-I_TA_2
Gypsy-2-I_TA_1

ulysses

Gypsy4-I_Dpse
Gypsy16-I_Dpse_1
GYPSY12_I_1
Gypsy10-I_Dya
Gypsy3-I_Dpse
Gypsy7-I_Dpse_2
Gypsy5-I_Dpse_2
Gypsy15-I_Dpse_2
Gypsy13-I_Dpse_2
Gypsy24-I_Dpse
Gypsy5-I_Dya_2
Gypsy23-I_Dpse_1
ulysses_I
Gypsy18-I_Dya
Gypsy13-I_Dya_3
Gypsy23-I_Dpse_2
Gypsy6-I_Dya_3

osvaldo

Gypsy11-I_Dpse_2
Gypsy6-I_Dmoj_2
Gypsy8-I_Dpse_2
osvaldo_I
Gypsy-38_NVi-I_2
Gypsy-17-I_NVi_2
Gypsy15-NVi_I_2
Gypsy-16-I_NVi_2
Gypsy1-I_Dmoj_1
Gypsy4-I_Dmoj
Gypsy-23-I_NVi_2
Gypsy-19-I_NVi_2
Gypsy-27-I_NVi_2
Gypsy-21-I_NVi_3
Gypsy4-I_Dya_3
Gypsy4-I_Dya_2
Gypsy9-I_Dpse

Chromovirus
REM1_I
GYMAG1_I_2
GYMAG2_I_3
Gypsy-29_SB-I
Gypsy-25_SB-I_2
GYPSOR1_I_2
Gypsy3-I_VC
Gypsy-6_SB-I
Gypsy-19_SB-I
Gypsy30-ZM_I
RETROSAT2_I
Gypsy-66_SB-I_2
Gypsy-77-I_ZM_2
Gypsy-4-I_TA
Gypsy63-ZM_I_2
Gypsy65-ZM_I
SUKKULA4_HV_I
RETROSAT3_I
RAM9B_I
Gypsy32-ZM_I
Gypsy-12_SB-I
TCG3_I_2
RETROSAT5_I_2
RIRE3_I_2
RAM12_I_2
RETROSAT4_I
Gypsy-78-I_ZM_2
Gypsy14-PTR_I_2
Gypsy67-ZM_I_2
tse3_I
Gypsy-70-I_ZM_2
ROMANI1_I
Gypsy6-PTR_I
Gypsy47-ZM_I
Gypsy26-ZM_I
Gypsy6-I_VC
Gypsy5-PTR_I
Gypsy1-SB_I_2
SZ-56A_I
Gypsy-104_SB-I
Gypsy-82_SB-I
Gypsy6-ZM_I
Gypsy14-ZM_I
Gypsy-16_SB-I
Gypsy2-PTR_I
Gypsy19-I_CR
Gypsy-78_SB-I
Gypsy-42_SB-I
Gypsy-56_SB-I
Gypsy10-I_VC
GYPSY1GM_I
Gypsy-28-I_DR
Gypsy-114_SB-I
Gypsy15-ZM_I
DEA1

Gypsy64-I_DR_2
Gypsy15-I_CR_2
Gypsy105-I_Dr_2
Gypsy103-I_Dr
Gypsy-36_SB-I
SZ-59_I
SZ-10_I
RETROSAT6_I_2
GYPSHAN3_I_MT
Gypsy2-VV_I
Gypsy-55_SB-I
Gypsy-113_SB-I_2
GmGYPSY10_I
Gypsy-17_SB-I
Gypsy-77_SB-I
Gypsy-70_SB-I
Gypsy21-ZM_I
Gypsy19-PTR_I_2
Gypsy104-I_Dr_2
Gypsy-7_SB-I
Gypsy-33_SB-I
CRM_INT
CRM_I
Gypsy7-I_VC_1
Gypsy55-ZM_I
Gypsy-39_SB-I
Gypsy-24_SB-I
Gypsy16-ZM_I
Gypsy-57_SB-I
Gypsy-47_SB-I
CRM-I_OS
Gypsy20-PTR_I
Gypsy16-PTR_I
Gypsy-13_SB-I_1
Gypsy41-ZM_I
Gypsy-41_SB-I
Gypsy5-ZM_I
Gypsy-62_SB-I
Gypsy-37_SB-I
Gypsy24-ZM_I
Gypsy-60_SB-I
Gypsy8-ZM_I_1
Gypsy-75_SB-I_1
Gypsy-69_SB-I
Gypsy62-ZM_I
Gypsy24-PTR_I
Gypsy-65_SB-I
ATGP3B_I
Gypsy-72_SB-I
GYLES1_I
SHAGY_I_MT_1
RIRE8C_I
Gypsy-64_SB-I
Gypsy-28_SB-I
SZ-54B_I
Gypsy8-PTR_I

Gypsy-35_SB-I
GYPSODE1_I
Gypsy96-I_DR_2
Gypsy-32_SB-I_1
Gypsy-21_SB-I_2
Gypsy-165-I_DR_2
Gypsy-46_SB-I
Gypsy15-PTR_I
Gypsy-48_SB-I
Gypsy7-PTR_I
Gypsy23-PTR_I
Gypsy62-I_DR
Gypsy-76_SB-I
Gypsy61-ZM_I
Gypsy-40_SB-I
Gypsy-170-I_DR_2
Gypsy50-ZM_I
Gypsy16-I_VC
Gypsy-1-I_CR_2
sushi_I
Gypsy1-I_GA
Gypsy-51_SB-I
Gypsy-106_SB-I_1
ATGP4_I
SZ-54D_I
GYZMA1_I
Gypsy-8_SB-I
Gypsy-71_SB-I
CRMA1_I_2
Gypsy8-VV_I
Gypsy60-ZM_I
Gypsy-15_SB-I
Gypsy17-PTR_I
Gypsy107-I_Dr_2
Gypsy-23_SB-I
Gypsy3-PTR_I
Gypsy4-I_AO
Gypsy3-ZM_I
Gypsy-31_SB-I
Gypsy-117_SB-I
Gypsy10-PTR_I
Gypsy-59_SB-I
Gypsy-115-I_DR_2
Gypsy101-I_DR
Gypsy-84_SB-I
ATGP5A_I_2
Gypsy159-I_DR
Gypsy-44_SB-I
Gypsy8-I_VC_1
Gypsy70-I_DR_2
Gypsy1_ZM_I_2
Gypsy14-I_VC
Gypsy-50_SB-I
Gypsy5-I_VC
Gypsy1-I_ST
Gypsy88-I_DR_2

Gypsy-9_SB-I
GYPSHAN4_I_MT
GYPOT1_I
GYCUME1_I
Gypsy19-ZM_I
ATGP3A_I_2
Gypsy97-I_DR_2
Gypsy76-I_DR_2
Gypsy-97_SB-I
SZ-54A_I
Gypsy23-ZM_I_2
Gypsy-38_SB-I_1
SZ-54C_I
Gypsy11-PTR_I
GYPSY5I_DR
Gypsy7-ZM_I
Gypsy69-I_DR
Gypsy37-ZM_I
Gypsy-94_SB-I
Gypsy-99_SB-I
Gypsy-53_SB-I
Gypsy89-I_DR
Gypsy-166-I_DR_2
Gypsy-102_SB-I
GYZMA2_I
Gypsy22-VV_I_1
Gypsy-105_SB-I_2
Gypsy2-I_ST
Gypsy-68_SB-I
Gypsy-168-I_DR_2
Gypsy135-I_DR_2
Gypsy-85_SB-I
Cgret_I
Gypsy-111_SB-I
Gypsy-110_SB-I_2
Gypsy-58_SB-I_2
Gypsy-54_SB-I
IBGYPSY1_I_1
Gypsy138-I_DR
Gypsy-26_SB-I
Gypsy22-PTR_I
Gypsy-79_SB-I
Gypsy-81_SB-I
Maggy_I
Gypsy108-I_Dr_2
Gypsy-93_SB-I_1
Skippy_I
Gypsy-49_SB-I_2
Gypsy-103_SB-I
GYARLI1_I_2
Gypsy72-I_DR
Gypsy-20_SB-I
MarY1_I
Gypsy-118_SB-I_2
Gypsy145-I_DR
Gypsy-52_SB-I_1

Gypsy15-I_VC
Gypsy-63_SB-I
Gypsy15-VV_I
AFLAV_I_2
Gypsy164-I_DR
Gypsy-67_SB-I_1
Gypsy20_ZM_I_1
Gypsy5-VV_I
Gypsy2-I_AO_2
Gypsy33-ZM_I_1
MGLR3_I
Gypsy40-ZM_I_2
CfT1_I
Gypsy-11_SB-I_1
grasshopper_I
Gypsy4-ZM_I_1
Gypsy11-I_VC_2
Gypsy56-ZM_I_2
REALAA_I
Real_I
Gypsy-74_SB-I_1
Gypsy4-I_VC_2
Gypsy18-VV_I
Gypsy-1-I_ACa_2
Gypsy-27_SB-I
Gypsy39-ZM_I_2
Gypsy57-ZM_I
Gypsy17-I_CR_2
Gypsy25-ZM_I_2
Gypsy-109_SB-I
Gypsy2-ZM_I_1
Gypsy4-PTR_I_1
Gypsy-61_SB-I_1
Gypsy-107_SB-I
SZ-64B_I_2
Gypsy-87_SB-I_2
Gypsy3-PP_I
Gypsy9-PTR_I_1
Gypsy18-ZM_I_2
Gypsy-43_SB-I_1
CAREP1
Gypsy1-VV_I_1
RN12_I
Gypsy-96_SB-I_2
Gypsy-98_SB-I
Gypsy-86_SB-I_2
Gypsy14-I_CR
Gypsy-91_SB-I_2
Gypsy-101_SB-I_2
Gypsy18-I_CR_2
Gypsy-45_SB-I
Gypsy-89_SB-I_1
Gypsy-88_SB-I_1
Gypsy43-ZM_I
Gypsy-112_SB-I
Gypsy-83_SB-I_1

Gypsy-119_SB-I_2
Gypsy-100_SB-I
Gypsy-83_SB-I_2
Gypsy12-PTR_I
Gypsy10-ZM_I_3
Gypsy-101_SB-I_1
Gypsy28-ZM_I
GYPSHAN2_I_MT_1
Gypsy9-ZM_I_2
Gypsy17-ZM_I_1
Gypsy-86_SB-I_1
Gypsy-43_SB-I_2
Gypsy-87_SB-I_1
Gypsy-91_SB-I_1
Gypsy10-VV_I
Gypsy51-ZM_I_1
SZ-64B_I_1
Gypsy2-ZM_I_2
Gypsy-90_SB-I
Gypsy58-ZM_I_2
Gypsy51-ZM_I_2
Gypsy-116_SB-I_3
Gypsy9-ZM_I_1
Gypsy13-ZM_I_2
Gypsy4-I_VC_1
Gypsy-89_SB-I_2
Gypsy-80_SB-I_2
Gypsy1-VV_I_2

Nvi

Gypsy7-NVi_I_1
Gypsy14-NVi_I
Gypsy-24-I_NVi_1
Gypsy-20-I_NVi_2
Gypsy-34_NVi-I_2
Gypsy-31-I_NVi_1
Gypsy-30-I_NVi_3
Gypsy-37_NVi-I_2
Gypsy-18-I_NVi
Gypsy-36_NVi-I_2
Gypsy-26-I_NVi_1
