# Supplementary material for: Conserved structure and inferred evolutionary history of long terminal repeats (LTRs)
Source: Mob DNA. 2013 Feb 1;4:5. doi: 10.1186/1759-8753-4-5 (PMC3601003; doi:10.1186/1759-8753-4-5)
Supplement: Additional file 2: Figure S1 — Long weblogo of Sire. Long weblogo for a Viterbi alignment of the Sire training set. Conventions as in Figure 1. Figure S2. Long weblogo of Gamma. Long weblogo for a Viterbi alignment of the Gamma training set. Conventions as in Figure 1. Figure S3. Long weblogo of class III retroviruses. Long weblogo for a Viterbi alignment of the training set of class III retroviruses. Conventions as in Figure 1. Figure S4. Alternative minimum evolution tree of reverse transcriptases of retrotranscribing elements and viruses. DNA viruses are shown in magenta. DHV, Duck Hepatitis Virus; MLV,_Mouse Leukemia Virus. GenBank ID numbers are also given. Supplementary HMM alignments. Alignments for the HMM training sets, detailing match and insert states. [file 1759-8753-4-5-S2.pdf]

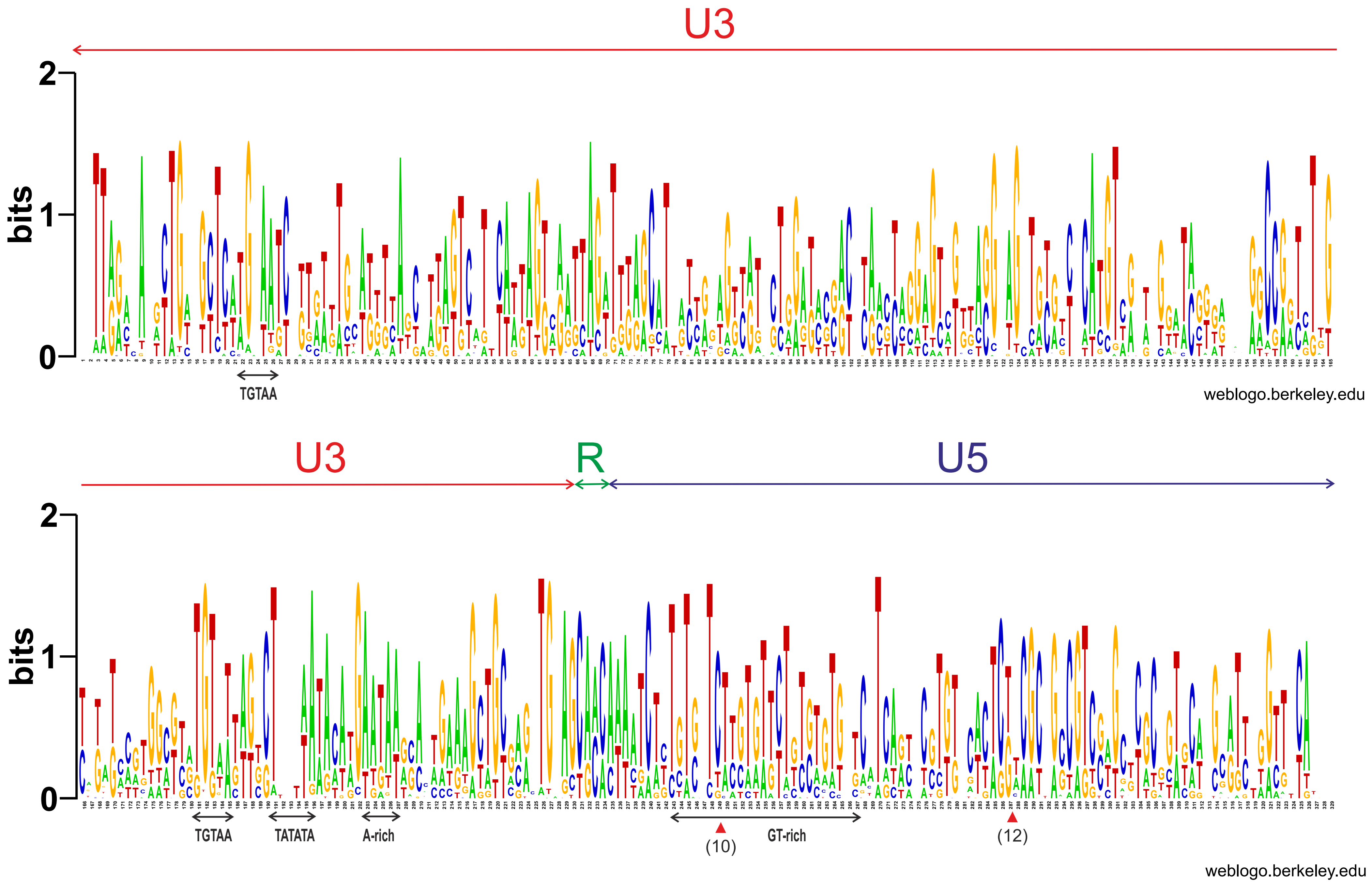

Suppl fig 1, long Sire HMM

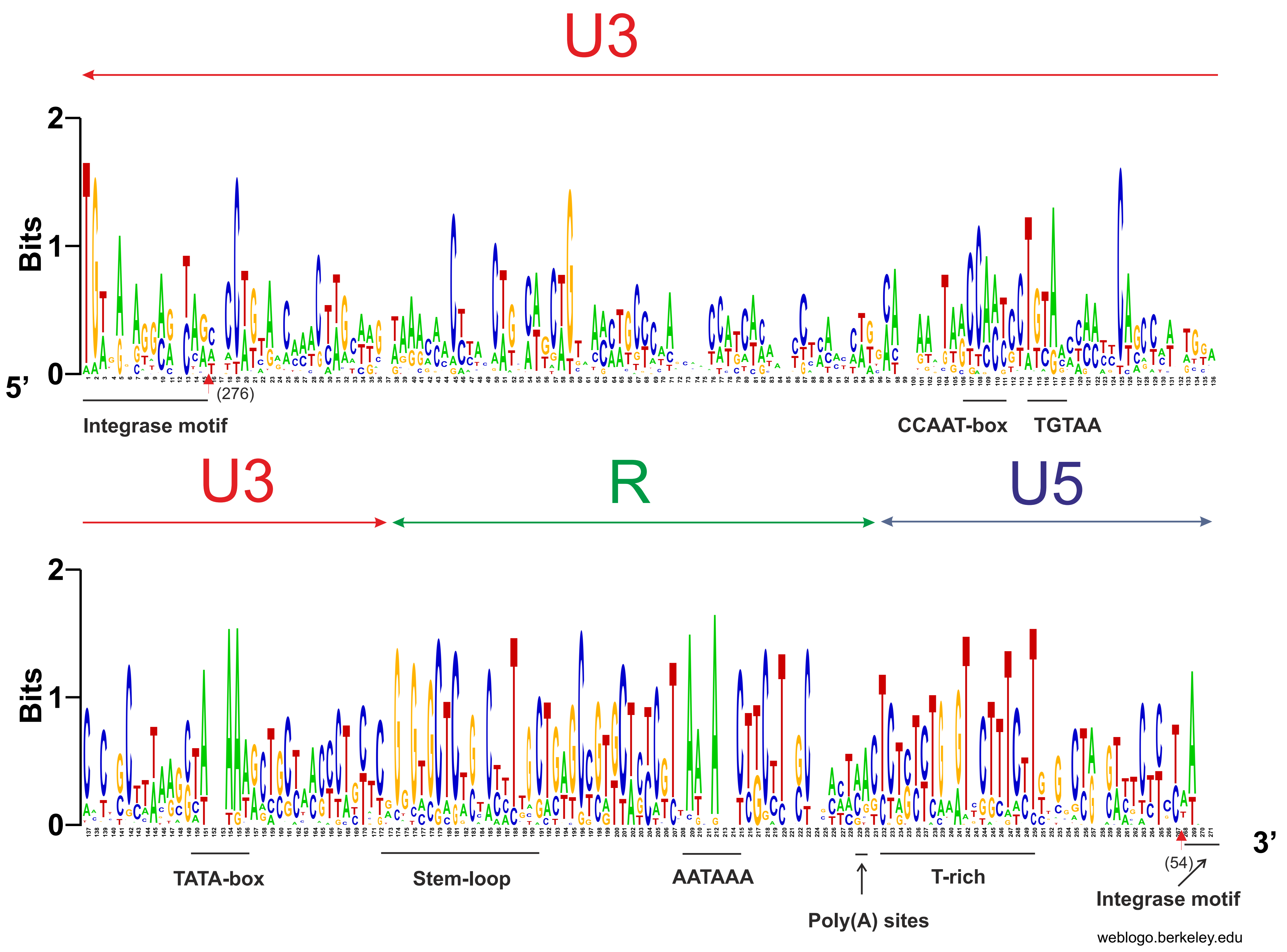

Suppl fig 2, long Gamma HMM

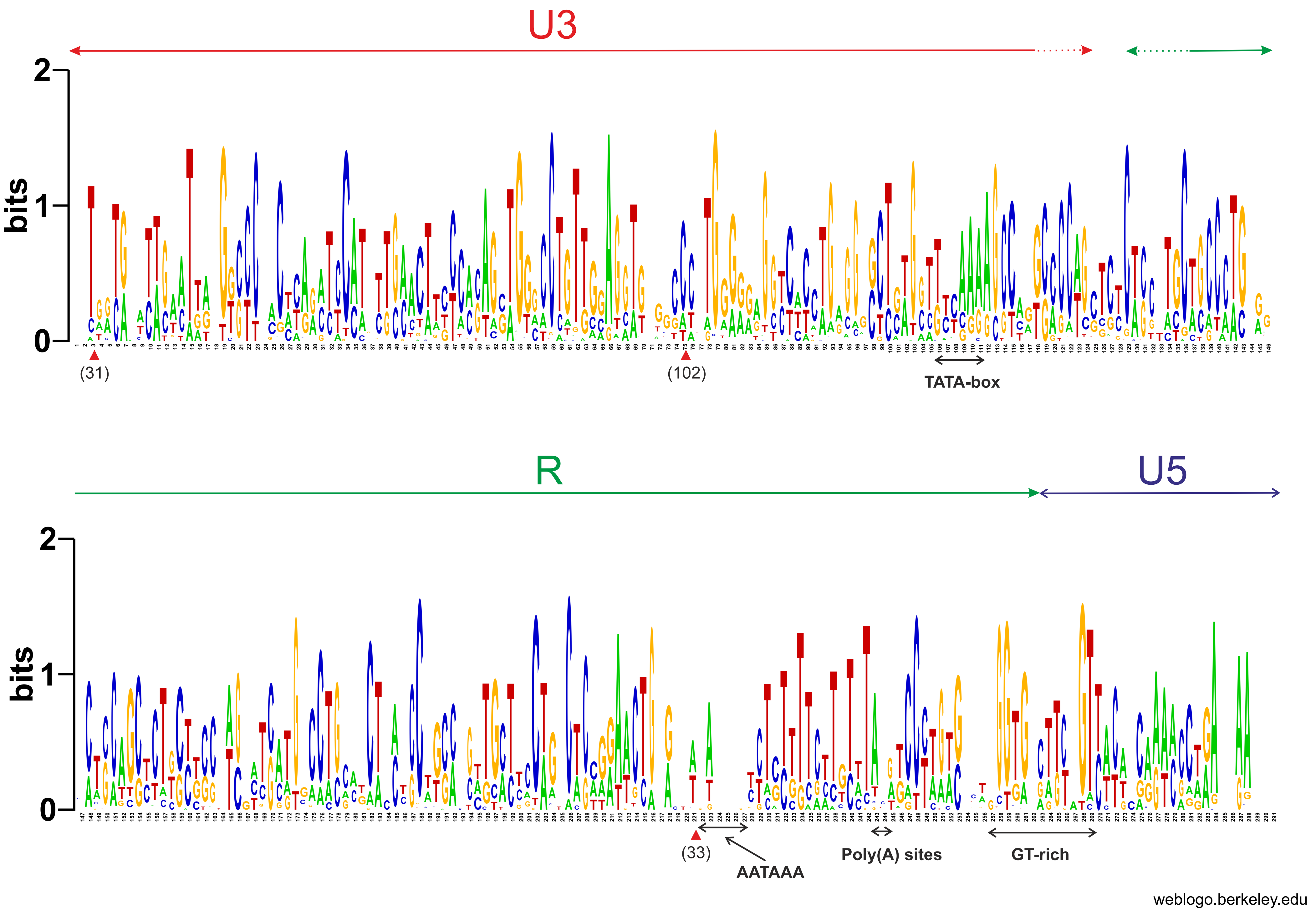

Suppl fig 3, long ERV class III HMM

Supplemental fig 4

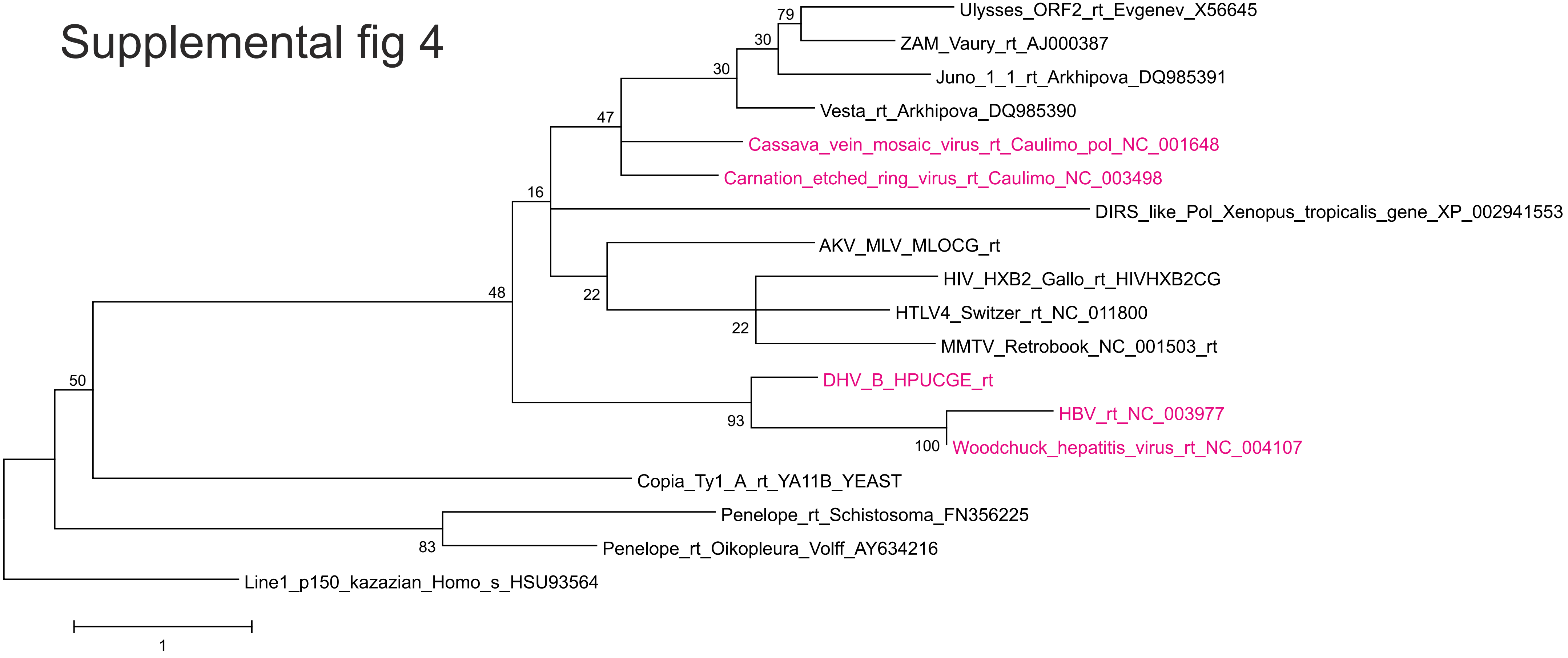

Supplemental material for Benachenhou et al; Conserved structure and reconstructed evolutionary history of Long Terminal Repeats (LTRs).

Aligned training sets,

for the HMMs ERV class III_150, HydraAG_90, Retrofit_150, Sire_150, Sushi_150 and Zam_150

Match States are shown in upper case. Insert states are shown in lower case. “-“ corresponds either to a gap or to an insert state from another sequence with more insert states. Each set is followed by the respective HMM consensus sequence. The alignments are in FASTA format.

…………………………

ERV class III_150

```
>LTR18A
TGTaaggaaaatggmtgcgctktagtcaggagta--------------------------
------------------------------------------------------------
------------------------------------------------------------
------------------------------------------------------------
------------------------------------------------------------
------------------------------------------------------------
------------------------------------------------------------
------------------------------------------------------------
------------------------GG--C-CgA--Ggc---AG--MC-WTC-CG--Gtn-
----C-----AG-CA-T---GA-CTc-AG------------Cgggtttggagcgcaggcg
cacaaccccgcacattatgtaaccacgccacgtgaggcgcatt-----------------
------------------------------------------------------------
------------------------------------------------------------
-----------------------------------AG-G--TGA-T---C-A--C-----
C-C-A--C-----G-T-G-----------------------AGC-T----CG-TG-----
------C---TT---G---GC--Tc----GG-AG-Ccac----------------T--AT
TG---TC----T-GT---AAA-AGg-Ta--T----AAT--------------Taccctgc
taacgctgtacatacggcttgcgcccaggctcactcgcgcccagagagag----------
------------------------------------------------------------
```


------------------------------AG-TAAA-G-CcA-T-G-TC--GA------
--A-Actgt----CT---AcgaTTc--CT-CG-A-GTG--T--TTT--TCc---------
-----------Ag-C---TACCcgccactcgcccaccgactcccctcggacctcagtttg
ggctagaacctg--

---ACA-
>MLT1A0
TGCtatggactgaatgtttgtgtccccccaaaattcatatgttgaagccctaatccccaa
tgtgatggtattaggaggtggggcctttgggaggtgattaggattagatgaggtcatgag
ggcggggccctcataatgggattagtgcccttataaa-----------------------

------------------------AG--A-G-A---------------CCY-CA--Ga--
----G-----AG-CT-----CC-CT--TG------------Ccccttccgccatgtgagg
acacagtgagaaggcgccgtctacgaacc-------------------------------

-----------------------------------AG-G--GAA-T---G-A--G-----
C-C----------C-T-C--AC---------C--AGaaac-TGA-A----TC-TG-----
------C---CGg--Cg--CC--T-----TG-AT-Cttggactt-----------C--CC

AG---CC----T-CC---AGA-AC--Tg--T----GAG--------------A-------

------------------------------AA-TAAA-T-TtC-T-G-TT--GT-T----
T-A-Agctac---CC---A---GT---CT--ATG-GTAt-T--TTG--TTata--Gcagc
ccg--A-----A--C---AGAC--------------------------------------

---TAAgACA-

>LTR16A

TGTgacggacatgraggtgcgctgcccagatccccttcaagaacggaartyttattncc
cyagctgctgggagwgctgtcggcagacagccctcagctgtcagccccttcaggaattgc
ctcngctgaagagagccgcctcgcccaaggtcacgccccctccccggggcagcccacatc
caatgactgrtcaatrtggaggtataaaggcccggccctctcgccccaactcgggacaac
tctgaagggccatyccagctccagagctccccgtggggtc--------------------

------------------------GG--C-T----G-----AG--GC-CTT-TGttG---
----C-----GA-CT-G----C-AT--TG------------C------------------


```
-----------------------------------AG-C--CCA-A---C-T--T-----
C-T-C--C-----C-T-C--TG---------C----------CC-Aat--CC-TG-----
------C---TT-------CC--T-----TC-CC-Tt------------------C--CC
TT---CC----A-CA---GGC-GT--Tga-Tccc-AAGggcgctccc-----T-------
------------------------------------------------------------
------------------------------------------------------------
------------------------------------------------------------
------------------------------------------------------------
------------------------------------------------------------
------------------------------------------------------------
------------------------------AA-TAAA-C-C-T-C-C-TG--CA-C--GC
T-A-Atct-----CC---A----T---CT-CAG-a--G--T--CTG--CTtccc-Gggg-
-----A-----Ac-Cc--AACC--------------------------------------
------------------------------------------------------------
------------------------------------------------------------
------------------------------------------------------------
------------------------------------------------------------
---------------------------------------------------TGCaACA-
>ORR1B
TGTggtggtttgaatraaaatggcccccataggctyatagggagtggcactattaggagg
tgtggccttgttggaggaagtgtgtcactggggtgggctttgaggtytcakatgctcaag
ccaggcccagtgtgttcactctcttcctgctgcctgcagatcaagatgt-----------
------------------------------------------------------------
------------------------------------------------------------
------------------------------------------------------------
------------------------------------------------------------
------------------------------------------------------------
------------------------AG--A---A-------------C-TCT-CA--G---
----C-----TA-CT-T---CT-CC--AG------------C------------------
```

-----------------------------------A--C--CAT-Gt--C-T--G-----

C-C-T--G-----C-A----TG---------C---------TGC------CA-TG-----

------C---TT---C---CCg-Cca---TG-AT-Gataa---------------TggAC

TAaa-CC----T-CT---GAA-AC--Tg--T----AAGccagcccc------A-------

------------------------------AT-TAAA-T-G-T-T-T-TC--CT-T--TA

T-A-Agagttg--CC---T---TG---GT-CATG-GTG--T--CTC--TTcaca-Gcaat

aga--A-----Ac-Cc--TAAC--------------------------------------

---TAAgACA-

>LTR53

TGTggtgtaaggcctaaaattaaggcccaatattatgtgctgccttgacatctggtgaaa

tcaggagggcctcaaatggcctaactacaagttcccctccccactctgctcccatggata

aggtcccctagccaaacaaccctccttatcaaggggaccaggcacagttcctgcttatcc

ctgntgagtagygggtttcagttccctgccagcccgtggaattattcaaayaagccaatc

acatcctcctgcgggaaccaggggtcacctcaccctcttgatactacaa-----------

------------------------AG--C-C----------TG--CC-TCC-CA------

----C-----AG-C------CC-CT--GG------------T------------------

-----------------------------------TG-T--TCA-Ct--C-T--Gtt---

C-C-T--G-------A-G--TG---------C--A------ACC-C----CCaTG-----

----------TGg--C---CC--Tg----TG-TG-Gnatgtggtgtcc-------T--CC

TC---CC----C-CA---GG---C--Tg--T----GAGtatatgtgat----T-------

------------------------------AA-TAAA-C-TgC-T-G-TC--AA-T--CT

C-A-Tctgt----CC---A---GT---GT-TGGGtGTCa-T--GTG--TTtar--Cc---

-----Atc---Cc-Ca--TAACcctagggtgggaatccctccctcaccaatggggtgaag

aggaggcaat--

---WAAaACA-

>MSTD

TGCtatggtttgaatgtgtcccccaaagttcatgtgttggaaacttaatccccaatgcaa

cagtgttgggaggtggggcctaataagaggtgattaggtcatgagggctctgccctcatg

aatggattaatgtcattatcgcgggagtgggttagttatcgcgggagtgggtttgttata

aaagnaagttcggcccccttttgctctctcnctctc------------------------

------------------------AC--T-C-T--C-----TT--GC-CCT-TC------
----C-----GC-CT-T---CCgCC--A--------------------------------

-----------------------------------TG-G--GAT-GacgC-A--G-----
C-A-A--G-----A-A-G---Gcc-------C--TC-----ACC-A----GA-TG-----
------C---CGg--Cn--CC--W-----TG-AT-Cttggactt-----------C--CC
AG---CC----T-CC---AGA-AC--Cg--T----GAGcc------------A-------

------------------------------AA-TAAA-T-TtC-T-G-TT--CT-T--TA
T-A-Aattac---CC---A---GT---CT--GTG-GTAt-T--CTG--TTata--Gcagc
aca--A-----Aa-C---GGAC--------------------------------------

---TAAgACA-
>MLT1M
TGTactagacatgtcaatcacggtgcctgccacgcccacagccccttctaagggaaactg

ccccgcccacagcccctgctgaaggggcagccctaggcggccatgttttgtaccacgtga

ccctgccctcccctggccacagctgattggaccaggggtgggcacctgacccaagggcag

ccaatccataggctggccagcgacctatgacgtggcctggcgcgaaaagatgagctgggc

caatcagattctctctctcgggaatttgaactgggaaacacggagagaatgaggcagtta

gcagcgggagctgaagctgaaaggatgcatagagagaagccatgaggtagagtcggggcc

atgatgggccatgtgcaagccgaagttatgaggaagcagaaactatgagtaagcagagga

agccggtcggtagagagaagagaacggagcagacgngcagagagaagccgagacgcgtga

tgagagagggaccggacgagagc-NG--C-N----G-----AG--GT-CCC-TA--G---

----------AG-CT-G---CC-CC--GG-------------------------------

-----------------------------------TT----CCG-G---C-C--G-----

C-T-T--C-----C-A-G--TC---------C---C-----TGT-T----CC-TGg----

------CgntTT---C---CC--Agt---TC-CA-Gt------------------T--CC

GGt--CC----AcGT---GTA-TC--Ct--T----A----------------C-------

------------------------------AA-TAAA-C-CcC-C-C-TT--TT-C--T-

T-G-Agntaa---CT---T---GA---GT-GA---GTC--T--CTG--TTcctt-Gcaac

caaa-Ag----Ag-Cc--TAAC--------------------------------------

---TAAaACA-

>MLT1E1A

TGGgcagaattctaagatggcccccaagattcccacctcctggtrttcatgccttgtata

atcccctccccttgagtgtgggtaggacctgtgaatatgatggatatcactcttgtgact

atgttatagtatatggcatgaagggattttgcagatgtaattaattttgcagatgtaata

ggtccctaatcagttgactttgagttaatcaaaagggagattatcctgggtgggcctgac

ctaatcaggtgagcccttaaaaaagncntctaaatccgtctngaaagagaagaacta---

------------------------AG--A-G-A--G-----AT--TC-TCC-T-------

-----------G-CT-G---GC-CT--TG------------Aagaagtaagctgccatgt

tgtgangagggtctgtgaagagggccacgtggcaaggacctgagggtggcctctaggagc

tgagagcaatccctggccagccaacagccagcaagaaaatagggacctcagtcmtacagc

tgcaaggaamtgaattctgccaacaacctgaatgagcttggaagaggattctaagcctca

gatgagaacacagccctagccaacaccttgatttcAG-C--CTT-G-----T--Ga----

G-A-C--C-----C-T-G--AG---------C--AG-----AGG-Ac---CC-AG-----

------C---TA---A---GC--Tg----TG-CC-Cagac---------------T--CC

TGac-CC----A-CA---GAA-AC--Tg--T----GAGa-------------T-------

------------------------------AA-TAAA-T-GtG-T-G-TT--GT-T--T-

T-A-Agcca----CT---Aa--GT---TT--GTG-GTAa-T--TTG--TTat---Gcagc

-----A-----At-Aga-AAAC--------------------------------------

---TAAtACA-
>LTR16E2
TGTatcggacacctcttgtgcaccacttcanatcctctcggccccacctcttactccagc
cactgctgcggcgaccagttccgtgcaggcttcgaccagcttcgcgcaggtgcaacctga
cagcgcctcgcctcaggcccgcgccgcgtacctctcgcttcctgccccggggcttctctg
acgccgcggcgtgggacacctgcgggaacccgctcggcactcacgcatgcgcaacccgga
agtgcgggggagttaacgccccgtggggcnacccttgaccaatgggggacgggagccgat
ggataaatgcttcccccttttcgtcccctgggcggacagttctgagacgcatttcataagg
ctcctcagaaggtcccggcgggatcg----------------------------------

------------------------AGcaC-C-A--G-----TC--GC-CCA-CA--G---
----C------G-GT-G---GC-CA--A-------------Ctcgataacgcatccttgt
attggct---

-----------------------------------TT-C--CCT-C---C-T--T-----
C-C----------C-T-G--TTt--------C--AC-----TCC-C----CC-TG-----
----------T----C---CC--Tcac--TC-CT-G-----------------------C
TC---CC----T-G----GGA-TCa-Ct--Tccca-----------------A-------

------------------------------AA-TAAA-C-T-A-C-C-TG--CA-Cg---

C-A-Ag-------CC---Ttt-GT---CT-CAGG----c-T--CTG--CTttc--Ggggg

-----A-----Ac-Cc--AGGC--------------------------------------

---TAAgACA-

>THE1A

TGAtatggtttggctgtgtccccacccaaatctcaacttgaattgtatctcccagaattc

ccacgtgttgtgggagggacccagggggaggtaattgaatcatgggggccggtctttccc

gtgctattctcgtgatagtgaataagtctcacgagatctgatgggtttatcaggggtttc

cgctttt---

-------------------------G--C-T-T--C-----TT--CC-TCA-TT--T---

----------TC-CT--------CT--TG------------C------------------

-----------------------------------CG----CCG-C---CaT--G-----

T-A-A--G-----A-A-G--TG---------C--CT-----TTC-G----CC-TC-----

------C---CG---C---CA--Tga---TT-CT-Gaggcctc------------C--CC

AG---CCa---T-GT---GGA-AC--Tg--T----AAGtcc-----------A-------

------------------------------AT-TAAA-C---C-T-C-TT--TT-T--CT
Tc-----------CC---A---GT---CT-CG-G-GTA--T--GTC--TTtatcaGcagc
gtga-A-----Aa-C---GGAC--------------------------------------

---TAAtACA-
>GGLTR3F1_LTR
TGTcatggttttgtaacttcgctattggtattccacatcataacatcatggacaaaagag
aagaactacgtatcccagaggacctcacggtcagagaaggaagacacatcacgga-----

------------------------AG--A-T-A------------CG-TCA-T-------
----C-----TG-GT-T---GC-GC---G------------Cggtgcttcttcactt---

-----------------------------------TC-G--CTT-G---C-T--G-----
C-C-G--G-----G-A-G--AG---------G--AG-----TGGgT----G--TG-----
------C---TT---T---CC--A-----AG-CC-Ggg-----------------Cg-CC
TT---CA----T-CA---AG----------T----AAGgc------------Tttcggtt
tcggaaactctctcactctctctctctctccctctctatcgctctttcgctctctctccc

tcattccatttggtttattatacttactcccaattagattgtattgtatcgtgtcatctt

gcatcccaacatcatagttagtaa------------------------------------

------------------------------AA-TAAG-----T-T-C-TC--CT-T--CT

T-AgAttgttg--CC---Acc-GC---TT-C----GTT--T--TTC--TCg----Gg---

-----A-----A--G---TGA-aggggagggggcccgcaagcctaccggcccccctgtc

acgggcacagatctatctaggtaactccgtg-----------------------------

---ACA-

>MLT2F

TGTggtggctttgtaatgtgtcaacttggctaggctggaactacatttcccagaattccc

ttccctgtatrkttccaggttagggtgggccacaagagacattctgtgtgagatttggaa

ggcggaagtgaagcagcagccattttgttttctgtgctcggagaggtcagagtcagcagg

cgctgttgcagctcacacacgttgtcgctkatctgctggctcacctcgttggcgtggggc

agc---

------------------------AG--C-C-----------G--GG-CCC-----G---

----C-----AG-CT-G---CT-CC--AS------------Cttcccctggatcctcctt

cagcttctccgactcctgggccaggtgtgtgtktagctccgtgacgaagggcgccagctt

ctcctgcaggacacccacatcatcgaggtcggaggcagtgagagactgacatgggttcca

gtttgtcctcgtgggttccagctcatgcttg-----------------------------

------------------------------------TG-G--GTT-C---C-A--G-----

C-T-T--Gt----C-C-T--TG---------C--TC-----TCC-C----CC-ACtttat

atccatC---TT---C---CC--T-----TC-CT-Gac-----------------Tg-CC

TGc--CC----T-GT---GGA--C--Tt--C----AAGc-------------Tccagcat

cagacgcaragacaacagccttacagagactgcttaaccagctcccacaattgcgtaagg

tcaaatccctat--

------------------------------AA-TAAA-T---C-T-C-TT--AT-Ta-TA

T-A-Tatctc---CT---A---GTg--GT-TCTG-CTTc-T--CTGa-TT-----Ga---

-----A-----C--Cc--TGAC--------------------------------------

---TGAtACA-

>MT2C

TGTagtggctattcctggttgtcaacttgactatatttggaatgaactacaatccagaat

tggaaggctcaccagtgaccctaatctggaggctgggagatacaagtttctgacctggat

cttggtatggagatcttgaggcatagtggctatggattccagaagattaaggcagggaga

tctttg--

------------------------AG--T-T-C--A-----AG---G-TCA-TC--G---

----C--------CT-G------CT--TG------------Cttcgtgagactgagtaac

tgct--

-----------------------------------AG-----AT-C---C-TtgGa----

C-T-T--C-----C-A-T--T----------C--AC-----AGC-T----RC-NA-----

------C---TGaa-C---CA--T-----TG-TT-Ggg-----------------A--AT

TGg--AC----T-GC---AGA--C--Tg--T----AAGtcat----------C-------

------------------------------AA-TAAA-T-T-C-C-T-TT---A-C--TA

T-AgAgagacattCC---AtaaGT----T-C--T-GTG--A--CTC--TAg----Ag---

-----A-----Ac-Cc--TGAC--------------------------------------

---TAAtACA-

>ORR1C

TGTggtggtttgaataagaatggcccccataggctcatatatttgaatgcttggtcacca

gggagtggcactgtttramaggattagaaggattaggaggtgtgrccttgttggaggaag

cgtgtcactggggtgggctttgaggtttcaaaagcccaygcca-----------------

------------------------GG--C-CcA--G-----TG--TN-TCT-CT--Ct--

----Ct----TY-CT-Gnt-GC-CT----------------Ctggatcagtatgcagctc

tcagcta---

-----------------------------------TT----TCT-C---C-A--Gcacta

N-R-T--C-----C-A-C--TGn--------C--A------TGC-Cg---CCaTG-----

------C---TC---Y---CC--A-----TC-AT-Gatgytaa------------TggAC

TAa--CC----T-CT---GAA-AC--Tg--T----AAGcaagccccc-----A-------

------------------------------AT-TAAA-T---G-T-T-TT--CT-Tt-TA

T-A-Agaattg--CC---T---TG---GT-CATG-GTG--T--CTC--TTcaca-Gcaat

ag---A-----Ac-Ag--TGAC--------------------------------------

---TAAgACA-

>LTR18B

TGTaaggtacatggatgtgctttggtcaaggaataggccgaggcggacatccaggcctgc

atgactcagcgagtttggngcgcaggcgcacacctccacttgttatataacctgtttgtg

taagttcatacttggctctgagccactattgtctgtaaaaggtataactgccctgctgac

gctgtgcaggggctcttggggctcagctcggctcaacatggcttgacatggtgggcgcgc

tggcgcccagagaaagagagagagagccagagctgtccgtcttgcagacggacagggggg

agcca---

------------------------GG--A-C-A--C-----AG------CT-TG--G---
----Ct----TG-CT-Cgt-GC-CC--AG------------Agagagaaagagttaagct
gctgaccctgaaggcaagggagagccggccgcgcagctgtgcgtgggggcggcaggagcc
gcagagc---

-----------------------------------TG-G----A-N---C-A--G-----
G-C-A--G-----C-C-G--AGa--------C--AG-----AGG-Cgg--AC-AG-----
----------TG-----------------TG-AGaG------------------------
AG----C----T-GC---TGA-AG--Tg--T----GAG--------------Aagctgct
gatgagagagctgctg--

------------------------------AA-TAAAaC-CaTaT-T-TCacCT-G--CC
T-A-Cggcccc--CCgagT---GTtctTT-CA-G-CTA--T--CTG--CCcat--Ccacc
c----Actc--Cc-Ctc-GGACctcagcatgggctggaacctgaccccgggcctg-----

---ACA-
>MLT1G3
TGMcactcctccatcaagkggtagagtctatttccyctccccttgaatntgggytggnct
ttntgattgcctngaccaatagaatgcagtagaagtgatactgtctgacttcnaaggcta
wggtcataaaaaggccatgcacttctaccttgctctcttgggangcttgctcttggaacc

cagccaccatgctgtgaggaagcccaagccacatggagaggccacatgtaggtgttctgg
ccgacagtcccagctgaggtcccagccaacagccagcatcaaccaccagacatgtgantg
agtgaagaagcctccagatgattccagcccccagctgtcaagtcatccccagcctctnnc
agycmtcccc--

------------------------AG--C-C-TtcA-----AG--TC-TTC-C-------
----C-----AG-CT-GaggCC-CC--AG------------Acatca-------------

-----------------------------------TG-Ga-GCA-GagaC-Aa-G-----
C-CaT--C-----C-C-C--A----------C--TG-----TCC------MY-TG-----
----------TG---C---CC--T-----GT-CT-Gaat----------------T--CC
TGac-CC----A-CA---GAA-TC--Ca--T----GAGca------------T-------

------------------------------AA-TAAA-Tg----G-TT--GT-T----
T-A-Agcca----CT---Aa--GT---TT-TGGG-GTGg-T--TTG--TT----------
-----------A--C---

---ACA-
>LTR33B

TGTgccggattgtctgttgncaactcagcaccatccccgctcccttctangctctcccct

gtatcgcaggggctggaagcctggaaactacatttcccagantcccttgccagcagggtt

ccggnttagattctgccaatgagaggcactcgcgcgagatttggaaggcggaagagaagg

agaagccattattctccngcggcagcngcgggcagacgcgtgggcttcggcagacggcag

atgtgaggttttgccagcggcttccgggcatcctcctgngaatcacccgcttcggtgctg

caggcagctgagatcatcggcggc------------------------------------

------------------------GG--C-T-T---------------CCC-TG--C---

----Gat---TC-CT-G---CA-CT--TC------------C------------------

-----------------------------------TG-A--TTT-C---C-T--G-----

A-A-A--G-----C-T----AG---------C--AGcggt-TTC------CC-TGac---

------C---TT---C---GC--T-----CC-CC-Cag-----------------C--CC

TT---CC----A-AC---GGT-TN--Tg--T----AAGcctctaattccctgT-------

------------------------------AT-TAAA-T-C-C-C-T-TC--CTgC--TT

G-A-Aatac----CT---A---GA---GT----G-GTT--T--CTG--TTttcctGacca

-----A-----Ac-Cc--TGAC--------------------------------------

---TGAtACA-
>ERV3-1N1-LTR_XT
TGTagcacggtggattttattttgattttataatgatttacaatatatacatctgctttg
acatatgaatcaaatcttaggtttgagtcaagtaaaagcagcagattttgggacagctat
tgtccagtgttcaaggtacaatgtctttgttctatgtatattgctgggtaaacagtaaac
acaggtattgttacccagcaaatatccttggaataaacaaacagacagcatatagacaat
acactttatgtacaacttatttgagcaacaaataaggccttctacaaagttagtaaattg
tctagttaataggttttgttcatgacaaaagtttgtacacaatattgcacaagtcagcac
ttggtgttgggggttttgctaagaactctagtataaaaggg-------------------

------------------------AG--C-C-T--CcaccaTG--TG-TC---A--G---
----T-----AG-CT-Tc--GC-CT--AGga----------Ctcctgaacgagtgccggg
acatttggatcatcgcttggttatcgggaacctgaaggtcttgcgccaaaggttg-----

-----------------------------------TG-G--G---G---C-T--C-----
C-C-C--G-----A-TtG--TG---------C--TGa----AT--T----GA-TG-----
------C---AG---A---AC--T-----GG-CTa--------------------Ta-CT
TGta-AC----T--A---TAA-TCa-Tac-T----AAGa-------------T-------

------------------------------AAgTAAA-T---C-TaT-TT--AA-T--TC
T-A-Ttatta---CT---T---GT---GT-GTGT-GTA--A--GTTa-TTg----Ct---
-----C-----A--C---TAACagtctatcagaaggtttaatcattgatcctgcatattt

attaatatttgtcattaatgtcaattaatacaaattattaatattaataaaggttcaccc

cctttatt--

---ACA-

>MLT1C

TGTtatgggttgaattgtgtccccccaaaattcatatgttgaagtcctaacccccagtac

ctcagaatgtgaccttatttggaaatagggtcgttgcagatgtaattagttaagatgagg

tcatactggagtagggtgggcccctaatccaatatgactggtgtccttataaaaagggga

aatttggacacagacacgcacacagggagaacgccatgtgaagatgaaggcagagattgg

ggtgatgcntctaca---

------------------------AG--C-C-A--A-----GGaaCG-CCA-A-------

----------AG-AT-T---GC-C---AG------------Caaaccaccagaagctagg

ggagaggcatggaacagattc---------------------------------------

-----------------------------------T--C--CCT-Ca--C-A--G-----

C-C-C--T-----C-A-Ga-AG---------G--A------ACC-Aac--CC-TG-----

------C---CGa--Ca--CC--T-----TG-AT-Ctcggact------------T--CT

AG---CC----T-CC---AGA-AC--Tg--T----GAGa-------------C-------

------------------------------AA-TAAA-T-TtC-T-G-TT--GT-T----
T-A-Agccac---CC---A---GT---TT--GTG-GTAc-T--TTG--TTac---Ggc--
-----AgccctAg-N---AAAC--------------------------------------

---TAAtACA-
>ORR1B2
TGTggtggtttgaatgaaaatggcccccataggctcatagggagtggcactattaggagg
tgtggccttgttggagtaggtgtggccttgttggaggaagtgtgtcacttgggggtgggc
tttgaggtttcagaagctcaagcca-----------------------------------

------------------------GG--C-CcA--G-----TG--TC-TCA-CT--Ctct
cttcC-----TG-CT-G----C-CT---G------------Cagatccagatgtagaact
ctcagctac---

-----------------------------------TT-----CT-C---C-A--G-----
C-A-C--C-----A-T-G--TCtgc------C--TGca---TGC-Ca---CCaTG-----
------C---TT---C---CC--Acca--TG-AT-Gataa---------------TggAC
TAaa-CC----T-CT---GAA-AC--Tg--T----AAGccagcccc------A-------

------------------------------AT-TAAA-T-G-T-T-T-TC--CT-T--TA
T-A-Agagtt---GC---T---GTg--GT-CATG-GTG--T--CTC--TTcaca-Gcaat
aga--A-----Ac-Cc--TAAC--------------------------------------

---TAAg----

>LTR16E1

TGTagcagatgcctctggtgccccgcgtcacatcccctcggcccacctctgatttcagcc
gcagctgcggtggacagttccgtgcgagctcagactcacctttgctgacagcatcccacc
tcaagcgcgcgccgtgcgtctttctgctttctgccccagggccttctccgacgccgcggg
agcccgctcggcccgcgcgcaagcacagcccggaagtgcggggg----------------

------------------------AG--T-T-A--A-----TG---C-CCC-CG--G---
----G-----GG-CA-A---CC-CT----------------Caaccaatgggggacggga
gccagtggataaatgctccagcctcccgtccttcaggtggacaattctgggaggcattct
gtacgcttctcaggaggtcccagcggaatcgagcccccgttgcccacagcagcgacctcg
ataacgcacccttatattggctt-------------------------------------
-----------------------------------TT----CCT-C---C-T--T-----
C-C----------C-T-G--TCt--------C--AC-----TTC-C----CC--G-----
------C---T--------CC--C-----TC-AC---------------------T--CC
TG---CT----T-CCtg-GGA-TCa-Cc--Tccc------------------A-------

```
------------------------------------------------------------
------------------------------------------------------------
------------------------------------------------------------
------------------------------------------------------------
------------------------------------------------------------
------------------------------------------------------------
------------------------------AA-TAAA-C-T-A-C-C-TG--CA-C--CC
--A-Agtc-----CT---T---GT---CT-CAGG----c-T--CTG--CTttc--Ggggg
-----A-----Ac-Cc--AAAC--------------------------------------
------------------------------------------------------------
------------------------------------------------------------
------------------------------------------------------------
------------------------------------------------------------
---------------------------------------------------TAAgACAg
>MLT1G1
TGTggcagattgtattttccaaagatggccgcaacaatatctcccatcccacatgctctt
cttacaatgtgaccttgncactcctcccatcgagnggtggggtctatgtcccctcccctt
gaacctgggcggacctttgtgactgccttgaccaatagagtatggcagaagtgatgctgt
gtgacttccgaggctaggtcataaaaatgccatgcacttccgccttgctctcttgggacg
ctcgctcttggaacccagccaccatgctgtgaggaagcccaagcagcccatggagaggcc
cacatggagagg------------------------------------------------
------------------------------------------------------------
------------------------------------------------------------
------------------------AA--C-C----G-----AG--GC-TCC-CG--G---
----Cc----AA-CA-G---CC-CC--AG------------Ctgagntcccagccgacag
ccagcatcaactgccagncatgtgagtgagccagcctnnagatgactccagcccccagcc
attgagtcacccccanccgtcgagcc----------------------------------
------------------------------------------------------------
-----------------------------------AT-C--CCA-G---C-T--Ga----
```

CgC-C--G-----CgT-Gg-AG---------C--AG-----AGA-C----G--AG-----
------C---CG---T---CC--C-----CG-CC-Gag-----------------C--CC
TGc--CCaaatT-GC---AGA-TT--Cg--T----GAGca------------A-------

------------------------------AA-TAAA-T-GaT-T-G-TT--GT-TgtTT
T-A-Agcca----CT---Aa--GT---TT-TGGG-GTGg-T--TTG--TTac---Gcagc
-----A-----At-Aga-TAAC--------------------------------------

---TGGaACA-
>MLT1F
TGTggtagccagcctccaagatggcccccaatgatccctgcctcctggtattcacgccct
tgtgtagtcccctcccacattgaatagggctgacctgtgtgaccaatagaatatngcgga
aatgatggtgtgtgacttccgaggctaggtcataaaagacattgcggcttctgccttgct
ctctttggatcactcgctctggggggaagccagctgccatgttatgaggacactcaagcag
ccctgtggagaggtccacgtggcgaggaactgaggcctcctgccaacagccagcaccaac
ttgccagccatgtgagtgagccatcttggaagcggatcctccagcccc------------

------------------------AG--T-C-A--------AG---C-CTT-CA--Gatg
a---C-----TG-CA-G---CC-CC--GG------------Ccgacatcttgac------

-----------------------------------TG-CaaCCT-C---A-T--Gaga--

G-A-C--C-----C-T-G--AGc--------C--AGa----ACC-Ac---CC-AG-----

------C---TA---A---GC--C------G-CT-Cccaaat-------------T--CC

TGac-CC----A-CA---GAA-AC--Tg--T----GAGa-------------T-------

------------------------------AA-TAAA-T-G-T-T-TaTT--GT-T--T-

T-A-Agcca----CT---Aa--GT---TT-TGGG-GTAa-T--TTG--TTac---Gcagc

-----A-----At-Aga-TAAC--------------------------------------

---TAAtACA-

>LTR41B

TGTgccagttatcaatttattgcctctcagctccaaatttacctttcaatacctgctctg

tgataatggactgaaytctttaagcatttctcctttacagtgagcatgatgttaagcttt

ctcagtagagggtgctggagggacattgcaggargaaggggggcttctcttcctggttcct

gtgtgctgcatttggcttttttcttgctccagtgtcagggtctgtcagcagtgtgtgtgtg

tggggacatctagtggtgctctgccccagctgtgccccag--------------------

------------------------AG--CgC-A--C-----AG--TC-TCT-CG--Gtga

c---Ct----CG-CA-G---CC-CC--GG------------Cctggcctagtgatcacct

tcctgtggccctcccgatatggacactg--------------------------------

-----------------------------------TG-T--GCT-C---C-A--Gg----

C-C-T--C-----C-T-GccAG---------C--AG-----TGC-Cac--CC-TG-----

------A---TT---C---CC--Tc----TGcAC-G----------------------CC

TGt--CC----A-CT---AG---C--Ct--T----G-Gc-------------Tcgcctgc

actccagagggttgtttctgtggccctcccaacgcggatagcatgtgctccagrcctcgc

aaaccagcagcagtgattctctctgcgtgyccgcctaccagcctcggcttacctgtaccc

cagagggttgtttcctgcttgtctagcgactgtagaccagctctggcctgggcaacccag

caaacttctccgccatccagtgggctgcaaccacaccttctccaacgaggtctgaacccc

agccttggggagggagccctccttccaagtttgtccttctttgggtattctctctttcct

ttggtattctccatcagccctagagtactctttagagttctctttacatctttatagtta

ctcccctatcatagtttaataattctttatAT-TAAA-----C-T-T-TC--CC-Tg-TT

T-A-Aatt-----AC---T---GT---GT----G-GTT--T--CTG--TCtcct-Gattg

g----A-----C--Cc--AGAC--------------------------------------

---TGAtACA-

>LTR40A

TGTtgggagacaattctccatgggtctctcgcatttctgcacgtcttgtgagcagaggca

ctgactgcctttgttctggactatcttttcaaggatgtttgtatagcgaacagccttgga

agatag--

------------------------AG--A-T-A--G-----TG--TC-TCC-CT------

-------------CT-G---G------AG------------Caaagggcaggtttgctta

ctagccttgnaarataaagataatgtctccctccggggcaaagggcaggcatgcttactg

cccattataaaagattngggtttcctaagctcggggttcctcwsctgtracgcaaaccca

ctgcgtgtgcagnantcatc--

-----------------------------------TG-G--SCC-C---C-TtcG-----

C-A-T--Cgcc--C-T-Cg-TGgga------Ct-TGg----GGG-N----CA-AGgggaa

------C---TGa--C---GC--Aaaca-TG-AT-Gctca---------------T--GC

TG---CC----T-GC---TGT-GC--Tg--T----GAR--------------T-------

------------------------------AA-TAAA-G-T-C-C-T-TT--GT-C--TC

T-G-Ac-------CC---AggaGT---CT-CGT--GTCt-T--CTG--CCa----Gc---

-----At----C--Ca--TGA-aactgtggcaggctaacttgttagcttgcaagtagggt

aaaatctcagacccttcacagttcttg---------------------------------

---ACA-

>MSTA1

TGCtatagtttggatatttgtcccctccaaatctcatgttgaaatttgatccccaatttg

gcartgttggaggtggggcctagtgggaggtgtttgggtcatgggggcagatccctcatg

aatagattaatgccctcctttgnggtgggaatgagtgagttctcactctattgtgggaat

ctattagttcccataagagctggttgttaaaaagagcctggcacctncctcctctctctc

tcttgcttgcttcctctctcaccatgtgatctctgcacacgct-----------------

------------------------GG--C-T-C--Ccc---TT--CC-CCT-T-------

----C-----AC-CT-Tcy-GC-CA--TG-------------------------------

-----------------------------------AGtG--GAA-G---C-A--G-----

C-C-T--GaggccC-T-C--AC---------C--AGa----TGC-A----GA-TG-----

------C---TCg--Ca--CC--A-----TG-CT-Ttttg---------------T--CC

AG---CC----A-GC---AGA-AY--Ta--T----GAGcc------------A-------

------------------------------AA-TAAA-C-CtC-T-T-TT--CT-T--TA

T-A-Aattac---CC---A---GC---CT-CA-G-GTA--T--TCC--TTtata-Gcaac

ac---A-----Aa-At--GGAC--------------------------------------

---TAAgACA-

>MER54B

TGTagtgaattcttataattttatgttgcctcggcatccattttgaatacaggtttaact
ttctcataccagaagcagggctcagtcacccttgacacagtttccagttctacaccacac
ccaaatggctcaagccggtggccagagataagaacttagaggcatctctcccgcctagca
gactgggctccccgctttcccgccgcttcctttaaanggaccattcaggcatttgcccgc
gaacttaaagtgacccacaccctattcccttatatatactgctagtt-------------

-------------------------G--C-C-A--------TG--TC-CTC-T-------
----C-----T--CT-G----C-CT---Gact---------C------------------

-----------------------------------TT-C--ATT-C---C-T--G-----
C-C-T--C-----G-C-G--TGacccggggaC--GG-----AGG-A-----C-TG-----
------C---CCt--C---CCgaC-----TC-AT-Tgcg----------------C--CC
TC---CC----T-GCccaGGA-TC--Tg--T----AAG--------------T-------

------------------------------AA--AAA-T---C-T-T-TG--AA-C--T-
T-G-Tttc-----CT---A---TT---GT-GGTG-GTG--Ta-TTGaaTTt----Gcgcc
ttcc-At----CtgAa--GAACcaggggctgccccaggccgggttttccccgggacgccg
gggagaacacaaggtcgggctcccagcgccagagcgatggtcaggcaggcataaactgga
cacgggtcagacaagagccacaagggcatctgccagtataaacaagtttcccgtgtgagg

gaccccctggtcacgggtcggacaactaggcattaggccgtccgccaggtaaaagaagta
tcccgtgaaaggcacactgtaaacacccacgtccagctccccttcatttcccgttagggc
agggttgctagccgctctggtactggaaccccaatttagctgggggctctc-AAaACA-
>LTR66
TGTggaggaaaagttaaatattaaatttgaactcaattgaacatggacacaaacaatggt
caccaagtcctggaacaggttgtgtgagccccttgaggcattcatccagcgctgtttcgg
agaaatctctatttcaatctattcctatacattagttattgaaaaacaatagacaatcac
aaaaacaagttgacctttttgtgttccttgagcccagtcgtgaagggccctcgtgactgg
gcctcatgccaaacaactcgttacaaaag-------------------------------

------------------------AG--C-T-A---------G--GG-TCC-CA--G---
----------A--CT-G---CR-CCraAG------------Cttcatgagacctctcctc
gtctgtgcacggatgag---

-----------------------------------TG-G--CC-----------G-----
A-C-T--------C-T-Gg-AGcc-------C--AG------GC-T----GT-TG-----
------C---TT---C---CC--Agtc--TG-GT-Gg------------------Tg---
AAt--CC----T-CC---ATAgTC--Tgg-T----GAG--------------Tgtaaata
tatatatatatatatatatacatatatatatatcttttcccttctccccttcccattgca
atttgcttattatatcaatttgcttattatatcatttgcttattatatctgcattgccat
ttacgtgg--

------------------------------GA-TAAA-G-C-T-T-G-TT--TA-Cc-CT
T-A-Aag------GT---A---TT---GT-GTGT-GTG--T--CTT--TTctt--Ct---

```
-----C-----C--Cc--TCACgcgtttcccgcacaga----------------------
------------------------------------------------------------
------------------------------------------------------------
------------------------------------------------------------
------------------------------------------------------------
-------------------------------------------------------ACA-
>ERV3-1-LTR_XT
TGTagtgcagtaggtttcttttactatgatttgtatgatttacaatttatacatgcttta
atatgtgtatgtatatatatatgtatatggtttagttagtaagtttaatcaagcaattct
agagataaggggagtccagtgtccttgtacatatattgttgggtaacaatacctgcgttt
attatgtaacaataaacaatgactatagactgtttacccagcaaatacaacaagcggacg
gcaggcagacaatgcccaatgtatttaaataagaacttgaggtataaagtttggtatctg
atcagacacaatggctgaccatttgttaaacaatattgttcaagtcaatagttgggcttg
ggggtttttcctaagaacttttggcataaaagaac-------------------------
------------------------------------------------------------
------------------------GG--C-C-T--C-----TG-----CCT-TG--G---
----GtcagaAG-CT-Tc--GC-CT--AGga----------Ctcctgaacgagtgccagg
atattggatcatcgcatggttatcgggaacctgaaggttttgcaccaaaggttg------
------------------------------------------------------------
------------------------------------------------------------
-----------------------------------AG-G--G---G---C-T--C-----
C-C-C--G-----AgT-T--TG---------C--TGga---TTA-At---GC-TGaa---
------C---TG-------GC--Ta----TC-TT---------------------T--GT
AA---CC----A-C----AAA-AC--Cg--Tacg-AAGc-------------T-------
------------------------------------------------------------
------------------------------------------------------------
------------------------------------------------------------
------------------------------------------------------------
------------------------------------------------------------
```

------------------------------AAgTAAA-T---G-T-T-TT--AAaT--TC

T-A-Tta------CT---At--GT---GT-GTGT-GTAagTtaTTG--CTc----Attt-

-----A-----At-Ag--T---ttttcatcagaaggtttaatcattgatcctgttaatat

aaataatattgataaaggttaacccctttatt----------------------------

---ACA-

>MER70A

TGCaggacagttctccgggtggccttggaccgacccagttctccccnctttctcgcttgt

agttctcaagaataactgtagaatgtgctgggaatgcaacatcctgagatagggaggaac

tggccggaacagcccgggctctgttccagtccctcctagaaacaggatgtccttcaacgc

tttagcccagcgagtcatgtngcccctgaggtataaaacccagggcgggctgctttccgg

ggtccctcagctgcggtgcaagtggggcacgcgc--------------------------

------------------------AG--N-C----G-----AG--AC-TCC---------

----------AT-CC-G---CC-CTg-GG------------C------------------

-----------------------------------AG-C--TTT-C---C-T--Gag---

C-C-T----------T-G--GGgga------C--CGgc---TCG-C----NA-TG-----

------Aa--TC---CtagGC--Ttc---TG-TT-Gtc-----------------C--CT

TG----C----T-GC---CTA-TC--Tg--T----AAG--------------T-------

------------------------------AA-TAAA-C-CcG-C-T-TC--AT-G----
T-A-Actt-----GT---Tgc-GT---GT-GTGN-GTGt-T--CTG--TCt----C----
-----A-----C--C---GGACtcagacaagttggtaaccagtgcacagtgaacctgctt
c---

---ACA-

>THE1C

TGAtatggtttggctgtgtccccacccaaatctcatcttgaattgtagttcccataatcc
ccacgtgtcgtgggagggacccggtgggaggtaattgaatcatgggggcggttacccca
tgctgttctcgtgatagtgagtgagttctcacgagatctgatggttttataagg------

------------------------GG--C-T-T--------TT--CC-CCC-TT------
----------TG-CTcG---GCaCT--T------------C------------------

-----------------------------------T--C--CTT-G---C-T--G-----
C-C-G--C-----CaT-G--TGaagaaggacG--TGtt---TGC-Ttcc-CC-TT-----
------C---CG-------CC--A-----TG-AT-Tgtaagtttcctgaggcctc C--CC

AG---CCa---T-GC---TGA-AC--Tg--T----GAGtc------------A-------

------------------------------AT-TAAA-C-CtC-T-T-TC--CT-T--TA
T-A-Aattac---CC---A---GT---CT-CG-G-GTA--T--GTC--TTtattaGcagc
gtgagA-----A--C---GGAC--------------------------------------

---TAAtACA-

>MER70C

TGTtgggaaaggcagtctcatgcatgcagtctttcgaccccccactcagccgcataagaat
gggccttgggcctggaacacttccttaccaagagataaagagtcctcacagcctgtgctg
gacttatcaccttgtgtgggaatatctttccctgtttcagactcaatgtgtactcctttg
ttctgcttaagcgtgtgcgtcatatggcacctggccaaccccactgctatatctgtcccc
tgcggggaggggacggggtccttctgctgcagcacaagaggaggtggctgtgtgcctgca

------------------------GG--C-C-A--A-----TT--GC-CCTgCG--T---
----C-----RG-CT-G----C-C---GG------------G------------------

-----------------------------------AG-GgaCCC-G---C-T--G-----

G-C----C-----A-T-G--GGgga------C--CG-----ACG-C----CC-ACta---

------C---TGa--A---GC--Tga---TC-TT-G-------------------C--TC

TG---TC----T-CT-------TC--Tc--T----ATG--------------T-------

------------------------------GAgTAAA-----G-C-G-TT--GT----TC

C-A-Tccagtg--CT---T---GA---CTgCGTT-GTGt-T--TTC--CTt----Ggcg-

-----A-----Ct-CcgaTACCaagatgcagtgggcagaagtgctcggacttctactcct

gataataggcaacagatgccacttgctcg-------------------------------

---ACA-

>ORR1C1

TGTggtggtttgaataagaatggcccccctcccataggcctcatatatttgaatgcttgg

tcatcagggagtggcactacttgagaaggattaggaggtgtggccttgttggaggaagtg

tgtcaacttgggggtgggctttgaggatttttcaaacagcccaagccaggcccagt----

------------------------GG--T-C-T--Ctc---TG--GC-TCT-AG--T---

----Cc----TG-CT-G----C-CT---G------------Cagatccagatgtagagac

tctcagctacttctccagcacca-------------------------------------

-----------------------------------TG----TCTgC---C-T--G-----

C-A-T--G-----C-T-G---C---------C--A------TGC-T-----C-TC-----

------C---TG-------CC--A-----TG-AT-Gataa---------------TggAC

TAaa-CC----T-CT---GAA-AC--Tg--T----AAGccagcccc------A-------

------------------------------AT-TAAA-T-G-C-T-T-TC--TT-T--TA

T-A-Agagttg--CCg--T---G----GT-CATG-GTG--T--CTC--TTcaca-Gc---

-----A-----At-A---GAACactacgtgacttaagtggtt------------------

---ACA-

>MLT2D

TGTgatagttaattttatgtgtcaacttggctaggctatggtgtccagacgtttggtcaa

acattagtctgggtgtttctgtgaaggttatttttggatgagattaacatttaaatcgg

tagactgagtaaagcagattaccctccctaatgtgggtggacctcatctaatcagttgaa

ggcctgaatggaaaaaaa--

------------------------GG--G-C-T--G-----AC--CC-TCC-C-------

---------------------C-CA--AGtaagaggaaattC------------------

-----------------------------------TG--------C---C-T--G-----

C-C-T--G-----A-TnG--TCttcgaa---C--TGgaat-ATC-A----GC-T------

------C---TG---C---GG--A-----TT-TT-Ggact---------------Tg-CC

AG---CC----T-CC---ATA-AT--TgcaT----GAGccaattcctta---T-------

------------------------------AA-TAAA-TcTaT-C-TaCT--CT-C--TC

T-AcAcaca----CC---C---TA---TT-GGTT-CTG--T--TTC--TCt----Ggag-

-----A-----Ac-Cc--TGAC--------------------------------------

---TAAtACA-

>LTR41C

TGTgccagttatcaatttattgcctctcagctccaaatccacctttgtcctgctttgtga

tactggagctggaccctgtaaacatttctcctttgccagctggcacaatgttaagctttg

tcagtagagggcactggagggacactgcaggaggaagggacttcyttcctggttcttgtg

tgctttttttgctcctatggcacatgrttgccagcggcatgtgggacacccagtggcactc

accctccagcaagtttcgccggcacccctgtaaagtcttgttagctccccagagggtggc

ttcccagtgagtttcaccagcattccagaggcctgcttcccagtgagtttcactggcaac

cccagcaggcggtttccagccaccccc---------------------------------

------------------------GG--C-C-T--G-----TG--GCaCCT-CA--G---

----Ca----AA-CT-T----C-TC--TG------------Ccatccastgggccacacc

atctccacataggagtttagatctcagcgttggagagrgcctccttccaagtttgttcct

tcct--

-----------------------------------TG-G--GTA-Ct--C-T--G-----

C-C-T--------C-A-G--CC---------Ct-AG-----AGG-TagtgGC-TG-----

------C---T----C---CC--Ta----T--AT-C-------------------T--GC

TAtt-CC----T-GT---AT--TC--Tt--Ta---GAG--------------Ttctcttt

accccttcttagttaatcctattattaattaattcttagttattataataattctttat-

------------------------------AT-TAAA-----M-T-T-TC--CC-Tg-TT

C-A-Aa-------TT---A---CT---GT-TGTG-GTT--T--CTG--TCtcct-Gactg

g----A-----C--Cc--TGAC--------------------------------------

---TGAtACA-

>MLT1D

TGTggtaggcwgaataatggcccccaaagatgtccacgtcctaatccccggaacctgtg

aatatgttaccttacatggcaaaagggactttgcagatgtgattaagttaaggatcttga
gatggggagattatcctggattatccgggtgggcccaatgtaatcacaagggtccttata
agagggaggcaggagggtcagagtcagagaaggagatgtgacgacggaagcagaggtcgg
agtgacgacgttgctggctttgaagatggaggaaggggccacgagccaaggaatgcgggc
ggcctctagaagctggaaaaggcaaggaaac-----------------------------

------------------------GG--A-T-T--C---------TC-CCC-TA--G---
----------AGcCT------C-C---AG------------A------------------

-----------------------------------AG-G--AAC-G---C-A--Gc----
C-C-T--G-----C-C-G--ACac-------Ct-TGattttAGC------CC-AG-----
----------TGagaC---CC--A-----TT-TC-Ggac----------------T--TC
TGa--CC----T-CC---AGA-AC--Tg--T----AAGa-------------T-------

------------------------------AA-TAAA-T-TtG-T-G-TT--GT-T--T-
T-A-Agcca----CT---Aa--GT---TT--GTG-GTAa-T--TTG--TTaca--Gcagc
-----A-----At-Agg-AAAC--------------------------------------

---TAAtACA-

>LTR16C

TGTagcagacgctgtcggtgtcctggctcatcacccccctcagccctcaacatttcnatgt

acactggcccgacttccaactgccagcacctgcanctctctgcctgagggctttctctgg

ccgcgggagcccgctctgcccgcgcgcagggcaggccggaagtgccggggaattaatgcc

cctnggaagcagccctcaaccaatgacngatgggagttggtgtataaataccccagctcc

ctcgcccctcgggtgggataactctgaggcatgtgttct---------------------

----------------------------A-C-A--C-----TG--TC-TCC-CA--G---

----------AG--T-T---CC-CC--AG------------Cgggattgagctccagttg

cccacagtggtaactngcttgataacgcacccttat------------------------

-----------------------------------TG-G--CTT-C---C-T--Tc----

C-C-TtcC-----C-T-G--TCt--------C--AC-----TTC-C----CC-A------

------C---TC---C---CC--TaccagTG-TT---------------------T--CC

TGggaTC----A-CC-------TC--Cc------------------------A-------

------------------------------AA-TAAA-C-T-A-C-T-TG--CA-C--TC

G-A-Atc------CT---T---GT---CT-CAGG-GTC--Tg-CTT--CTg----Gggg-

-----A-----Ac-Cc--AAAC--------------------------------------

---TAAgACA-
>MLT1H
TGTggtagattgattgcaaaaatggccacaattmtccacccctccctgtatccatgccct
ttgcaatgtgactttgcagctcctcccatcaagaggtggagtctatttccccaccccttg
aatctgggctggccttgtgacttgctttggccaatagaatgtggcagaagtgacggtgtg
ccagttctgagcctaggcctcaagaggccttgcacgcttctgctctctctcttggaaccc
tgccaccgccatgtgaacaagcccgggctagcctgctggaggatgagagaccacgtggag
cag---

------------------------AG--C-C----G-----AG--CCgTCC-CA--G---
----C-----TG--A-G---GC-CA--TC------------Ctagacnggccagccccca
gccaagatcagcagagccgcctactcaaccagcagctgaccacagatgcatgaggg----

-----------------------------------AG-C--CCA-G---C-C--G-----
A-G-A--C-----C-A-G--A-------------AGa----ACC-Ac---CC-AG-----
------C---TGag-C---CC--A------G-CC-Caaat---------------Tg-CC
GA---CC----C-GC---AGA-AT--Cg--T----GAGcta-----------A-------

------------------------------AA-TAAA-T-GgT-T-G-TT--GT-T--T-

T-A-Agcca----CT---Aa--GT---TT-TGGG-GTGg-T--TTG--TTac---Gcagc

a----At----Ag-C---TAAC--------------------------------------

---TGAtACA-

>MLT2B4

TGTgatggttaattttatgtgtcaacttgactgggccatrgggtgcccagatatttggtc

aaacattattctgggtgtgtctgtgagggtgtttctgggatgagattaacatttgaattg

gtagactgagtaaagcagattgccctccccaatgtgggtgggcctcatccaatcmgttga

aggcctgaatagaacaaaaaggctgactctcccctgagtaag------------------

------------------------AG--A-G-A--A-----TT--CC-TCC-TG--C---

----C-----TGaCT-G---CC-TT--TG------------Aactgggacatcggtcttt

ttcctgcctttagactcaaactgaaacatcagctcttcctgggtcttaagcctgctggcc

ttcagactggaactacaccatcggctctcctggttctcaggccttcagacttggactgga

actacaccatc---

-----------------------------------AG-C--TCT-C---C-T--Ggg---

T-C-T--C-----C-A-GctTG---------CcaAC-----TCA-C----CC-TG-----

------C---AG-----------A-----TC-TT-Gggactt-------------C--TC

AG---CC----T-CC---ATA-ATcaTr--T----GAGccaattcctta---T-------

------------------------------AA-TAAA-T-CtC-T-C-TC--TT-N--TA

T-A-Tatanta--CC---Aca-TC---CT-ATTG-GT---T--CTG--TTtctctGgag-

-----A-----Ac-C------C--------------------------------------

---TAAtACA-

>MTC

TGTgctggctagttttatgtcaacttgacacaagctagagtcatytgagargagrgaacc

tcaattgagaaaatgcctccataagattggcctgtaggcaagcctrtaggrcattttctt

aattagtgattgatgggggagggcccagcccattgtgggtggtgccayccctgggctggt

ggtcctgggttctataagaaagcaggctgagcaagccatgaggagcaagccagtaagcag

cactcctccat---

------------------------GG--C-C-T--C-----TG-----CAT-CA--G---

----T-----TC-CT-G----C-CT---C------------C------------------

-----------------------------------AG-G---TT-C---C-T--Gc----

C-C-T--G-----C-T----TG------------AG-----TTC-Ctgt-CC-TGa----

------C---TT---C---CT--T-----CG-AT-Ga------------------TgaAC

TGt--GA----T-GT---GGA-AG--Tg--T----AAGcc------------A-------

------------------------------AA-TAAA-C-C-C-T-T-TC--CT-C--CC
C-A-Agttg----CT---T---TG---GT-CATG-GTG--T--TTC--ATcaca-Gcaaa
tgt--A-----Ac-Cc--TRAC--------------------------------------

---YAAgACA-
>hmmcons
TGT---

-----------------------GGG--C-C-A--G-----TG--TC-CCC-CG--G---
----C-----AG-CT-G---GC-CT--AG------------C------------------

-----------------------------------TG-G--CCT-C---C-T--G-----
C-C-T--C-----C-T-G--TG---------C--AG-----TGC-C----CC-TG-----

------C---TG---C---CC--T-----TG-CT-G-------------------T--CC

TG---CC----T-CC---GGA-AC--T---T----GAG--------------T-------

------------------------------AA-TAAA-T-T-C-T-T-TT--CT-T--TT

T-A-A--------CC---A---GT---GT-CATG-GTG--T--CTG--TT-----G----

-----A-----A--C---TAAC--------------------------------------

---TAA-ACA-

…………………………

HydraAG_90

>GYPSY59_LTR_AG

TGTTGG-G--

---------T--------GCGTA-Cg-T---G--CA-G-Ggtactcacgcgccctctctg

cgagatgttttctctcgtcggttgatgggcacgagtgtcct-------------------

-------------A-GCGAGAGATt----C-G-T--C-ccgatccgcgatcccgattgtt

actcgttacacgatcgggatgcgagcggcagtcgggatacagctagcaacgaatgtggtg

tggtgtcagtaac---------------------A--AG---------------------

----------------------------------GcgGAAATA----AA-------Tt-A

--AG---T-------------------------TTatttattgtagt----T--T-----

--ATc-TAAttt---ACGTT--G--TgtcgtaaacaCT-TA--TT-----CGgccacA--

---------Tatcacacg---------G---CAA-CGTAAAA

>GYPSY58_LTR_AG

TGTTGT-Gagctaacctggcccggactcattcacggcggtaactcacgga----------

---------T--------GACAG-C--A---A---A---Gcgtcatccgtcaatgta---

-------------T-GCGTGTGAT-----C-GaT--C-cgggggtcatcggttgcgcggt

cacggacgttgaccgccaaacaccagcgcgcactgaagaactagcggtcagcaaggaagc

gtgctcgaagccaacaccaaattgtaaccaagtgA--AG---------------------

----------------------------------T--TGAATA----TAtacgttaGtaA

--AT---T-------------------------TA----------------T--T-----

--AG--TAA------CGCGT--G--Agt--------TT-TA--TT-----CG-----A--

---------Ccacctctgcgatcga--A---AGAACATAAAA

>GYPSY28_LTR_AG

TGTTGT-G--

---------T---------ACAA-Cg--g--G--GA-T-Gggaa----------------

-------------T-GTGACACTTca---C-G-G--CC----------------------

----------------------------------A--AGgtatatgtttaccttacagtg

tgggaaccttgggaagaaaagggggttccggaagaagaactgtcagagggagaacaaaaa

gggaagcctacgagctaggaagcaattacgaggcA---GAATA----AAt------Aa-T

--AC---A-------------------------GTgaac------------T--Acg---

--AT--AAAt-----AAC-T--G--T-------------TC--CT-----TA-----A--

---------Ttac--------------A---AAGATACAACA

>GYPSY19_LTR_AG

TGTCAT-A--

---------Tacgtct--GACAG-C--T---GtcCA-TcGacagaaac------------

-------------T-GCTAGT-TG-----G-G-TagCA----------------------

----------------------------------AacAGctgtcagtg------------

----------------------------------A---GAATA----AAcg-----Gc-A

--CTctgT-------------------------GTctgaact---------AcgA-----

--ACg-AAAca----CATGT--G--T-----------C-TTccTT-----CC-----A--

---------C-----------------G---AGT-TATAACA

>GYPSY66_LTR_AG

TGTTAT-G--

---------Tat------GTGAA-C------G--CA-G-Gata-----------------

-------------A-GTGACAGA------C-G-T--CA----------------------

----------------------------------Ac-AGctgtcatccaagcagaccacg

ccgcttgcgtccgcgctttttggacttcttcttttctctccgattgccattgttatcgcg

tataaaacgaattg--------------------T--GTAA-AcccgAA-------G--T

--AT---T-------------------------TTtc--------------T--A-----

--AT--AAA------CCAGT--G--A--------------A--CT-----CA-----Caa

cag------T-----------------A---AATACAGAACA

>GYPSY57_LTR_AG

TGTTAT-G--

---------G--------GCCAA-Cc-T---G---A-T-Gatcccgtagatcgctaccga

ggacactcatcgggcgtagatcgaagtgtgtgtgcgatctatcgggaggtcagcgaacgc

gcgctcggcacgaT-GCGTGAGTGc----C-GaT--CG----------------------

----------------------------------A--GGggaaagatatggcgcgcgctt

gagaaaggtcgtggaacaaagtggtggcaaaattgtttacgagttatt------------

----------------------------------A--ATAATA----AAcaa----Gg-A

--CT---Ttccaatcccatcgt-----------TT----------------Tg-A-----

--AG--GAAgt----AACGC--G--Ttaacg-----TT-TC--TT-----AG-----Cg-

---------Ttcttcgaaagaaaac--G---AAAATATAACA

>GYPSY24_LTR_AG

TGTTAT-A--

---------T--------ATGAA-CccT---G----gT-A--------------------

-------------T-TTGACAGTT-----GtG-T--CAtaacgcctg-------------

----------------------------------Ac-AGgttgaccctacctttcgtttg

ttgttaaatgtcattccttctttacaccttattgaattcgtttggttactt---------

----------------------------------T--TTGCTA----CA-------Ac-T

--CC---T-------------------------TA-------------------A-----

--AC--AAAcct---CATGT--G--Aac--------TT-TT--CTgcc--TA-----A--

---------Taaagagaatt-------A---AATACATAACA

>GYPSY64_LTR_AG

TGTTGGcA--

---------G--------GACAG-C------C--CAcT-Gtgcggtgtgaaccccttcgg

ctagcagtgcgtccacctgcatttctatacgcatctcggggtgaca--------------

-------------A-GTGACAGCTgc---C-G-----G----------------------

----------------------------------A--TGccgggggcaca----------

----------------------------------Tg-GGAA-A----AA-------Gg-A

--GT---A-------------------------TTgtac------------A--Agagac

gcAT--GAGat----AAGGTa-G--T-----------G-AA--AT-----AA-----Ag-

---------Tacaa-------------G---AAAACGTAACA

>GYPSY23_LTR_AG

TGTTAT-A--

---------TacaacattGACAG-C--TatgG--CA-T-Gttggcagctc----------

-------------T-GCGACTGCT-----C-G-----A----------------------

----------------------------------AcgAGcagcactgctcgaaacaggaa

gggagcgaatgaaatgtc--

----------------------------------A--TGCATA----CAa------Gg-A

--AC---T-------------------------GAat--------------A--A-----

--AG--AAAa-----CGCGT--G--Taacatt----TG-TA--CT-----GC-----A--

---------Tc----------------A---AAAATACAATA

>GYPSY25_LTR_AG

TGTTAT-Ggcccaacctgcctaacggctaagggcactcaccgaagacaggcaatggtctt

cgggtgatgtg---

---------T--------GAGTG-T--A---G--CA-G-Gtcgggcc-------------

-------------A-GAGAGAGAGccgagC-G-G--CA----------------------

----------------------------------A--AGtgcattattatactgtaggct

atcgcacgggcaacaagaggag--------------------------------------

----------------------------------A--TAGATA----AA-------Gg-C

--AT---A-------------------------TTccata-----------T--Attc--

--AG--AAAgacg--AAAGT--G--Tac--------TG-TA--TT-----CG-----A--

---------Tagaagggtggc------G---ATATCACAACA

>GYPSY62_LTR_AG

TGTTGG-A--

---------T--------CCCAG-T--T---Ac-CA-C----------------------

-------------A-GGGATA-AT-----G-GaA--CAcatagcaacacttagaaagcat

agcaaccatgcggtataaaaggagccgatagctcatcaccgctttactctg---------

----------------------------------A--AGttgatttc-------------

----------------------------------A----AATA----CA-------A--A

--AC---T-------------------------TT----------------A--A-----

--CTt-GAA------CCTGT--G--T-----------G-TA--ATagtc-CG-----Ag-

---------T-----------------AgttCGGACATATCA

>GYPSY3_LTR_AG

TGTTGC-A--

---------T-----------AG-T--A---A--CA-C-Gcataacgcagtaacat----

-------------T-GCAAGA-CT-----C-GaT--CA----------------------

----------------------------------G--AGtacacattg------------

----------------------------------Ag-TGAATA----AA-------G--A

cgAT---Tccattctgaac--------------TA----------------AggA-----

--AT--AAAg-----CAGTT--G--Tgt--------TT-TT--CT-----CA-----Aga

tatattcccT-----------------G---CGA-CATATCA

>GYPSY69_LTR_AG

TGTAGA-Gaataatagtaataataataacaataataataatttagaaattaaatttaaaa

taaaatgattgttaccattaaagtgaagcttagaattgatcgcgagagggaaaggaaaag

agagaaatagcgatagaacgagaagcgtatcgacaagtgagagactcgcggcttgccgac

aagtgaacgT--------GAGAGaC--T---G--CA-C-Ggtgagatgctacacggtgag

aagagttcaacatagaaacgcgttttcaagagaggagcaactcggtgagatcgca-----

-------------T-GTGAGAG-------C-G-G--CAggatggaaacgaaacgcgaaaa

ggcgatcagtttttggacagacgtagaaaagac---------------------------

----------------------------------A--AG---------------------

----------------------------------Tc-GCAATA----AA-------A--A

g-AG---T-------------------------GTcccagcagtaaaaaaaA--A-----

--AA--AAAa-----AAAGT--G--Ttaaaa-----TG-TA--TA-----AC-----A--

---------TcaatgatgacaagggctG---CGGTTACGACA

>GYPSY21_LTR_AG

TGTAGT-Aggctatgactacta--------------------------------------

---------T--------GACTA-CcaA---A--CA-G----------------------

-------------T-GGGATTTTG-----C-C-T--CA----------------------

----------------------------------Gc-AGaatttcacttcctaggcatag
tagcctatgccatgttt---
----------------------------------A--GTAATA----AA-------Gc-A
--GT---Tcatag--------------------TT----------------A--Acc---
--ACc-AAActag--CAAGTtgG--Tt---------TT-TA--TT-----TG-----C--
---------Tctctgtgt---------G----AACTGTAACA
>GYPSY60_LTR_AG
TGTTGT-Ggtcacgaagtggaaggctccctttattcggcgt-------------------

---------T--------AAGAA-C------G--TA-T-Gcagccactg-----------

-------------A-GTGACTGATgag--C-GgT--TA----------------------

----------------------------------Gc-AGacgagagagcgccagacgctc
gtgggacaccgtcgggaacacaagggctgatcg---------------------------
----------------------------------T--GTAATA----AA-------G--T
--AT---TgtgtattgtttattacgtgttcggtGT----------------A--A-----
--ATa-TAA------ACTGT--G--T------------aTA--CTctcggCC-----A--
---------T-----------------C---CGAACACAACA
>GYPSY67_LTR_AG
TGTTAT-Ggtgaatattttgccttagtgagccaacctgtatgtatcacggctctcactga

---------T--------GAGTG-C--Ac--G--CA-T-C--------------------

-------------TcGTGAGA-TGc----C-G-T--TAggttcgggcttatgacgtgatc
cccgaaggatcactctctctccggcgcgcctccaattagg--------------------
----------------------------------At-AG---------------------

----------------------------------T---GAATA----AAc------G--T
--TT---T-------------------------TAtatgtgt---------T--A-----
--AC--TTAtt----CGCGT--GcaTtcgtccacc-TC-TT--TT-----AA-----A--
---------Tgcatgacga--------C---AGACCTTAACA
>GYPSY63_LTR_AG
TGTTGG-A--

---------Tat------CACAG-T--Ac--A--CA-T-Gtacaaccatatagaat----

-------------A-GTTATTGTA-----C-GaT--CG----------------------

----------------------------------T--AGtatacatttataaccttatag
aatagtcagt--
----------------------------------T--GGAATA----AA-------Gc-T
--AT---T--A-----
--ATctGAAc-----ACATC--G--T----------AG-T---CTgg---CA-----Ac-
---------T-----------------A---AGTATATAACA
>GYPSY68_LTR_AG
TGTGGT-A--

---------Tgt------GAGAG-T--A---G--CAgT-G--------------------

-------------T-GGGTGTGCG-----G-G----CA----------------------

----------------------------------Gt-AGgaggttgg-------------

----------------------------------Ac-GGAATA----AA-------G--A
--GC---A-------------------------GAcgtgtg----------T--T-----
--ACt-GTAgtt---CCGGT--G--T--------------T--TT-----CG-----A--
---------T-----------------G----GAATATCACA
>GYPSY20_LTR_AG
TGTTAT-A--

---------T--------CTGAG-C--T---A--CA-C--ttctggcagcactggagctg
tcatacgctattccccatacacaccgtggtatgaacaactaccttccatacacaccgtgg
tatgaat------A-GCTAGA-TT-----C-T-A--TAtacactgcgatcgcttccgcta
ccgttctacgacacttgaagaagagaagccgaactac-----------------------
----------------------------------A--AGcactac---------------

----------------------------------A--GGACTA----CA-------Aa-T
--AT---A-------------------------TTtaga------------T--Agag--
--AT--AAAacagttCGGTT--G--Tacaa------TT-TA--CT-----TA-----A--
---------Ttacggttccgcc-----C---CATCTACAACA
>GYPSY65_LTR_AG
TGTGGT-A--

---------Tgtgttgt-AAGAG-C--Tcg-G--CT-T--tattccgctcct--------

-------------T-GCTAGAGCG-----C-G-G--TAtaatctgacaacgatgacag--

----------------------------------At-AG---------------------

----------------------------------Tc-AGAACA----CA----------T

--AC---A-------------------------GA-------------------Acgg--

--AGt-GAAgcc---AGATC--G--T-----------G-TA--TT-----AC-----Agc

aagc-----T-----------------A---C--CCACCACA

>hmmcons

TGTTGT-A--

---------T--------GACAG-C--T---G--CA-T-G--------------------

-------------T-GCGAGAGTT-----C-G-T--CA----------------------

----------------------------------A--AG---------------------

----------------------------------A--GGAATA----AA-------G--A

--AT---T-------------------------TT----------------A--A-----

--AT--AAA------CAAGT--G--T----------TT-TA--TT-----CA-----A--

---------T-----------------G---AGAACATAACA

…………………………

Retrofit_150

>RETROFIT_LTR

TG----------TGAA-----------------A--CAG----TctgttctA--T-----

--T--ATCC-Tggacatgagatataca-----------T---GCAT--Gctccagtacat

gcgatgctctag--

--------Tac---------Tg-GGAT-AtgG----------------------------
----------T-Tc-----GT-TA-Gg-ATAag-------GA-Ga------T-T-Atctc
caa---A-T---T------TGT-----TTcacctTC----Tta-----------------
---------TCca---T----------TGT-A------A---Ctga----A---------
--TAC--------TGT
A-Ag---CCA-C---Ggtaga-----G--GCTGTTtCTa-CCaC---TATT-----T-A-
AC----------Agaga--T-GCGG-CC----CAA-G----G------------Caa--A
-G-G---GTT---TA--A--C-----GC-TT----------------CAcA-TT-----T
taTCA--------------------------------CA
>RETROFIT4_LTR
TG----------TGAA-----------------A--CTA----T-------A--Ttg---
--T--ATAC-A---------------------------T---GCGT--G-----------

--------Tgaga-------Tc-TGTT-A--G----------------------------
----------A-Ttg----GA-TAaG--ATA---------GA-Gt--T-TGT-T-A----
------G-G---T------TGT-----TAgcc--TC----Ttcta---------------
---------TCca---TcaaaacagacTGC-A------Tg--Ct------A---------
--TATgga-----TGT
AaAaa--CCA-C---Ggtgat-----G--GTTGTTaTCa-CC-A---TATA-----TtA-
AC----------Aaagg--T-GCGG-CC----TCT-A----A------------C----G
-A-G---GTT---CA--A--C-----GC-TTCtgc-------AcC-TCTgA-TT-----T
--TCA--------------------------------CA
>COPIA-43_SB_LTR
TG----------TTGA-----------------G--ATA----Tg------A--Tgactt
g-T--AACT-Acttgtactagc----------------T---ACTT--G-----------

--------T-----------Ac-TAGA-A--G----------------------------
----------A-TatgacgCA-TG-T--ATCtagc-----TA-Gg--T-GGT-T-A----
------T-C---Taga---TGA-----GA-----TC----Tttgagagt-----------

---------TTg----T----------TGT-A------A---Ttc-----A---------

--TCTtcttgtgcTGT

A-Ag---CCA-T---Gcagag-----G--GCTATTcTCt-GC-A---TATA-----TtA-

AC----------A------T-GCAA-CC----GAGaG----C------------Ccca-A

-A-G---GGT---CA--T--C-----TC-GTT----------CaC-CCT-A-AT-----T

--TTA--------------------------------CA

>COPIA-17_SB_LTR

TG----------TTAA-----------------A--GTA------------Ac-Ttg---

--T--AACCcA---------------------------A---GTCTa-G-----------

--------G-----------Tc-GGTTgA--G----------------------------

----------A-T---------TA-Gg-ATC----------------T-TGT---A----

------------T------TGT-----TA-----TCcc--A-------------------

---------TG-----T----------TGT-A------A---C--------cttgttgta

ctcttagttagttatccaagatctcaaccgacctaacca-------AAT--------TGT

A-Ag---CCA-C---Gtgaga-----G--GCTCTTtCTc-AC-C---TATA-----TaA-

AC----------Aaagg--T-GCGG-CC----TCC-T----A------------Cg---G

-G-A---GGTt--CA--A--C-----GC-TT-------------C-CCAaA-TTa----C

--TTT--------------------------------CA

>RETROFIT3_LTR

TG----------TTAGttagcatgaaaggttcg----GA----Tc------A--Tg----

--T--GATC-Acgacctgacagct--------------T---GCAT--Gcatgtagttgt

tgaggttagtttgga---

--------T-----------Ta-GGTT-A--Gtctatccatgcggatcaggttagccttt

cctcca-------------CA-TGcG--ATAaca------GAtGt--T-TGT-T-Aga--

------G-T---T------TGT-----TT-----TC----Tg------------------

---------TTt----Tc---------TGT-T------A---C--------tgcatgcag

tcgtttcatcaatctcggcttcggttgttctcagctgcgccga---ACTc-------TGT

A-Tcg--CCA-C---Gatggg-----G--GCTGTTcCCa-TCaCataTATA-----TtA-

AC----------A------C-GAAG-GC----GAC-G----C------------At---A

-G-GattAGTgc-GA--T--C-----GT-TTC----------CaC-CCA-A-ACtaa--T

--TCA--------------------------------CA

>COPIA-125_SB_LTR

TG----------TTAG-----------------A--CAA----Tagg----A--Tagtat

--T--GTCT-Tggtacattgaa----------------T---GTGT--G-----------

--------T---------------GCT-A--Ggcttagggttgttgagcacgccacacag

-------------------GT-TG-Tt-ATC----------A-Gg--T-TGT-T-Ata--

------G-CacaT------AGA-----TA-----TC----Taggagat------------

---------TTg----T----------TGT-A------A---C-------A---------

--AACatcaatc-TGT

AgAgg--CCA-C---Gtaggg-----G--GCTCTTcCCt-AC-C---TATA-----T-A-

AC----------A------T-GCA--CC----GAGaGtt--C------------Ct---G

-G-At--GGG---CA--A--Ctc---TC-GTT----------C-T-CCT-AgTT-----T

--TTA--------------------------------CA

>COPIA-26_SB_LTR

TG----------TTAG-----------------Aa-ATA----Gg------A--Tccat-

--TgcATCT-A----------------------------gacGCAT--G-----------

--------Gcgtctc-----Tt-GACT-T--Gtactcaaagtaaatc-------------

----------A-Tg-----CA-TG-Ta-ATTagcc-----TA-Gt--T-AGT-T-A----

------G-C---T------AGGa----GA-----TAga--Tatttctgttttgg------

---------TTg----T----------TGT-A------At--CtcctgagA---------

--TCT--------TGT

A-Aa---CCA-C---Gcaagg-----G--GTTGTTcCTt-GCcC---TATG-----TtA-

AC----------A------C-GCAG-CC----GAGtG----C------------Ctc--A

-A-A---GGGc--CA--A--C-----TC-GTT----------AaC-TCC-A-T------T

--TTA--------------------------------CA

>COPIA32-ZM_LTR

TG----------TTAG-----------------At-AGA----Ttct----A--Tgattc

a-T--GTTT-Atcttcct--------------------T---GTAT--Gcttatcccctt

gtaactcggaacaaaccagtagagtcggcca-----------------------------

--------------------Ag-GAAT-A--G----------------------------

----------A-T---------TA-G--ATTgg-------TT-Tat-T-TGT-T-A----

------G-C---C-------CT-----TA-----TC----Ttggaccgaagatatc----

---------TCatggtT----------TGT-T------A---Cg------T---------

--TAA--------TGT

AaA-----TC-T---Gaa--------Gc-GCGCTT-TGt-CG-C---TATAag---T-A-

AC----------A------C-GCAG-ACa---GCC-A----Cgg----------C----A

-A-A---GGT---GA---g-Ct----GC-TTC----------AcC-CCA-A-TC-----T

--TC-cgcgcctctgattttctatatggtatcggagcCA

>COPIA6-SB_LTR

TG----------TTAG-----------------A--ATA----Gca-----Ac-Taactc

--T--GTAA-A---------------------------G---ATAC--Ggcaaatgaccg

taatcaacttggctacgtccaggttctaggtggt--------------------------

--------T-----------Tc-GGTT-A--G----------------------------

----------AgTa-----GA-TA-Tg-ATCtt-------TA-Gt--T-TGT-T------

------G-----T------AAT-----TA-----TC----Tcaacggaaacctcat----

---------TC-----T----------TGT-A------A---Cac-----A---------

--AATt-------TGT

A-Aa---CT---------------------CTATAt-------A---TATA-----TgAa

AC----------A------T-GGAA-CC----GTG-GgatgC------------Ca---A

-G-At--AGG---CT--Ac-C-----AC-GTT----------AgC-ACA-A-TT-----C

--TTT--------------------------------CA

>COPIA-55_SB_LTR

TG----------TTAG-----------------A--ATA----T-------Ag-Tagctg

--A--AAAT-Agtgtgtgtgttagcgg-----------T---GCAT--Gttagtgtgttg

aatatgttagaagcatgacacatggcacaatattcagcagta------------------

--------T-----------T--GAAT-A--Gacgccatac-------------------

----------A-Tg-----CA-TA-Gg-CTAgaa------GA-Gc--T-TGT---A----

------G-A---T------TAAgtc--AAg----TC----T-------------------

---------TTa----T----------TGT-A------Aa--Cg------A---------

--TCTcca-----TGT

AcAg---CCA-C---Gaaggg-----G--GCTCTTcCCt-TCaC---TATA-----TaA-

AT----------A------C-GGAA-CC----GGG-A----A------------Cc---A

-G-Gat-AGT---CA--T--C-----CC-GTT----------AcCtCCT-A-TT-----T

--CTAtaggcactatgtgt------------------CA

>COPIA21-ZM_LTR

TGactggggttgTTAA-----------------G--ATA----Tg------A--Tgct--

--A--ATCC-Tgataacgcgtgtgttatca--------G---ATAT--A-----------

--------G-----------Tc-AGTT-A--Gtaatatcctatattttgggac-------

----------------------TA-G--ATAcggca----GA-Gt--T-TGT-TcA----

------G-----C------AATacataGA-----TCc---Tgtaggtcccatc-------

---------TTaaaggT----------TGT-T------A---C-----------------

--TCA--------TGT

AaAt---CTA-A---Gcatc------------ATTgCTt-TC-C---TATT-----T-A-

AT----------AgaatcaT-GCGG-CC----TCG-A-----------------C----A

-G-A---GGTga-GA--A--C-----GC-TTC----------A---TCA-A-TT-----T

--CTA--------------------------------CA

>COPIA-127_SB_LTR

TG----------TTAA-----------------A--AGG----Gtg-----A--Ta----

--T--ATCC-Aactcctcattctt--------------A---GCTT--G-----------

--------T-----------Tc-GGTTg--------------------------------

------------T--------gTA-Gg-ATA----------T-GtccA-TGT---Aca--

------T-T---T------TCTg----TT-----TC----Tt------------------

---------TC-----T----------TGT-A-------------------ttgtagtcc

acggctgcatgttggcatgccggttagttaggggctgcggctcattACT--------TGT

A-A-----CA-C---Aatcta-----G--GTTGTAcCCt-AC-Cag-TATA-----T-A-

AC----------A------T-GTAC-CC----GAGtA----C------------CtatgA

-G-A---GGT----A--T--C-----TC-GTT----------CcA-TCT-A-CT-----T

--TCA--------------------------------CA

>COPIA-123_SB_LTR

TG----------TTAA-----------------G--ATA----Tcag----A--Ta----

--T--GTAT-Tgtcctgtaa------------------T---GCAT--------------

--------------------Tc-GGTT-A--Ggatctagataaggtt-------------

-------------------GT-TA-G--ATT----------AtGt--T-TGT-T-A----

------G-C---C------TGA-----TA-----TC----Tcatccaaccgaatg-----

---------CC-----T----------TGT-A------Ag--Tc------A---------

--AGTt-------TGT

A-A------------------------tcGCTATAt-------A---TATA-----TcA-

AC----------A------C-GTAC-AC----GTG-AtgagC------------Ca---A

-G-At--AGG---CA--At-C-----ACgGTT----------CcA-TCT---TCg----T

--TTA--------------------------------CA

>COPIA-134_SB_LTR

TG----------TTGA-----------------A--ATA----Ttt-----A--Tcc---

--A--GTCC-----------------------------A---AGAT--G-----------

--------Tatacgc-----Aa-GGCT-A--Ggctccgtgtaacaatagaatctaggagg

ttgttgtaaa---------CT-TG-T--ATTgtaagt---TA-Gg--T-TGT---A----

------G-----A------TAT-----GA-----TC----Taatgttctac---------

---------TTg----T----------TGT-A----------C-------A---------

--AGT--------TGT

A-Ag---CCA-C---Gctagg-----G--GCTCTTcCTa-GCaA---TATA-----T-A-

AC----------A------C-GGAG-CC----GTGtAg---C------------Ct---A

-G-G---AGG---CA--AtaC-----AC-GTT----------A-A-CCC-A-A------T

--TTA--------------------------------CA

>COPIA-98_SB_LTR

TG----------TTAA-----------------A--CGA----T-------Gc-T-----

--A--ATCC-Tttcggttag------------------G---ATCT--G-----------

--------G-----------T--GTCT-A--G----------------------------

----------A-Tt-----CA-TT-Gt-ATAggt------TT-Gga-T-TGT-T--gtac

aagtagG-T---T------TGTtga--GA-----TCca--A-------------------

---------CCa----A----------TGT-A------At--Ctgtcc--T---------

--TGT--------TGT

AaAcg--CCA-T---Ggagag-----G--GCTGTT-CTc-TCcC---TATA-----T-A-

AC----------A------C-GAAG-CC----GAG-Gac--C------------Ct---G

-G-Aa--GGG---CA--Ac-C-----TC-GTT----------A-A-CCC-A-TCtcgt-T

--TTA--------------------------------CA

>COPIA-104_SB_LTR

TG----------T-AA-----------------A--ATA----G-------A--T-----

--A--GACT-----------------------------T---GCAT--G-----------

--------G---

-------------------CA-TG-Ca-AT-----------------------T------

------G-G---

----------------T----------TGT-A------A---Ca------A---------

--ACCagacg---TGT

A------CCA-A---G----------------ATT-------------------------

--------------------------------GAGcT----A------------C----A

-A-Gtt-AGG----A--A--T-----TA-GTTatt-------AtA-CAT-A-T-------

-------------------------------------CA

>COPIA-136_SB_LTR

TG----------TTAA-----------------G--ATA----Tgt-----A--Ta----

--A--GACT-Ttgagttgtatacgacatggcttcgt--T---GTAT--G-----------

--------Taatc-------Ta-GAAT-A--G----------------------------

----------AcTt-----G--TAcG--ACA----------A-G---T-AGA-T-Acacg

------G-G---A------TCTactc-GA-----TC----Tctta---------------

---------TCag---A----------TGT-A------AtttCt------T---------

--TCTtga-----TGT

AcAag--CCA-Cca-Ggg--------G--GCGTTT-CCc-TGaC---TATA-----TaA-

AC----------A------C-GGAG-CC----GAG-Gt---A------------Cag--A

-G-A---TGT---CA--C--C-----TC-GTT----------CtAgTCT-A-T------T

--TTA--------------------------------CA

>COPIA-54_SB_LTR

TG----------TTAG-----------------A--AGA----Tag-----Ag-Ttg---

--T--ATCC-----------------------------T---ATAT--G-----------

--------G-----------Aa-GCCT-A--G----------------------------

----------A-Ttactt-GA-TG-T--ATAc--------TTc-----aTGT---A----

------C-T---C------TAT------A-----TA------------------------

------------------------------gA------Att-Cc------T---------

--TGT--------TGT

AcAagg-CCT-T---Gtatg------Gt-GCGATTgCAc-CC-A---TATA-----T-A-

ATg-----------------aGATG-CC----GAGtA----C------------Cct--G

-A-G---GGG---TA--CggC-----TCcTTTg---------C-C-TCT-A-TTctc--T

--TTA--------------------------------CA

>COPIA27-ZM_LTR

TG----------TTAG-----------------Ac-AGAattgTa------A--Tccc--

--T--ATCC-Tctgg-----------------------T---GCCTcgG-----------

--------G-----------T--GGTT-A--Gttggttagc-------------------
-------------------CA-TA-G--ATA---------GA-Ga------T-T-A----
------G-G---C------TATa----GA-----TA----Aagataagcc----------
---------TCggc--Tg---------TGT-A------At--C--------ttaaccacc
tgaaactc--------------------------------------TATccg-----TGT
AtAcaccCTT-C---GcccggtatacG--GCG-----------C---TATA-----TaA-
AC----------A------T-GCAA-CCt---GCG-Gcca-C------------Ca---A
tG-Gt--GGTt--GA--A--C-----GC-TTC----------A-T-CCT-A-TTagg--T
--TTA--------------------------------CA
>COPIA-65_SB_LTR
TG----------TTAG-----------------A--ATA----Cc------Ag-Ta----
--T--GTTC-Agcatacgtacatgacaactatggt---T---GTGT--G-----------

--------T-----------Tg-GAGT-At-G----------------------------
----------AgTcagg--CA-TA-G--ATAcggtttcatGT-G---T-TGT---Atac-
------G-A---T------TAG-----GA-----TA----Tcctc---------------
---------TTg----T----------TGT-A------At--C--------ctattattg
taatctcctagattagatacttgttg--------------------TACaaga----TGT
A-Aa---CTC-CtttGccaaa-----G--GATATTcCTg-GC-C---TATA-----TaA-
AC----------A------T-GGAA-CC----GTG-G----C------------Ctg--A
-G-A---AGG---TAccA--C-----GC-TTT----------A-A-CCA-A-A------T
--TCA--------------------------------CA
>COPIA-23_SB_LTR
TG----------TTGA-----------------A--ATA----Tg------Ag-Ttg---
--T--GTTT-----------------------------G---GTAT--Atcgtggaggca
aggcacaacacgtcaaggctgttagcgtgacttgaattggttctcaagtaaacccggctc
catgtatcT-----------A--GTCT-Ag-G----------------------------

----------A-Tt-----GT-TG-Ta-ATC----------T-G---T-TGT-A-A----
------A-G---T------AGTtgtagAT-----TC----Aatc----------------
---------TC-----T----------TGT-Aca----At--Cc------A---------
--ACC--------TGT
AtAcg--CTA-CctaGg---------G--GCTATTcCTa-GGaC---TATA-----T-A-
AC----------A------T-GGAG-CC----GTGaGag--C------------Cg---A
-A-A---GG----CA--T--C-----AC-GTT----------AaA-CCA-A-TC-----T
--TTA--------------------------------CA
>COPIA-88_SB_LTR
TG----------TTAG-----------------G--AGA----T-------A--T-----
--A--ATTT-Tacacgagataaacctt-----------T---GGTT--G-----------

--------G-----------TgcGGCA-Ag-G----------------------------
----------AcT------CC-TA-T--ATT----------A-Gga-T-AGT-T-A----
------AaC---A------TAG-----AA-----TC------------------------
----------T-----T----------TGT-A------A---T--------ttctaattc
aggagtcctagcctaaatcacata----------------------TATc-------TGT
A-A------------------------------TA-TTg-GTtC---TATA-----TaA-
AC----------A------C-GGAC-CCtatgGTG-A----G------------Cc---A
-A-Gtt-AGG---CA--A--Cca---TA-GTT----------A-CtCCAtA-TT-----C
--TTT--------------------------------CA
>COPIA-116_SB_LTR
TG----------TCAA-----------------AtgAAA----T-------AggTcta--
--T--GTCT-A---------------------------G---GTTC--G-----------

--------Gtgta-------Tc-TTCT-A--G----------------------------
----------A-Tgag---GT-TAaG--ATCc--------TA-G----aTCA-T-A----
------A-C---TaacagaTGT-----AA-----TC----Ttcattag------------
---------TTg----T----------TGT-A------A---Ca------A---------

--ACTtgtct---TGT

AtAtg--CCA-Cc--Gccgtgga---G--GCTGTTtCCc-GG-Ct--TATA-----TaA-

AC----------A------C-GGAA-CC----GAG-A----G------------Cc---A

-G-GcaaAGG---CA--T--C-----TC-GTT----------A-A-CTC-A-TC-----T

--TTA--------------------------------CA

>COPIA35-ZM_LTR

TG----------TTAA-----------------G--CTA----T-------A--T-----

--T---TCT-A---------------------------Tg--GCAT--G-----------

--------TataattatagaTa-GAAT-T--G----------------------------

----------A-T-------T-TA-G--ATTtcc------TT-Gta-T-TGT---A----

------AcC---A------AGTcca--TA-----TC------------------------

---------------------------TGT-A------A---Cg------A---------

--TCTgtaact--TGT

G-Tg---CCA-A---Gttt-------G--GCT--------GG-C---TATA-----T-A-

ACcatc--------------gGCAA-CC----TCC-T----G------------Gg---A

-G-A---GGT-------A--Ct----GC-TTTctgcatatt-CtCtACT-A-TT-----C

--TTA--------------------------------CA

>RETROFIT2_LTR

TG----------TTAG-----------------A--TAA----T-------G--Tt----

--A--GT-T-----------------------------A---GTTT--G-----------

--------T-----------T----AT-A--G----------------------------

----------A--------GA-TA-G--ATT----------A-Gtt-C-TGT-T-Act--

------G-C---A------TGTac---TT-----TC----Ttttatctatctctatatcc

aggattgtcTCagg--T----------TGT-TgagattAgt-Cct-----A---------

--TCCtt------TGT

AcAcg--CCA-C---Ggcaga-----G--GCTCTT-TCt-GT-C---TATA-----TcA-

AC----------Aaagg--T-GCGG-CC----CCG-T----A------------Ca---G

-G-G---GTT---CA--A--C-----GC-TT----------------CTcA-TTccgt-T

--TTA--------------------------------CA

>COPIA28-ZM_LTR

TG----------TTGG-----------------Aa-AAA----Tag-----Ag-Tt----

--T--GTTT-A---------------------------A---GTAT--Gttgcgtaaata

ttggtttgtttcctgacgcacctcttcct-------------------------------

--------G-----------A--GGCT-A--G----------------------------

----------Aa-------GA-TAgGa-ATA---------TA-Gt--T-TGT-T------

------G-----A------GGT-----GA-----TA----A-------------------

----------Cgc---T----------TGT-A------A---Cagaa---A---------

--TATgt------TGT

A-At---CTC-C---G---------------GCTTcCTttGC-C---TATA-----TtA-

AC----------A------T-GCAA-GC----TCG-G----CagagaacaaatgCt---A

-G-A---GTT---TA--At-CaaaagGC-TTCcgct------C-C-TCTgA-TT-----T

c-TTT--------------------------------CA

>COPIA40-PTR_LTR

TG----------T-AA-----------------G--CAA----G----------------

--T--ATCC-A---------------------------A---GTAT--Ggaactaactcc

aactgaacctcct---

--------G-----------Aa-GAAT-Ag-G----------------------------

----------A-Tttaa--GC-TA-GtgATA----------A-Gat-T-TGTaT-A----

------G-A---A------TGG-----AAc----TC----Taattattgtgtttga----

---------TTagaacTc---------TGC-A------A---Catt----A---------

--TCTttat----TGT

AtA--TATA-----T-A-

ACctctgattcaA------T-GAA--------TAA-A----C------------At---G

-A-G---TTT---CT--A--C-----GT-TTC----------AcAaTCAgA-TTtcatcT

--CCA--------------------------------CA

>HOPSCOTCH_LTR

TA----------TTAG-----------------Ac-AGA----T-------A--T-----

--A--TTCC-----------------------------A---GATT--G-----------

--------T-----------A--ATCT-T--Gatttctttccaactaaccggacgtagat

agcacagg--A-Tct----CC-TT-G--ATTt--------TT-GgcgC-TGA-T-Atc--

------G-C---T------TGT-----GA-----TAcggcAg------------------

---------CC-----Tc---------TGT-T------Ag--C--------ctccggtta

gagtttgg--------------------------------------TACga------TGT

A-Acaa-CCT-Cc--Gc---------T--GCT-TTgTCa-GC-C---TATA-----A-At

AC----------A------T-ATAC-GC----GGCtG----Ct-----------Ca---A

-G-Ggc-AGTagaGA--A--C-----GC-TT---------------CCTaA-TT-----C

--CTT--------------------------------CA

>COPIA1_ZM_LTR

TG----------TTAG-----------------A--AGA----Taag----A--Ttagat

tgT--AAAC-Aaa-------------------------T---ATAT--Gtaacaaacaag

agtaagcatc--

--------Tc----------T--GGGA-A--G----------------------------

----------A-Taaa---GA-TAgGg-ATA---------TT-Ctg-T-TGT---A----

------A-C---T------AGA-----AT-----TCc---Tgaagcttc-----------

---------TCtagt-T----------TGT-T------A---Cg------A---------

--TCC--------TGT

AaAt---CCTgC---Gca--------T--GTTGTT-TAt-GC-C---TATA-----TaA-

AC----------A------T-GTAGg------GCG-G----C------------CctccA

-AcG---AGG---CAgcA--C-----GC-TTTaacctaatgcC-C-TCTgA-TT-----T

--TCA--------------------------------CA

>COPIA-126_SB_LTR

TG----------TTAG-----------------A--AGA------------A--Tt----

--A--GTCC-----------------------------A---GCAC--G-----------

--------T-----------T---TGT-A--Ggggctacggttgtacagttgtaccgtgt

----------A-T-------A-TG-Ga-ATA----------T-G-----TGT-T-A----

------G-----T------TGA-----GA-----TA----Tattaggaata---------

---------TCaag--A----------TGT-A------At--Ccc-----A---------

--TGCtttg----TGT

A-AcctgCCA-C---Gatggg-----G--GCTCTT-CCc-AT-C---TATA-----T-A-

AC----------A------C-GCAA-CC----GAG-Gatc-C------------Ct---A

-G-G---GGT----A--Ac-C-----TC-GTT----------AaC-CCTaA-AT-----T

--TCA--------------------------------CA

>RETROFIT5_LTR

TG----------TTAG-----------------AtcTTA----Tc------A--T-----

-----ATCT-TatcattctctgtatggatatgcacggaT---GCAT--G-----------

--------T-----------T--GGTTc--------------------------------

-------------g-----GA-TA-G--ATA---------GA-G------GA-T-A----

------GgA---C------TCT-----AA-----TC------------------------

----------Cc----T----------TGT-A------A---Tt------Aggatcaccg

aaccaacacatctgcacgcatata----------------------TATc-------TGT

A-Aa---CTC-A---Gc---------Gc----GTT-TTt-GC-A---TATAgtgaaTcA-

AC----------A------C-GAAA-CC----CTGcGt---C------------C----A

c---c--GTG-------A--C-----GC-TTTcctgcat---AcA-CCTgA-GT-----T

--TTA--------------------------------TA

>COPIA-135_SB_LTR

TG----------TTAG-----------------A--AGA----T-------A--Tg----

--T--TTCC-T---------------------------A---ACCT--G-----------

--------T-----------Ac-GGTT-A--Gctagtctaggctttc-------------

-------------------CT-TA-Ga-ATAgt-------TA-Gc--T-TGT-T------

--------C---T------TGTag---TA-----TA----Ag------------------

---------TTaac--T----------TGT-Atc----A---Ca------Agatgtaatc

tcccagatcacgttgtgatctgatc---------------------TGT--------TGT

AcTtg--CCA-Cc--Gtagg------G--GCTATT-CCt-ACgC---TATA-----T-A-

AC----------A------T-GAAG-CC----GAGaGacc-C------------Ca---A

-A-G---GGT---CA--A--C-----TC-GTT----------CcC-TGT-A-AT-----T

--TCA--------------------------------CA

>hmmcons

TG----------TTAG-----------------A--ATA----T-------A--T-----

--T--ATCC-A---------------------------T---GTAT--G-----------

--------T-----------T--GGTT-A--G----------------------------

----------A-T------GA-TA-G--ATA---------TA-G---T-TGT-T-A----

------G-C---T------TGT-----TA-----TC----T-------------------

---------TC-----T----------TGT-A------A---C-------A---------

--TCT--------TGT

A-A----CCA-C---G----------G--GCTGTT-CC--GC-C---TATA-----T-A-

AC----------A------T-GCAG-CC----GAG-G----C------------C----A

-G-A---GGT---CA--A--C-----GC-GTT----------A-C-CCT-A-TT-----T

--TTA--------------------------------CA

…………………………

Sire_150

>SHACOP21_LTR_MT

TGTT-AAAtga------------------------TAA----------------------

```
------------------------------------------------------------
----T-T-----T--GT----A---A---------T-T--T-------------------
----Tc-C--A-TAT--Tcc-----TA--G-GT-TCcttaa-TGTA---T---C-AG---
------------------------------------------------------------
------------------------------------------------------------
------------------------Ta--AGttcca-G-AG-AA-----------------
-----T--GT-CAT---t-TAA---TAtt------C------------------------
------------------------------------------------------------
-------------------------------CTAG-------G-TGc---------A-TT
C-Act-TA---TTTActagaactTT---------------C-T-A-------TG--Tatc
aatataactagagg----------------------------------------------
-----------------------------------T--T--CTA----------------
------------------------------------------------------------
------------------------------------------------------------
---T---------GTATTgacaaggcactacaa---------------------------
-----------T-G-AA-Aag-----A-------------------------A-------
--------------------ACATTac---------TTt---------------CAat--
---------------C-T--A-TAa------C-----------Tt---TcacGT--TT--
------T-----------------------------------------------------
------------------------------------------------------------
-----C--TG-TT---Cc------------------------------------------
--------------------T---Tttattcctctttct---------------------
------------------------------------------------------C--CAA
CA
>SHACOP8_LTR_MT
TGTT-AAAa--------------------------TTA----------------------
------------------------------------------------------------
------------------------------------------------------------
------------------------------------------------------------
```


----A-Tg----T--GT------------------T-G--Taacc---------------
----Gc-C--A-CTA--Tc------TT--T-AG-GA------AGCA---T---T-ATga-

------------------------T---AGaa----T-TG-TAaccatcat------C-A
T-A--A--TG-ATT--Gt-CTT---TA--------T------------------------

-------------------------------TTAG-------G-TT----------T-AT
G-G---TA---GGTTt-------GTta-------------T-C-AacctattTG--Tatt
ctctatcagtttccttct--
-------------------------------A---G--C--CTA----------------

---T---------ATAAAggctttgtaatgctaagt------------------------
-----------T-G-AAgT-------A-------------------------A-------
--------------------GCAGTa----------TGtggtgaatt-------CAa---
---------------C-A--A-AAt------Cag---------T----T---TT--TC--
-T----T---

-----CaaTG-TT---Ca--
--------------------T---Ccaccagtggaagtatagtataaaaagtcacgtttc
cactcaaaatcctgcaacagtataatttataacataccacctctagaatacca-C--CAA
CA
>CPSC4A_LTR
TG----GA---------------------------TAT----------------------

----T-T-----T--GT----A---A---------T-C--T-------------------
----T--G--A-GCA--Ttt-----TA--T-AT-TTc-----TGT----T---T-TGcat
gaacg---

------------------------T---AG------T-GG-----------------C-T
G-G--C--TG-AAG--G--TTG---T----------------------------------

-------------------------------TTTG-------G-CTggtcgg----T-AG
A-A---TA---GCTGt-------TTccttct---------G-C-A-------TG--T---

-------------------------------T---G--T--C--agttttgtttagctgc
cgaatgacctggtttcagatcgtgggatggcgcggtcagtagccgatccgggttgctcgt
tccgttcagttgtgcatgtaacaaactccgttttggtggaggaga---------------
-------------ATAAAgag---------------------------------------
-----------T-G-AA-A-------Acagaaa-------------------A-------
--------------------GCAGCagaag------TTgcgctg----------CAc---
---------------A-A--A-AAttcta--C-----------Tt---T---GT--TC--
-T----T---

-----C--AG-TT---Cttgacg-------------------------------------
--------------------T---Ttcccgaacttcttaaggttactgtcgttgtccggc
aattttcagagtgagcgagtgtgagcgatttcagagtttgttctttccgttttgC--TGA

CA

>COPIA-111_SB_LTR

TGTT-AGC---------------------------TAA----------------------

----G-Taga--T--GC----Ac--G---------A-G--Tc------------------

----A--T--T-CAT--G-------TA--A-TC-TTt-----TGTTa--A---A-AGgag

tcttagag--

------------------------T---TG------T-AG-GAaggcatga------C-C

G-G--T--GG-CGT--Gc-CGG--------------gtcatgagtctacttgtgtcggtc

agtttgttgacgccacgacgagtatcgtgcacgcggcgtatcttgcagggaaacgaggta

cgcacggtgcccgccgtg-------------GC---------G-TG----------G-TG

G-A---TC---GG-G--------GC---------------G-C-C-------TG--Tgct

cacgatcag---

-------------------------------A---G--T--CTA-----------------

----

---T---------TTATA--

-----------T-GcAA-T-------Aaagaggaccgaa-------------A-------

--------------------ACTGCgacg-------TTgggcag----------CAatta

gaac-----------C-C--A-AAt------CtgtcttcacgaT----T---GT--TC--

-Tc---Ttataaattcgctgttcaggtatcaccgacatcgccg-----------------

-----G--AG-TT---Ca--

--------------------T---Cgacatcgggcggtcttgcg----------------

--C--CAA

CA

>COPIA-100_SB_LTR

TGTT-AGA---------------------------TAAcccaggctctcgttcatggacg

ttcctatttttactcggtcttcctgaataagctcatggcgaac-----------------

----A-Ct----T--GTa---A---A---------T-G--Gccagac-------------

----G--C--A-TGA--C-------TT----GT-TCa-----TGCA---G---C-GGtca

tccaggt---

------------------------T---TG------T-AG-GA-----------------

G-A--A--GT-AGTt-G--TAG---TGgag-----A------------------------

-------------------------------TC---------G-CGcgc---------AG

C-G---TC----CTC--------GT---------------T-C-G-------TG--Gga-

-------------------------------T---G--G--C--acggaccagttcgggt

cgtggcatgcgcgtcgcgtccgtggtgaccggcatgcttctagtcgtgggatggcgcgac

aatggcggaggtgtgaaggtctcagcgtcaggccgagtggcctttgtaatcatc------

---T---------ATTTAttt---------------------------------------

-----------T-GcAA-T-------A-------------------------Gagtcaca

aaacc---------------GCTGCtac--------TTcgcag-----------CAc---

---------------C-A--A-AAg------C-----------Tc---T---GA--TT--
-T----Tacttccactgtgttcgcgtgtgttccatctc----------------------

-----G--TG-TT---Ccactgagc-----------------------------------
--------------------T---Ttcagtcatcttctctatctccggcgatgggcgccg
gcgagcaaaaga---CAA
CA
>COPIA9-ZM_LTR
TGGT-AGT---------------------------TTA----------------------

----TaT-----T--GA----T---T---------A-G--T-------------------
----G--G--T-TAA--G-------TA--T-GG-TAaac---TGCA---TgtcT-AGatt
gttgggatggcaacaga---

------------------------T---AAtc----T-TG-GA-----------------
-cG--T--GC-CAT--Ga-TTGattCA--------A------------------------

-------------------------------TCAG-------T-CTgtaac-----T-AT
A-Ta--TA---ACT--------------------------G-C-A-------TG--Tat-

-------------------------------T---T--T--CTA----------------

---T---------ATAA---

-----------T-G-AA-T-------Agagagacaacagaaa----------A-------

--------------------GCTGTcgc--------TTg---------------CAg---

CA

>COPIA-72_SB_LTR

TGTTtGAA---------------------------TAA----------------------

----AgTcc---T--GA---------------------gcT-------------------

----T--G--T-TAT--T-------TT--T-AG-TA---------A---G---T-AGaaa

ctagattagcaggctgtgc---

------------------------Ta--AGta----T-GG-CAtgtcc---------C-A

G-At-T--GT-TGG--Ga-TGG---CA--------Acaattgctcacgagcatgccgtga

ttgacacag---

-------------------------------TC---------G-AT----------T-GT

A-AtttTA---TTTCc-------TT---------------G-C-A-------TG--Taac

-------------------------------T---T--G--C--t---------------

---T---------ATAAAggaatgaaaggtctac--------------------------

-----------A-G-AA-A-------A-------------------------C-------

--------------------GCTGCcaca-------TTtg--------------CAgca-

---------------A-C--A-AAat-----C-----------Tc---T---GTc-TCc-

-Tg---T---

-----G--TG-TT---Cgcgtgtgttcttcctcacggaa---------------------

--------------------T---C-----------------------------------

--C--TAA

CA

>ATCOPIA78_LTR

TGTT-GAAagt------------------------TAA----------------------

----A-Ct----T--GA------------------T-T--Ttgaatcaa-----------

----G--T--T-TAA--T-------TA--T-TG-G-------ATCAa--T---T-ATcca

ataattaattatggccaaatccaagttctagagttttctctagaaatatcatcatttcca

cctccttaaaagattctagaaattttctagaatcatcttccacctccttaaacataaaaa

tctagatactctaatagaataatcT---AGataat-T-TG-AA----------------T

A-A--T--GT-AAT--C--TAG----A--------Tcttatgtaagaactctctagac--

-------------------------------TTAG-------G-AT----------T-AA
A-A---TA---TTTTag------ATa--------------T-T-T-------TG--Tagt
ttgg--
-------------------------------A---G--G--CTA----------------

---T---------AAATAcctcctccccctctcaaatgt---------------------
-----------T-GcAA-Tgttgtg-A-------------------------A-------
--------------------GTTGTa----------TTcaagtttaaag-----CAaagt
a--------------AtA--A-AAgtt----C-----------Ta---T----T--TC--
-C----Taaaaaactctctcaaaacacttaaacac-------------------------

-----T--TT-CT---Cca---
--------------------T---Tacctctaaaagaattttact---------------
--C--TAA
CA
>COPIA-14_SB_LTR
TGAT-GG-------------------------------tagttggtcatgtatctagtat
agcagggcctaagtattgtccactgcac--------------------------------

----T-Tt----T--GT------------------T-T--Tacttctctg----------
----T--T--T-TAAa-Tc------AT--G-TG-GC------TGTA---A---T-AGgct
tgcttgggggtcaaga--

----------------------------AGa-----T-AA-GAcc------------C-T

-----T--GG-CAG--Cc-AAGt--TA--------A------------------------

-------------------------------CTAG-------G-A-------------AT

C-T---AC---TCTAgaat----AT---------------G-C-C-------TA--Tcca

agaggcagccctc---

-------------------------------A---G--C--CTA----------------

---T---------AAGAGc---

-----------T-G-GA-G-------Acagggcaagg---------------G-------

--------------------GTTTTc----------TTg---------------TAa---

---------------C-Cg-AgATc------Ca----------Tt---T---TT--GCaa

-T----Tcaatcaaaaggccatagcaggcaaactgccctctggctgttgccagatcaatc

tc--

-----G--TG-TT---Caccaaa-------------------------------------

--------------------T---Tat---------------------------------

--C--CAA

CA

>COPIA-118_SB_LTR

TGTT-GGT---------------------------TAA----------------------

----G-C-----Tc-GA------------------T-G--T-------------------

----T--C--A-GGTg-T-------CA--T-GG-GActgcggTGCT---G---T-GGctg

gtttggtcagaggcgcagcagaatttgaatgaagccgatggcgggaacgg---------

-----------------------T---TG---------G-CGt-------------C-A

G-G--A--GG-AGTg-Gg-TGG---TG--------Accgcgctcacggcgtcgagcag--

------------------------------TTAGca-----G-TG----------T-GT

G-T---T----TCTGcc------GTcatgtatc-------G-T-G-------TG--Tgtg

agtagtt--

------------------------------T---T--T--C--attcaacaagtgtgtg

ttctaggtgtaggtataaatacctggatgtaagaggcatgagttagcacttaagacttgg

tgtaatcgtgtttga--

---T---------TTGAAagttatcaatag-----------------------------

-----------T-G-AA-A-------Aagggcactctctccc----------G-------

--------------------GTAGTtctccgggcagTTgc--------------CAaagt

gtgcgt---------C-C--A-ATt------C-----------Tcgt-T---GTgaTC--

-T----Tgcgcaatcttgagtttaggtttagccaccgt---------------------

-----G--TG-AT---Ctagcctgtga--------------------------------

--------------------T---Tgttgattctgag----------------------

---C--TTA

CA

>COPIA-41_SB_LTR

TGTT-AGTg--------------------------TAA---------------------

----G-Ct----T--GA----T---G----------------------------------
-----------gGGA--G-------TT--G-AT-GTt-----TGCT---G---G-AGacg
ctgcagcagctgggtaagcagaagcagcaaatttgaattcaaacaagagggagcgggagc
agttggctgagtgcagatggtgaccgcgcccacgttcgtgtctcaacgtcaag-------
------------------------C---AG------T-TG-CA---------------c-
-----T--GA-CAT--Cc-TTTtc-TA---------------------------------

-------------------------------TT---------G-TG----------T-AT
C-T---T----GCTTtc------GT---------------G-T-G-------TG--Tgtc
agagacttgtttagttg---
-------------------------------T---T--G--GTA----------------

---T---------AAATAgctaagtgtt--------------------------------
-----------T-G-GA-G-------Agcgc---------------------A-------
--------------------GTAGCgca--------TTg---------------TAagcg
a--------------A-Ct-A-TTtg-----Tg----------Tt---T---GT--GC--
-Tg---Ttcacatatatcagtgcaaaagagcactgtcctctgacagttgccagttcgtgt
ttcttcattgttcatgcgcattccttctctgtaggattagccaattgatcattagcgttg
tgattG--TG-AT---Cctcgg--------------------------------------
--------------------T---T-----------------------------------
--C--CAT
CA
>COPIA50-PTR_LTR
TGAT-AGAat-------------------------TAA----------------------

----T-Ct----T--GT----T---G---------T-T--Tt------------------
----A--T--T-TCA--G-------TT--T-GT-TT------TGTT---T---T-AAttc
cagcagagtttaagagtagacgtaacctttcatatagaagttatgatttagaggttatt-

------------------------T---AG--------TG-GAcgtaacctttcataT-A
G-C--A--GT-TAT--Ga-TT----TAgaggtta-T------------------------

-------------------------------TTA--------G-AGgacgtaacc-T-TT
C-G---TA---TTTGtaacc---TT---------------T-C-G-------TA--T---

-------------------------------TtgcT--T--CTA----------------

---T---------ATATAataagaaaatc-------------------------------
-----------A-G-AA-A-------Atg-----------------------At------
--------------------GCAGTgag--------TTaag-------------CAtc--
---------------A-C--A-AAaggc---C-----------Tc---T---GT--TC--
-T----Ttcattattttgtctctgtgattgtgt---------------------------

-----G--AG-TT---Tcccaaa-------------------------------------
--------------------T---Tgtgtgactggccgtaagaaagaataac--------

CA
>COPIA-46_SB_LTR

TGTT-GGTg--------------------------TAA----------------------

----G-Ccagc-T--GA----T---G---------T-G--Tcatg---------------
----A--C--T-TGT--Gacg----TG--G-AG-GAt-----AGCA---G---A-GGcgg
gagcgggagcgctgaagtttgaatgacccacgcacgggag--------------------

------------------------C---AG------T-TG-CAg-------------C-T
G----A--AG-CGG-----------TG--------Accacgtacacgatcgcgacgtcga
gaggcggttttcactgacaagcttatgta-------------------------------
-------------------------------TCAC-------G-TTtt--------T-AG
C-Agt-TC---TGTGt-------GTgtcaca---------G-T-G-------TG--Tgta
agcta---
-------------------------------T---T--G--GTA----------------

---T---------AAATAgc--
-----------T-G-GA-Tgttt---A-------------------------Gtgcgtga
gttagt--------------GCTTTggag-------TTggttg-----------TAaa--
---------------CtC--A-TTgaa----C-----------T----------c-TT--
-Tg---Tgaacagaggtgaaaaggggagccgacgccctgtcggcagtttct---------

-----G--TG-TT---Catccc--------------------------------------
--------------------T---Tgttcttgatcgattaggcctagccactgaaatctt

gctcgtgattgttgatccatca--------------------------------C--TTA

CA

>COPIA-33_SB_LTR

TGTT-AGT---------------------------TAA----------------------

----G-CctaatT--GC----T---A----------------------------------

----------T-GTA--Tc------TT--A-AT-TTc-----TGCA---T---T-AG---

------------------------T---AG------T-AG-CAgtgc----------C-T

-----T--GG-CAT--Cc-AAG--------------------------------------

-------------------------------TTTG-------G-AGc---------A-GG

C-A---TC---TTTAc-------TT---------------T-T-Gc------TA--Tagc

atattctggagtaggtgtctgaccaca---------------------------------

-------------------------------T---T--G--CTA----------------

---T---------ATAAGtggcagacccccctccctctattgtatcctgttatcacaagt

ttgat------T-C-AA-Tcc-----Aagcggccaagggcca----------A-------

--------------------GCTGCcctctgggca-TTcc--------------CAgat-

---------------C-C--A-AAt------C------------------------TC--

-Ta---T---

-----G--AG-TTggg--

---CAA
CA
>COP12_LTR_MT
TGTT-GAC---------------------------TAAttgacctataaaacttttctga
atactttattgtttatgttactttattgcttat---------------------------

----G-Tg----T--GT----A---A---------T-T--A-------------------
----T--T--T-TAA--C-------TT--G-TT-T-------TGT--------T-AGtaa
aactatgctgcacaagtaagattatctgcacaagt-------------------------

------------------------Ta--AG------T-AG-G---------------T-T
T-A--T--GT-CAT---a-TA----TAtgac----A------------------------

-------------------------------TCA--------G-CT----------A-AG
C-A---TAg--GCTGa-------GTcattactta------G-T-A-------T---T---

-------------------------------T---G--G--CTA----------------

---T---------AAGTAtgtaagaaggatgtatggatggta------------------
-----------T-G-TA-Tc------A-------------------------Aattt---

--------------------GCAGCa-----------Tag--------------TAa---

---------------A-C--A-TTg------T-----------Tat--T---GTc-CC--

-T----Ttctcttccatttttttctctaatttct--------------------------

-----G--TT-TT---Ctg---

--------------------T---Ttttgagcttattgcttctgctgccattacaaaag-

--C--TAA

CA

>COPIA-16_SB_LTR

TGAT-GGA---------------------------TAA----------------------

----GtCtc---T--GT----T---G----------------------------------

----G--C--T-GGT--Gg------TT--G-TGgGC------TGCA---G---T-AGgca

gcacattgtccttgtgtaggacagcatccttgactgcttttaggacagcagttagcatct

gtaggacagcatcttgactgcttttaggacagcagttagca-------------------

------------------------Tc--TG------T-AG-GAcagcatc-------T-T

A-GacT--AG-CAT--Ct-TAG-------------A------------------------

-------------------------------CTAG-------C-ATct--------T-GG

C-A---TAt--GCTGgc------TG---------------G-T-T-------AG--C---

-------------------------------A---Gc-C--CTA----------------

---T---------AAATA--
-----------T-G-TA-Tcccc---A-------------------------Acccctca
ggttggc-------------ATAGCa----------TTgtgtgagaaataaaccCAg---
---------------A-A--A-ATtg-----Ccccaac-----Tcag-T---GT--GC--
-Ta---Tcctctcaatgagagtaaggatc-------------------------------

-----C--TG-CT---Cacacctagagcaagga---------------------------
--------------------T---Ccagcgt-----------------------------
--C--TAA
CA
>COPIA-19_SB_LTR
TG----ACagggttagcctagtg------------TTA----------------------

----G-Cc----TaaGT----T---A---------TcT--Ttac----------------
----T--T--T-GCT--Gtaa----TT--T-AC-TTtgc---TGTA---A---T-TGtta
attacctaa---

------------------------T---AGgttcctT-GG-CAgc------------C-A
A-G--T--TG-AAT---a-TGG---TG--------Ccttggcaaccaagtcgctggtgga
cagcatct--
-------------------------------GTA--------G-TT----------T-GT
T-Ag--AA---GCTGtca-----GG---------------G-T-A-------TG--Ttgg

ggtgctgtctgccctct---

-------------------------------A---G--G--CTA----------------

---T---------ATATGcagcagccccctcacccttgtatctgtgtagttctggttttg

caat-------T-C-AA-T-------AcaatccaagcggcctctcttggccaA-------

--------------------GCTGCcctctggcag-TT----------------------

-----------------C--A-ATc------C-----------Ta---T---GT--TTg-

-Caa--Tc--

-----C--AG-TT---Ct--

--------------------T---G-----------------------------------

--C--CAA

CA

>COPIA-81_SB_LTR

TGTT-AGC---------------------------TAA----------------------

----A-T-----T--GA----T---Gcgtcatta-T-G--Taatctttg-----------

----T--T--T-GGT--Taaacac-TG--G-AG-TCt-----TGTA---A---T-AGcta

ga--

------------------------Ta--TGgg----G-AG-GAcgtga---------C-C

G-G--T--GG-CAT--Gc-TAG--------------ggcacgatctatcctgggttggtc

gagtttgcgtcatcccggacattgcgcatgcgacgtgaaccgcggtgaaacgggacacgt

acggtgtacggggtggcgcggtgggac----TCAG-------G-CG----------T-AT

G-A---GC----CTG--------GGa--------------C-T-G-------TG--Tca-

-------------------------------T---G--T--GTA----------------

---T---------AAATAtcaa--------------------------------------

-----------T-G-AA-Tt------Aagaccgtgaga--------------A-------

--------------------GCTGCga---------TTcctag-----------CAgcg-

---------------A-C--A-TAt------C-----------Ca---T---CTaaTC--

-Tcg--Tgtgcctacgatctcgtttgtaaactcttgccgcg-------------------

-----G--TT-AT---Cacagagacaccggcg----------------------------

--------------------T---Cgaggtccggagtccagggcatcgtctggct-----

--C--CAA

CA

>COPIA45-PTR_LTR

TGTT-AAAtgt------------------------TTA----------------------

----T-------T--GTt---T---A---------T-T--Tg------------------

----T--T--A-TTT--Ga------TT--T-AC-TC------TGTA---T---T-TGaat

aagttcctatttaccaggaaccagtcaattgtttacctattccttaggaaaatcccagct

gac---
------------------------T---AGg-----T-GA-CAg-------------T-T
-----A--GT-CATggGa-TAG----AtgctgcatA------------------------

-------------------------------TT---------T-TTc---------T-A-
C-T---TA---TCTA--------TT----------------aT-T-------TG--Ta--

-------------------------------T---T--T--CTA----------------

---T---------TTAAGttgaactcttcttaa---------------------------
-----------T-G-AA-At------Atatag--------------------A-------
--------------------GTTGCac---------TTc---------------CAcgtt
tgttgtt--------A-C--A-AAg------C-----------Tt---Tag-CT--TCac
-Tg---Tcacgttttcaatacaaaactttagctttgaattagtgcaaaacactta-----

-----G--TG-TT---

---CAA
CA
>COPIA5-ZM_LTR
TGTTaGGAaag------------------------TAA----------------------

----T-Cct---T--GA----T-------------TgG--T-------------------

----Gc-T--A-GTT--Ta------CT--T-AT-TC------TGTT---T---T-AGgaa

a---

------------------------T---AGc-----T-AG-TA-----------------

-----T--GG-TAG-----TGG----A--------Tcagtcttatatggacggatccaat

cagcaagatcaggactgatagcagtccggatta---------------------------

-------------------------------TTAC---------CT----------T-GT

A-A---TA---GCTA--------GT---------------T-T-G-------TT--Tcct

tatgaa--

-------------------------------A---G--T--CTA----------------

---T---------AAATAtatggccttagaggcta-------------------------

-----------A-G-AA-A-------A-------------------------G-------

--------------------GCAGTca---------ATaggacgc---------CAc---

---------------C-A--A-ATctg----C-----------Tg---T---GT--TC--

-C----Tctccaaaa---

-----G--AG-TT---Cgcgtgcgtgtgagtccctgggagg-------------------

--------------------T---Ttttcttatacctgaagaacttacctgggaggtagg

aactcgccggcaacctgcagggttgtctctccggcgcagttt------------C--CAA

CA

>COPIA-59_SB_LTR

TGTT-AGA---------------------------TAA----------------------

----G-Cct---T--GC---------gcc------T-T--Agactta-------------

----G--C--T-GCA--Tg------TT--T-TT-TCa-----TTCA---G---T-TG---

------------------------TgcaTGcgcac-G-AG-CA--------------C-T

G-Ac-----T-TGT--Gg-TTG--------------ccgtgcgcctaggagaactggccg

agattagctcgccatgactcgcgacatgcagccacggctccaacgaaagtggcgcgtggg

gatggacgcgcgcaatgcgctgc--------GTGG-------G-CGccg-------T-GG

------------CTGg-------GT---------------G-T-G-------TT--Tg--

-------------------------------T---T--T--CTA----------------

---T---------ATTTAccgcaatcaaaaac----------------------------

-----------A-G-AA-A-------A-------------------------C-------

--------------------GCTGCttg--------TTgttcg-----------CAgca-

---------------C-C--A-AAa------C-----------Tcga-Tc--GTg-TC--

-Caag-Tgttcgcgtgtgtttctctcgccgccgctcgtgcgctcaccgcg----------

-----G--CG-TT---Caccgccgttcggt------------------------------

--------------------T---T-----------------------------------

--C--CAA

CA

>COPIA-131_SB_LTR

TGTT-AGC---------------------------TAA----------------------

----A-C-----Ta-GA----T---AgcaaaccgaT-G--Taatctttg-----------

----T--T--T-CAA--Gtattg--TT--T-AGaTCa-----TGCA---T---T-AGtcg

------------------------T---AGtgggc-T-AG-GAcatga---------T-C

G-G--T--GG-CGT--Gc-TGG-------------A------------------------

-------------------------------TC---------G-TG------------AT

C-T---AA---GCCGa-------GTcgg------------T-C-T-------TG--T---

-------------------------------A---T--C--C--ggcgccagaacgcgtg

ctgcgcacgcggcgtgctccgcagtgaaacagagtcgcgcacgaccgtgatctggcgcgg

agagtccttggcacgaacgccaagattgtatctgcatcatgtg-----------------

---T---------ATATA--

------------cC-GA-A-------A-------------------------G-------

--------------------GCTGCaatg-------TTggg-------------CAgcg-

---------------C-Ct-A-AT-------Taa---------T----T---CTa-TC--

-T----T---

-----G--AG-TT---Catc--

--------------------T---Tctagctctcatcgtggtcaccgccgtcgagcgtcg

ccgggttcgcgcacaggaacgctattcggtt-----------------------C--CAA

CA

>CPSC4B_LTR

TGTT-AGTaa-------------------------TAA----------------------

----G-Tc----Ta-GC------------------T-G--Ctcagagtagttagtttccc
tctgT--T--T-TTAc-G-------TT--G-AG-TCt-----TGCAc--A---TgATctg
ttcatcctgtttgtgtgcatagtgggcttgacgcacgctgcgttcgtgcggagtccgtgg
tgtcgtgcgtggtgtgctccagttcgtgggatgacgcga---------------------
----------------------------AG------T-GG-CA-----------------
-----A--GG-AGT--G--TGG---CAcgcaca--A------------------------

-------------------------------TTAG-------G-AGgccac-----T-AT
G-Gcc-AC---GTTTaac-----TT---------------T-C-T-------TG--Taat

-------------------------------T---T--G--CTA----------------

---T---------TTAAGgttaagc-----------------------------------
-----------T-C-TA-AtccagaaA-------------------------A-------
--------------------GCTGCcag--------TT----------------------
--------------------A-ATg------Cag--------------------------


```
------------------------------------------------------------
---------------------------------------------------------CGC
CA
>COPIA11-ZM_LTR
TGTA-GAAag-------------------------TAA----------------------
------------------------------------------------------------
------------------------------------------------------------
------------------------------------------------------------
------------------------------------------------------------
------------------------------------------------------------
------------------------------------------------------------
------------------------------------------------------------
----T-Cct---Tg-GA----T---Gcca------T-G--T-------------------
----T--T--A-GCA--G-------TT--T-A--TCt-----TG-A---T---T-AGctt
aaat--------------------------------------------------------
------------------------------------------------------------
------------------------T---AG------G-TG-TAtga-----------T-T
G-G--A--AT-AAG--G--CTG----A--------Ttgagagccgttat-----------
------------------------------------------------------------
-------------------------------TTAG-------T-TTcct-------T-AT
T-AcagTA---GTTCct------TT---------------C-C-A-------TG--Taat
------------------------------------------------------------
-------------------------------A---G--T--CTA----------------
------------------------------------------------------------
------------------------------------------------------------
---T---------ATAAAccaagagatatcaa----------------------------
-----------T-G-AA-A-------A-------------------------A-------
--------------------GCAGTcac--------TCgacag-----------CAc---
---------------C-A--A-AAa------Taaga-------Tcg--T---GT--CCa-
```

-C----T---

-----G--TG-TT---Tcgcgtgag-----------------------------------
--------------------T---Ttctgagtgttgtgttggccgtgatctcgccggcat
cctgtgcggtgtcactccggcgaggttctagtt---------------------C--TAA
CA
>COPIA-31_SB_LTR
TGTT-AGAtactcc---------------------TAA----------------------

----G-------T--GC----T---G----------------------------------
-----------gGTA--G-------AT--T-AG-GAa-----TGCAc--G---T-TTttc
cacttcttgcatgcgcgcagacaccgggctgtggtgatcgcgcgctcaacttagcgg---

------------------------T---AG------T-TG-CAtg------------C-A
-----T--GC-CGC--Ca-TGG--------------------------------------

--C-CG----------G-AT
G-A---AC---TC-G--------GT---------------G-C-G-------TG--Ggga

-------------------------------T---G--G-----acgcgcacaaggatcg
ggtcatgcgcgccatggccggtatgccctctctgtttttccca-----------------

---T---------AAAAGg---

-----------A-G-AA-At------Atacaccggaaa--------------Ac------

--------------------GCTGCgcag-------TTtgg-------------CAgc--

---------------A-C--A-AAat-----C-----------T------------TC--

-C----Tcgcactctgcgtcgctcagtttcttgcatccgcgtttctgctcgccgccggcg

tccgctgcgcgcacgccgccg---------------------------------------

-----G--CG-TT---Cgccg---------------------------------------

--------------------T---Cgaccacgt---------------------------

--C--TGA

CA

>COPIA-38_SB_LTR

TGTA-AGAta-------------------------TAA----------------------

----T-C-----T--GC------------------T-G--Tccc----------------

----Tc-T--T-GTA--C-------TT--T-TG-TCc-----TGCA---T---T-CGgca

ggttcaagtggtcagctcttggctgccactgtagaagtatggcaataggccatttagtct

tccatagttggtagaagtacaccagctagag-----------------------------

------------------------T---AG------T-GG-G---------------C-T

G-G--T--TT-AGG--C--TGG---TA--------Caagagtaggaattggctgagataa

g---

-------------------------------CCA----------TG----------T-AG

T-G---TA---GTTG--------TTgga------------G-T-Gg------TG--Tagt

caccatccaagtagtgtaccagcaggtacatagct-------------------------

-------------------------------T---G--T--GTA----------------

---T---------ATATAccctacctattgcaa---------------------------
-----------T-G-GA-A-------A-------------------------Aac-----
--------------------TCAGTtca--------TTgc--------------CA----
---------------CtC--A-TTcag----T-----------Tc---TccaGT--TT--
-Tcag-Ttcaagtctgaaagctgagtgtgtgtgtgctgcgtgagtgagtgagggagctca
gggctcttcctcttccttggagcaggggtgagggagagggaaggtgagcagggc------
-----C--TG-CT---Cacctggtgc----------------------------------
--------------------T---Tgtgtgcaggtg------------------------
--C--CAA
CA
>COPIA-21_SB_LTR
TGTT-AGC---------------------------TAA----------------------

----A-T-----T--GT----T---Gcaatatc--T-T--Tgtaagctttg---------
----T--T--T-TCA--G-------TTg-G-AG-TCt-----AGCA---T---C-AGggt
agagtcagtcgg--

------------------------Tc--AGgg----G-AG-AAggga----------T-C
A-G--A--GT-CAT--Gc-CCG----A--------Tcacgatcccggtctagtttggtca
agccacgccgtggcgtgcacggcgcatgaggcgcacgttccgcagcgaaacggga-----
-------------------------------CT---------G-CG--------------

C-A---CC---TGTGc-------GTacg------------G-C-A-------TG--Ggga
gtctgtaaactcg---
-------------------------------A---Gc-T--CTA----------------

---T---------ATAAAca--
-----------A-G-AA-T-------Agagggaccgtaaa------------G-------
--------------------GCTGCga---------TTtg--------------CAgcga
---------------C-C--A-TA-------Ca----------Tc---Ta--CT--TC--
-Tc---Tagggttttgtatctgcttgagcttcggggttggcgtggtcaccgtggtcacca
cgcgccgccgttcgacgtcgttccttggttgatct-------------------------
-----G--TG-CT---Tgcgg---------------------------------------
--------------------T---T-----------------------------------
--C--CAA
CA
>COPIA-27_SB_LTR
TGTT-GAAcccagagtctg----------------TAA----------------------

----T-Cg----T--GTc---A---G----------------------------------
------------TCA--T-------TT----AG-GTg-----TGCA---T---T-AG---

------------------------G---TG------T-TA-CAtttt----------T-A

T-G--TtcAG-CAT--Gt-TTG---TA--------A------------------------

-------------------------------TTA--------G-CG----------T-AG
A-A---TC----CTGg-------GT---------------G-C-G-------TG--Gcga

-------------------------------T---T--G--C--acacgcactcaccagg
accgcgtcttcagggccatctccgccatggctggcacgccgtgcatgtcgcacgggcacc
gaaaatgacggcgcaccgtgcagcgcccaaccgtggcggagggaggcgatccaggcc---
---T---------ACAAGgccgagt-----------------------------------
-----------T-G-AA-Tgc-----Acaaacaa------------------A-------
--------------------GCTGCgcg--------TTcg--------------CAgtaa
---------------A-CtgA-AAcggtg--C-----------Tc---T---CTc-TC--
-Tcg--Tgtatcgctcgctcgccgccgtagtagttttagttgacgcggtcaccactcgcc
g---
-----G--TG-TT---Ccgcctcgccgggctcctcgccggagctgcgg------------
--------------------T---C-----------------------------------
--C--CAA
CA
>COPIA14-PTR_LTR
TGTT-GAAgtttggtacc-----------------TAA----------------------

----G-C-----Ta-GA----AcagG---------A-T--Tac-----------------
----T--T--A-GCA--G-------TA--A-TC-TAc-----TGCA---T---G-ATtat

ctagttgttagctgagattactatctagattaggatatggttttaattcttttgttaagt

ttatctaggattaaactaacttgtaaacatctccttaaaa--------------------

------------------------T---AA--------AG-GA-----------------

G-A--A--GT-TGG--C--TGG---TAcgtt----T------------------------

-------------------------------CTA--------G-AGt---------T-TG

T-T---TC---TCTTt-------GTaa-------------T-T-C-------TG--Tttt

ctt---

-------------------------------T---T--C--CTA----------------

---T---------AAATAaaagggcatgggatggt-------------------------

-----------A-G-TA-Acc-----A-------------------------Actactca

at------------------TCTGCtc---------TTtgttctta--------TActt-

---------------A-A--A-TActaagttC-----------Tg---T---GT--TC--

-T----Ttcttttgttcttttccatcgtggacttagttctttgttcttttctcttttatc

agcaaacataagg---

-----C--AG-TT---Ctttatat------------------------------------

--------------------T---T-----------------------------------

--C--TA-

CA

>COPIA-29_SB_LTR

TGTT-GGAgcagagctcgtcg--------------TAA----------------------

----T-Cg----T--GTc---A---A---------A-G--Tgcatatt------------
----TccT--T-TGA--T-------TA--G-AT-GCat----TGTA---A---T-AGttc
aattaagttgtgaacatt--

------------------------T---TGtt----T-AG-CAc-------------G-T
A-G--A--AT-CACg-Gg-TTG--------------------------------------

---------------------------------AG-------G-CG----------G-AG
-------A---CTTGcac-----GTc--------------T-C-Gacgc---TG--Tgac
cg--
-------------------------------T---G--C--C--cagataggttcacccc
gccacggcgcgcatgccgtgcttgtcgcgtggacgtccgcaaatgacggcgtatctcgca
gctgccaaccgtggcggggatctcggatcgagccata-----------------------
---T---------ATATAtcaagtttgt--------------------------------
-----------T-G-CA-Tctggta-A-------------------------G-------
--------------------GTTGCgcca-------ATcgcggc----------TAaa--
---------------C-Ct-A-ATcg-----C-----------Tg---T---GTc-TCg-
-Tg---Ttctctctcgtcctc---------------------------------------

-----G--TG-TT---Cgtg--
--------------------T---Tcctgtggtgaccgcgcaccgagcatcgacggtgcc
ggcacgttccaagctccggcgagccgccggac----------------------C--TTA
CA
>COPIA-85_SB_LTR
TGTT-AGC---------------------------TAA----------------------

----A-T-----T--GA----T---Gcagagtct-T-G--Taatctttc-----------

----G--T--T-TCA--G-------TT--G-AG-TTa-----TGTA---A---T-AGtcg

g---

------------------------Ta--AGgggct-G-TG-CAtga-----------T-T

G-G--T--GT-TGT--Gc-CTG----A--------Tcatgacacagcttagaagagtcgg

tcgtgtccgcgtcatggtgtgccgagcgcatgagacgtgcactg----------------

-------------------------------CTAGgaaacg-G-TG----------T-AG

C-G---TA---CTCG--------ATatg------------G-C-A-------TG--Gcgc

gggacgggctcagttgggcctgctg-----------------------------------

-------------------------------T---Gt-T--CTA----------------

---T---------ATAAAca--

-----------T-G-AA-T-------Aaagaccgagg---------------G-------

--------------------GCTGCaatg-------TTgg--------------CAgcgc

cg-------------C-A--A-TAt------C-----------T----T---GTc-TT--

-C----Tcacgtgaacttgttttcacctaatctagatctcgccgcggtcaccacggtggt

gtgcagcgacgttcca--

-----G--AG-TT---Cctgatga------------------------------------

--------------------T---Tcttggct----------------------------

--C--CAA

CA

>COPIA-25_SB_LTR

TGTTgGGC---------------------------TAA----------------------

----T-Cc----A--GCg---T---G---------T-G--C-------------------
----G--C--A-GGT--Ggc-----AT--G-TG-GCcc----AGCTg--G---A-AGtgc
ccggtcagcagagctaggcgggagattcagaatttggatggaagttcaggggtgcgcggt
gagcgcgcccacgacatcgctggtcaacag------------------------------
------------------------C---AG------T-TG-CA---------------aA
G-T--A--GG-CGT--C--TTG---TAtccatg--T------------------------

-------------------------------TT---------G-TG----------T-GG
T-T---TT---TTCAt-------GT---------------G-T-G-------TG--T---

-------------------------------A---T--G--C--aatgtattggctggga
tataaagccaaggtgtagcactacttagcggttgcttcgtca------------------

---T---------ATGTAatcgtgtgttgtatcgtgaactattctgag------------
-----------T-G-AA-A-------Aagggcactgtatccctgtcgac---A-------
--------------------GTTGCcagg-------TTcgggg-----------TAc---
---------------A-C--A----------C-----------Tg---T---GT--TC--
-T----Tgagc---

-----G--TG-TT---Catccattgatttcgcgttttctttgtgtttccactgaaatcac
accgtggtctcaatcctagaT---T-----------------------------------
--C--CA-

CA

>COPIA9-PTR_LTR

TGTT-GAA---------------------------TTA----------------------

----T-Cat---Ta-GT------------------T-T--Taaagtttccctg-------

----T--T--T-TAA--G-------CC--T-AT-TTc-----TGTT---A---T-GTtga

ataattcctccttgtcttgaggaacaagattgtttttctgttacagtagccatgtacca

cga---

------------------------T---TA------T-AG-GAt-------------T-T

T-A--A--AT-CGTt-Gg-TTG--------------------------------------

-------------------------------TTAT-------T-CT----------T-TT

C-T---TC---TTCCa-------GT---------------G-T-A---------------

-------------------------------A---G--G--CTA----------------

---T---------TTAA---

---------------TA-Gcc-----Acatc---------------------A-------

--------------------GTTGCttaa-------TT----------------------

-----------------At-A-TTgaa----C-----------T----T---CT--TC--

-Ca---Tctcttgtgtaatcaaacctgtcaaagctctcaaagctcttta-----------

-----C--TT-TT---Cttta---------------------------------------
--------------------T---T-----------------------------------
--C--TA-
CA
>COPIA-138_SB_LTR
TGTT-AATag-------------------------TAA----------------------

----G-Ccg---T--GA---------gc-------T-G--Tac-----------------
----T--T--T-CCT--G-------TT--T-TG-TAcc----TTC-----c--T-ATtct
actgctagtgctaacggcatggcccagggagttgggatggcaacccccaatcacgagcat
gccttataggctcttgccttttata-----------------------------------
------------------------T---TG------T-TG-C---------------C-T
A-G--T--TT-AGT--Ga-TTG----G--------C------------------------

-------------------------------TTA--------T-TGcct-------T-AG
--A---TA---GCT------------------------------A-------TG--Ta--

-------------------------------A---TcaG--CTA----------------

---T---------ATATAgtgaac------------------------------------
-----------T-G-TA-Ttc-----Agaggaat------------------A-------
--------------------GCTGCctcac------TGcac-------------CA----

---------------C-A--A-TAc------C-----------Tg---T---GTctTC--

-T----Tctgtgtgtgtgttgatcg-----------------------------------

-----G--TG-CT---

--C--TAA

CA

>COPIA-9_SB_LTR

TGTA-AGAca-------------------------TAA----------------------

----T-C-----T--GC----T---Gccccc----T-T--Tctc----------------

----T--C--T-TCT--T-------TC----TG-TCc-----AGGAc--A---G-AGaca

tggtagtggatcagatcatctcatggctggtcaagtctctagccctgtagagtataaggc

aataggccatacttgtactagtagaagtacaccagcagtatggctggtactagagtag--

------------------------T---TG------T-TG-G---------------C-T

G-A--T--TT-CAG--Cc-TTG---TAc-------C------------------------

-------------------------------CTAG-------G-TG----------T-AT

---g--TA---GTTG--------GTaga------------G-C---------TG--Tacc

ttagaaggtacatgtaa---

-------------------------------T---Gg-T--GTA----------------

---T---------ATAAAccagtccaa---------------------------------

-----------T-GcAA-Tgg-----Aacc----------------------A-------

--------------------GCAGCaag--------TTcattgc----------CA----

---------------CtC--A-TT-------Cag---------T----TccaGT--TT--

-Tag--Ttcaagtctgaaactgtgtgtgtgctgtgtgtgtgtgcagtagtagctcagggc

ttttcttctaccttggagcaggggagagggagaggggaggagagcaggtg----------

-----C--TG-CT---Cacctcggtgcagga-----------------------------

--------------------T---Tgctgtg-----------------------------

--C--CAA

CA

>COPIA-112_SB_LTR

TGTTtGAA---------------------------TAA----------------------

----GtCct---T--GA------------------------gca----------------

----T--C--T--AA--T-------TA--T-AG-AA------TGTA---G---A-GGcgc

ctaacgccattagaggcaggccacaga---------------------------------

------------------------T---TG-------cGG-CAtggcc---------C-A

G-G--T--GGtTGG--Ga-TGG---CA--------Accactgatcacgagtatgccggga

-------------------------------TTAG-------C-AGg---------TcAT

T-T---TA---TTTCctt-----GTaacaca---------T-C-A-------TG--Tat-

-------------------------------T---T--C--CTT----------------

---T---------ATATAg---

-----------T-G-AA-T-------Acgatcaatggaa-------------Act-----

--------------------GCTGTcgc--------TTtacag-----------CAa---

---------------C-C--A-ACtct----C-----------Tg---T---GT--TCg-

-Tgtg-T---

-----G--TG-TT---Catcctcatcggat------------------------------

--------------------T---T-----------------------------------

--G--TGT

CA

>CPSC5_LTR

TGTT-GGAttg------------------------TAA----------------------

----T-Cc----T--GA---------gca------T-T--Tatattctg-----------

----T--T--T-TGT--T-------TT--G-TT-GCa-----TGTA---G---A-GT---

------------------------Tc--AG------T-TG-GAgt------------C-T

G-A--A--TT-CTG--Gc-CGG--------------------------------------

--------------------------------TA--------G-AA----------T-AG

C-At--TT---TCTGt-------TTta-------------G-C-A-------TA--T---

-------------------------------T---T--G--C--ttcattctttagctgc

gatgactcggctcggatcgtgggatggcgcggtcagtagttgatccgagattgtcgtgat

tctgttatgcatgcaacaaactccgtgtggagatgaa-----------------------

---T---------AAATAgaagagaaaac-------------------------------

-----------T-G-GA-A-------A-------------------------A-------

--------------------GCTGCacggca-----TTacg-------------CAg---

---------------C-G--A-AAa------C-----------Tc---T---GT--TCg-

-Tcag-Ttgttctgttctgctctgttttactgaaaaattgttcagttaactgtagctgca

cctgcaagcgtcagtgtgt---

-----G--TG-TT---Ctgtgagattgagagccagttcgcgtgagtt-------------

--------------------T---T-----------------------------------

--C--TGA

CA

>COPIA26-PTR_LTR

TGTT-GACagttgcc--------------------TCA----------------------

----T-T-----T--GC----T---G----------------------------------

-------C--T-GAA--G-------AA--G-AG-GTc-----TTC----G---A-TGctg

tgaagagtcagtcaagattggttccaga--------------------------------

------------------------T---TGt-----T-AG-GA-----------------

-----T--GT-TAG-----TTG----A--------Aaataaagctgtcatagatcagctc

tttctctgttt---

-------------------------------TTA--------T-CT----------T-CT

C-Aa--TA---TTT---------TTcggaa----------T-T-C-------TG--Ttat

caaac---

-------------------------------Tc--T--T--CTA----------------

---T---------AAAAGagtgattta---------------------------------

-----------T-C-AA-T-------A-------------------------Aagaaaat

--------------------ACTTCta---------TTca--------------CA----

---------------A-C--A-TTta-----Ta----------Tc---T---TTa-TC--

-T----Tattgct---

-----G--AG-TT---Cttgcctg------------------------------------

--------------------T---Tcttatacaatttgtttctgtcataaa---------

--C--TA-

CA

>COPIA-28_SB_LTR

TGTT-AGT---------------------------TAA----------------------

----T-C-----T--GT---

----------T-GCAccT-------AA--G-AG-TCc-----TGTA---A---T-CTttc
at--

------------------------Tc--AGt-----T-AG-CGt-------------T-T
G-G--A--GT-CGT--Gt-AAG--------------------------------------

---------------------------------AGg------G-AGac--------T-GG
-----------GTTG--------GT---------------G-TcG-------TG--Cccg
accctgctcacctcggagtc--
-------------------------------T---G--T--C--gtgaacacgccatggc
aagttaggcgcacggggcgtgcgcaccggatatcggcgtcgcgcacccactcgtcatggc
gtgggagtcttgtaaaacaagcac------------------------------------
---T---------ATAAAtaaagagg----------------------------------
-----------A-G-AA-Gggtcgt-A-------------------------Aaac----
--------------------GCTGCggga-------TTg---------------CAg---
---------------C-A--AcAA-------C-----------T----T---GTtgTC--
-Ta---Ttgcattcacttgatttcagtttacatctactcgccggttcgtggtcaccacga
cgccggacatagtcgacgttcctgcgttccggc---------------------------
-----G--TG-AT---Ctcgacgttcctg-------------------------------
--------------------T---T-----------------------------------
--C--CAA
CA
>COPIA-57_SB_LTR
TGTT-AGCcta------------------------TAA----------------------

----G-Tgt---T--GC----A---A---------TcG--Aac-----------------
----T--C--T-GTAa-Tc------AT--G-TG-TT------AG-A---T---C-AGggt
gtcatgctagaggaatcctagtatggt---------------------------------

------------------------T---AGag----T-TA-GAtgtag---------C-C
A-A--A--GA-CAT--Gc-TTGgg-TAcgtcaag-C------------------------

-------------------------------TT---------G-AG----------T-CG
G-Ag--TC---TGTAtct-----GTcacgtcgtg------G-C-G-------TG--Tcag
gcgcatgtaacagggatcgcaaggaaatagtgtcctgttcctatgcgacacggcgtggga
gcctca-------------------------T---G--G--CTA----------------

---T---------ATATA--
-----------T-C-AA-At------Aaagatatcatgagg-----------G-------
--------------------GCTGCgg---------TTagg-------------CAgtaa
---------------A-C--A-TA-------T-----------Ta---C---GT--TTg-
-Cc---T---

-----G--AG-TT---Tcga--
--------------------T---Tctctagtttcaatctgttatgtggtgatcgcagtc
tccacgtcgccagcgagaacgacattctacgacgtttcagcgtcaatctgat--C--CAA
CA
>SHACOP4_LTR_MT
TGTTgAGAcattgagatttcagatgtacacaatagTAT----------------------

----T-Ttg---T--GA----T---A---------T-T--Ttatg---------------

----T--T--T-TCT--T-------TT--A-TT-TTaa----TGTA---G---T-AGttg

aagttacctctgcagaagtatggtgacccatac---------------------------

------------------------T---TG------T-TA-GAgtgtttaagac---T-T

-----T--GT-AAT--Ct-TGG--------------------------------------

-------------------------------TTAG-------G-TGacagac----T-AC

T-A---T----GCT---------GTc--------------C-T-Gc------TG--Ttca

ctttttctgttt--

-------------------------------T---T--T--CTA----------------

---T---------ATAT---

-----------T-G-TA-Tcag----A-------------------------A-------

--------------------ACTTTa----------TTttgatg----------TAg---

---------------A-AtgA-AA-------T-----------Ta---T---CT--TC--

------a-AG-TT---Tctgaaataacttttag---------------------------

--------------------T---T-----------------------------------

--C--TTA

CA

>ATCOPIA65_LTR

```
TGTT-GGAg--------------------------TAA----------------------
------------------------------------------------------------
------------------------------------------------------------
------------------------------------------------------------
------------------------------------------------------------
------------------------------------------------------------
------------------------------------------------------------
------------------------------------------------------------
----G-Ct----T--GA----A---A---------Ta-----------------------
-----a-C--T-TAA--Taa-----AT--A-AG-TT------ATTAa--A---T-ATtac
aaaggat-----------------------------------------------------
------------------------------------------------------------
------------------------Ta--TG------T-TA-AAtctc----------T-A
G-G--A--GT-TAT--C--TAGa--TAtttaa---A------------------------
------------------------------------------------------------
-------------------------------TTAG-------G-AGtta-------T-CT
A-A---T----GTTA--------ATccta-----------G-T-A-------TGa-Ttag
gtctaatta---------------------------------------------------
-------------------------------T---Tc-T--CTA----------------
------------------------------------------------------------
------------------------------------------------------------
---T---------ATATA------------------------------------------
-----------T-G-AG-Tccc----A-------------------------A-------
--------------------GGTGTgg---------TTgac-------------CAtatg
agttttaaagagattA-Ag-A-TT-------Tagaga------T----T---GT--TTag
gT----Tttgagttattttcctaaactataaagagagttattcttaaatcttt-------
------------------------------------------------------------
-----G--AG-TT---Cttaatc-------------------------------------
--------------------T---Tctttgaaaa--------------------------
```

--C--TA-
GA
>COPIA-51_SB_LTR
TGTG-GGAg--------------------------TAAgcttgctgcagggatgacgtgg
aggtgatgaagctgagctggcaaagcgggaacgtccatcagattcaaatttgaatgggag
cgtggacggttggcctcatcagacgtggtgaccgcgcccacggcgcgtcctggagcaagc
agttacatctacctaca---

----T-T-----T--GT----A-------------TcT--T-------------------
----G--T--T-TAT--T-------TA--A-AT-TT------TCTA---G---T-GTgta
accatgtccttttac---

------------------------T---AC------T-GG-TA--------------T-A
A-G--T--AG-TGT--Ga-TG----TA--------A------------------------

-------------------------------GTGGa------G-TG----------T-GT
T-A---TC---GCGGt-------GT---------------G-T-Tggt----TG--Taaa
cacc--
-------------------------------T---G--T--GTA----------------

---C---------TTGAActtgtgagagg-------------------------------
-----------T-G-AA-A-------A-------------------------Ggggcgcc
gtcacccaggcggttcctgtGCTGTtcatg------TG----------------------
-----------------C--A-TTgat----Cgcgt-------TccagT---GT--TC--
-TaggcTagccaaatccatcaaattatcgctgtgatt-----------------------

-----G--TG-TT---Cctgat--------------------------------------
--------------------T---T-----------------------------------
---CAA
CA
>COPIA18-PTR_LTR
TGAA-GGAgtcttctcg------------------TAA----------------------

----A-C-----Ta---------------------T-A--T-------------------
----T--C--T-GGT--Tgt-----TA--G-AG-TT------TGT--------T-AGaat
aaataacaaactagaagggagagtcctagttttacagggcattgcaaat-----------

------------------------Tac-AGt-----T-AG-CAacc-----------T-C
T-C--T--GT-TAGa-Gt-TTG--------------------------------------

-------------------------------TTAC-------T-TT----------T-CT
G-T---TAt--TCTG--------GTa--------------T-C-A-------TG--Tatt
ag--
-------------------------------A---G--G--CTA----------------

---T---------ATAAAgccaagagttcttgc---------------------------
-----------T-G-AA-T-------Aatac---------------------A-------

--------------------ATTTCca---------TTtatcc-----------CAgttc
---------------A-CgtA-TA-------C-----------T----T---CT--TC--

----------aAT---Catccataa-----------------------------------
--------------------T---C-----------------------------------
--C--TAA
CA
>COPIA23-ZM_LTR
TGTT-GCAttgatctctttg---------------TAA----------------------

----T-Cg----T--GTca--A---A---------T-G--Aacacgta------------
----T--C--T-CAT--T-------TT--G-AT-GCa-----TGTAg--G---T-AGttt
gttt--

------------------------T---AG------T-AA-CA-----------------
-----A--AT-CAT--Gc-ATA--------------------------------------

-------------------------------TTTG-------G-CG----------T-AG
A-A---TC---GCGG--------GTga-------------G-C---------TG--Gaga
c---
-------------------------------T---G--C--C--caatctcacgccgtga
ccgcatttttgtcaggaaagaaccaccgcagcagatttgccgtgcatgtcgcactgggcg

ttccggaaataacggcgcaccgtgcagcgatctgttgcggtggggaacacgggggaatcc

ctcT---------ATAAA--

-----------A-G-GA-Ac------A-------------------------Aggattgc

att-----------------GGTGTtga--------TTgcacgcatttg-----CAg---

---------------A-G--A-AAt------C-----------Tcg--T---GT--CC--

-Tc---Tcttgtactctcgttcctaggtctgtagcgtccgcggtcaccaccggccggcgt

cgcggactcgccggcg--

-----T--CG-AT---Cttcgacaggtgg-------------------------------

--------------------T---T-----------------------------------

--C--CAA

CA

>COPIA-92_SB_LTR

TGTT-AGC---------------------------TAA----------------------

----A-T-----T--GA----T---A---------------gcaca--------------

----T--C--T--AT--G-------TA--A-TC-TT------TGTT---A---T-GGtaa

ggatcatgcatgagtcgggtaggttg----------------------------------

------------------------T---TG------G-GG-GAtaagcctga-----T-C

G-G--T--GG-CGT--Gc-TAG----A--------Tcaggacttaacccggagtcggtcc

atgctcgcgccagagtacgcgctgcgcacgtggcgtgacccgcagtgcaacggtgtcacg

cacggttcgtga-------------------TCTG-------G-CGcggggagactT-GG

C-Aa--TCaagGCCAag------GT---------------G-T-A-------TC--Tgca

tc--
-------------------------------A---Ta-T--GTA----------------

---T---------AAATAgca---------------------------------------
-----------T-G-AA-T-------Aaaggaccgtga--------------A-------
--------------------GCTGCgacaa------TTtgg-------------CAgcg-
---------------C-C--A-TAatc----C-----------Cg---T---GT--TC--
-Ttc--T---

-----C--TG-TT---Ttg---
--------------------T---Ttcatctacattcgctctcgccgcggtcaccacgaa
cacgccggcgtcgagactacggaccaagggcatcgctcggtt------------C--CAA
CA
>COPIA4-SB_LTR
TG------------------------------------actgtcaaggtgacttggtgca
taggaaaagtacttcaggcatcatattctaccttggatcaaatttggtgacctgggcttc
acagaaacaaagggtcgtggctctatcttcttgtgaagctgagtacatagctgcagcatt
gggagcctgtcagggagtgtggctcagtcggctgatagaggagaagttcagaaattcaga
cttctcatcgataaccagtctgctatcgagctagccaagaacccagtgtttcatgatcgc
agtaagcacattgatacacggtatcattacattcgtgactgcattgagaagaacgtcctg
gatgtggaccatgtaagaacagacgagcaggttgctgacattctgacgaagcccttgggg
agaatgaagtttgcggaattcagagtcaagctcggcgtcgttctagttcgtcaggattag
gggg--------T--GA----T---T---------T-G--Tta-----------------
----G--C--T-TTAgcT-------TT--G-TG-TTtca---TTCAt--G---T-AA---

------------------------Tcc-TG------Tc-G-AAgttt----------T-A
T-G--T--AA-CGTa-Gt-TGG--------------------------------------

-------------------------------TTAG-------G-CGcg--------T-AG
A-Ta--TC---GCCG--------GTg--------------G-T-Tcgaaga-TG--Tgca
agaaccacgcggtgaccgcgcactgcattg------------------------------
-------------------------------T---G--T--C--cccgtcgcgacgtcgt
cttgcatgtcccagcggcgttccggaaaatcggaggatcgttggaggcgtcgtggcggcg
gggtgtaaggcctcaggcca--
---T---------ATATAaacca-------------------------------------
-----------A-C-AA-Ag------Attgcattggcg--------------A-------
--------------------GGTGCccgca------TTtgg-------------CA----
---------------C-G--A-AAt------C-----------Tcgg-T---GTc-TTg-
-T----Tgctttccatattttctgttcgcgcacagagctcgccgtagggttttagccgcc
gc--
-----G--TG-TT---Caccacctgccggtgacccgccgccgccagacttgttcatcacg
agtttgg-------------T---T-----------------------------------
--C--CAA
CA
>COPIA-30_SB_LTR
TGTT-AG----------------------------TAA----------------------

----T-Cc----T--TT----T---G---------TaG--Tctgttacatagcatagcat
----G--T--T-TGT--T-------AT--TcTG-TTc-----TGCA---T------Gcac
acgagggccggct---

```
------------------------------------------------------------
------------------------Tg--TG------G-TG-CCcgtgtgcg------T-A
G-G--A--GG-TAG-----TGG----A--------Cgagattagctcgccatgaacctga
gcatgcaagccatggccggctgttacggcgcatggggatggacgtgcgcatc--------
-------------------------------CT---------G-CG----------T---
G-A---TC---G-TGcggcc---GTg--------------G-C-T-------TG--Tgct
gctctgctttgtaa----------------------------------------------
-------------------------------T---T--C--CAA----------------
------------------------------------------------------------
------------------------------------------------------------
---T---------AAATAagagtcgaatacatt---------------------------
-----------A-G-AA-A-------A-------------------------C-------
--------------------GCTGCcgcga------TTtgcagca---------CAa---
---------------A-A--A-AAct-----C-----------Tg---T---GT--TC--
-Cag--Tgttcgtgt---------------------------------------------
------------------------------------------------------------
-----G--TG-TT---Cttg----------------------------------------
--------------------T---Tccctcacctctgctcgtgtcctcgccgccggtgtg
caccgacgtccggtga--------------------------------------C--TGA
CA
>COPIA-105_SB_LTR
TGTT-AGC---------------------------TAA----------------------
------------------------------------------------------------
------------------------------------------------------------
------------------------------------------------------------
------------------------------------------------------------
------------------------------------------------------------
------------------------------------------------------------
------------------------------------------------------------
```

----A-C-----T--GT----T---Gcgtca----T-G--Taa-----------------
----T--T--T-GAA--Tag-----TT--A-AG-TTca----TTGAgtcG---T-AG---

------------------------Tc--AGggt---T-AG-CA-----------------
G-G--T--AG-CGG--G--TGGc--CA--------Aaggcacgcttggtccacgacacag
c---
-------------------------------CTAG-------G-AGgtc-------G-AG
A-G---TC---CGCGcc------AT---------------G-CcA-------TG--Ta--

-------------------------------T---T--G--C--agcgcgtagcgtacgt
tgcgaggaaacgatgtatcgcgcgataagcagtatggcgcggggagactcggagcctgtt
ccgagatctctaggagtccctaa-------------------------------------
---T---------AAAA---
-----------G-G-AA-Ag------Acatgatgctttgcgg----------G-------
--------------------GTTGCg----------ATacgg------------CAg---
---------------C-C--A-AAtat----C-----------Tcg--T---GT--GT--
-Tgg--Ttgttttgtgtttccatctatctcgttttcgctctcaccgcggtgatcgcgatc
accgtcacaccaccattggcgcagcac---------------------------------
-----G--AG-TT---Ccagggcatcgcctgg----------------------------
--------------------T---T-----------------------------------
--C--TAA
CA
>COPIA-107_SB_LTR
TGTT-GATttgcatcctg-----------------TAA----------------------

----T-Cg----T--GTca--T---Act-------T-G--Ta------------------

----T--C--T-GCA--T-------CT--T-AG-T-------TGCA---T---T-AGct-

------------------------T---AGt-----T-TGtCA-----------------

---------G-CAT--G--TT----TA--------A------------------------

--------------------------------TAT-------G-CG----------T-AG

A-A---T----TCTGg-------GT---------------G-TcA-------TG--Gtga

-------------------------------T---T--G--C--acacgtgagcacgccg

gaaccgcagattgtaatcctccccgtcatagctggtgggccgtgcatgtcgcatgggtga

cccaaatgaggcacaccgtgcagccgctatttgtgacggggaagaggcc-----------

---T---------ATATAa---

-----------G-G-AA-At------Aagattgcatcgtttaa---------G-------

--------------------GCTGCgca--------TTg---------------CAgttg

aa-------------A-C--A-AAtc-----C-----------C--------GTc-TC--

-Tc---Tcgcgtttctctgtttccatagtttgttagggtttaactcgccgcggtcaccac

cagccggtgccgcgacgtcgccgggctcaatccgc-------------------------

-----G--AG-CT---Cgg---

--------------------T---T-----------------------------------

--C--CAA

CA

>COPIA-74_SB_LTR

TGTT-AGC---------------------------TAA----------------------

----A-Taga--T--GCac--A---A---------T-G--Taatcttcg-----------

----T--T--T-CCT--Tgagtca-TT--G-AG-TCa-----TGTA---A---T-AGtcg

gtcagggttggtccatgagcgtaggcgtgcgcggcatggactgaaccacgagtttgagtc

ggcttacgccacgacgtgccgagcgcacg-------------------------------

------------------------T---AGc-----G-TG-CActc-----------C-A

-----A--GA-AAG--Gg-T--

--G-CG----------A-CG

--A---TC---TTTGtgc-----GTcgtg-----------G-C-G-------TG--Gggg

gactccgtgtggagg---

-----------------------------------T--C--CTA----------------

---T---------ATATAtcaa--------------------------------------

-----------T-G-AA-T-------Aaagactcaa----------------A-------

--------------------GCTGTgc---------TTgtggg-----------CAgcg-

---------------A-C--A-AAt------C-----------Tct--T---GTc-TT--

-C----Tacgttttgatcttgtctgtgttct-----------------------------

-----G--TG-TT---Ctcgccgcgatcaccgcgatcaccaccagcgatccttgacatgc

ttggagcttcggg-------T---T-----------------------------------

--C--CAA

CA

>COPIA-86_SB_LTR

```
TGTT-GAAct-------------------------CAA----------------------
------------------------------------------------------------
------------------------------------------------------------
------------------------------------------------------------
------------------------------------------------------------
------------------------------------------------------------
------------------------------------------------------------
------------------------------------------------------------
----T-Cccg--T--GCa---T---G---------A-G--Tta-----------------
----T--C--T-GCA--TattgtaaTT--T-AG-TTtgt---TGCA---T---T-GGcac
gtttacaat---------------------------------------------------
------------------------------------------------------------
------------------------Tc--AGa-----T-AG-GAtag-----------C-T
G-C--A--TT-TGG--Gt-TGGgg-TG---------------------------------
------------------------------------------------------------
-------------------------------TTAG-------G-CG----------TcAG
A-Acc-AC---GCTGc-------GT---------------T-C-G-------TG--G---
------------------------------------------------------------
-------------------------------T---G--C-----gacgtagattctttcc
ccgccgtgtttgtaggaacaacccgtgattaggcgggcgtggcgcagcatgcgcagatac
ggcgggggggggggaaagatcatgtaaacaccaagcc-----------------------
---T---------ATAAAtagagaa-----------------------------------
-----------A-G-AA-A-------Aaacagaaa-----------------A-------
--------------------GTAGCgac--------TTtgtgg-----------CGgc--
---------------A-C--A-TAt------Ctt---------Tcg--T---GT--TC--
-C----Tgc---------------------------------------------------
------------------------------------------------------------
-----G--TG-TT---Ca------------------------------------------
```

--------------------T---Ctaacaaaccgaaacgcaaagtgagagagagttcgt

cggccaagccgatcgccgtcgtgcttgccattcaggcagccggcg---------C--CAA

CA

>COP20_LTR_MT

TGTT-AGAaat------------------------TAA----------------------

----AtCa----A--GA----A---A---------T-A--Tctagaagagt---------

----A--T--A-GAA--Gc------TTg-G-AG-TTa-----TTCT---T---T-TTcat

taagagttaaaaatatctatat--------------------------------------

------------------------Ta--TG------T-AG-CAccac----------C-T

A-G--A--AT-AAT--C--TTG---TA--------Accaaaagaataatctagtgctgat

tgtacaccacctctataggaataatctagcaccat-------------------------

-------------------------------CCA--------T-AGc---------T-AG

A-Aa--AA---TCTA--------GTaaccgaca-------T-T-A-------AG--Ttta

ag--

-------------------------------A---G--C--CTA----------------

---T---------AAAAGgcacatgcttgtaccatat-----------------------

-----------T-G-AA-Tcatca--A-------------------------G-------

--------------------TCTTCgacaataaaa-TTagtgtgtgtt------CAa---

---------------AgA--A-ATt------Ctcca-------T----T---GT--TC--

-Tc---Ttagttattactttgt--------------------------------------

-----G--AG-TT---Tgtgtcccactgatatcaaa------------------------

--------------------T---T-----------------------------------

---ggTAT

CA

>COPIA-36_SB_LTR

TGTC-TAA---------------------------TAA----------------------

----T-Ctt---T--GT------------------T-C--Ta------------------

----G--T--A-GAA--G-------TC--G-AG-TCg-----GGT--------T-AGggg

tggacatgaacttaggcgtgcaaggtcatgatcacccgagtcggtttaggactctcgcca

tggcgtgc--

------------------------Tc--AGcgcac-G-TG-GCg-------------C-A

-----T--GT-CGA--G--TTG---TA--------A------------------------

-------------------------------TC-G-------G-TG----------T-GG

Gc----AC---TTTGtat-----GTcatg-----------G-C-G-------TG--Gggg

gcttggactcaagcccgagcc---------------------------------------

-----------------------------------T--G--CTA----------------

---T---------AAAAGcaa---------------------------------------

-----------G-G-AA-T-------Aaacgatcatatg-------------G-------
--------------------GCTGCgg---------TTgcgg------------CAgta-
---------------A-C--A-ATag-----C-----------C----T---GT--TC--
-T----Tcatctcgtcgacttgctcttc--------------------------------

-----G--TG-TT---Caa---
--------------------T---Tctcgccgcggtgaccacgcactcgccggcgtccgt
tcctggcgcgttggaggcgttctgct----------------------------C--CAA
CA
>SHACOP18_LTR_MT
TGTT-AAT---------------------------TA-ttgcttttggactgcattacgt
ggctgatttatctttattccctat------------------------------------

------------T--GT------------------T-T--Cagt----------------
----T--T--T-TTA--T-------TT--T-AG-TCa--------A---T---A-AGtgg
tacctacactt---

------------------------T---AG------G-AG-AAag------------C-A
G-T--T--GT-TGT-----------TA--------Tcccacgatcctagt----------

-------------------------------TTA--------G-TG----------G-TG
A-Tg--TA---GTTGt-------GTtctg-----------T-T-T-------TG--T---

-------------------------------T---T--T-----gtgaaacac-------

---T---------ATTAAgtgtattgttttag----------------------------
-----------T-G-AA-Att-----A-------------------------Ataaat--
------------------------Caa---------TTtcc-------------CAtt--
---------------C-C--A-TTt------Caacg-------Tat--T---CT--TC--
-Cc---Tgtctatcatatta--

-----T--TT-TT---Ca--
--------------------T---Ttataccatataa-----------------------
--C--CAA
CA
>COPIA-20_SB_LTR
TGTT-AGTa--------------------------TAAgcctgatgtgttcccaactgga
ggacacactgtggtgaagctggagaagcgaaggctgcagtggatttcaaatgggagcgtt
ggcgggaacggttgctggacggggtggagcggggtgaccgcgaccacgacggttgtctcc
gtctgatagtgtgtttttagccaacttgta------------------------------

----T-C-----T--GT--------G---------T-T--Ta------------------
----T--T--T-GAA--T-------TT--G-AA-TTa-----TGT--------A-AGcgc
gtgtgttagtgactggtataag--------------------------------------

------------------------T---TG-------cTG-GAtg------------T-A
A-G--A--GG-TGT--G--TG---------------------------------------

-------------------------------TTAGc------G-CT----------T-TG

AgA---CA---CTTC--------GT---------------G-T-G-------TG--C---

-------------------------------T---T--T--GTA----------------

---Tcgtgtccac---

-----------T-G-AA-Aggcg---A-------------------------Aaaggggc

gcccgtcaccctggcggtt-GCTGTgtg--------TTcattcagtgtt-----CAt---

---------------C-C--AgATct-----Cg----------Tt---T---CTt-TC--

-T----Tgcgcgatcttgagtttaggcatagccactgt----------------------

-----G--TG-TT---Ccagctcgtga---------------------------------

--------------------T---Tgtcgatcctaaa-----------------------

--C--CAA

CA

>POPCOP1_LTR

TGTT-AGTtat------------------------TAA----------------------

----A-C-----T--TA------------------T-T--Tatcaaga------------

----T--T--A-GTA--T-------TAaaG-AG-TCata---ACTA---T---T-AGtat

tgaagagttgcatctattagtattaaagagtcataactattagtattgaagagttgcatc

tat---

------------------------T---AG------T-AT-TA----------------A

A-G--A--GT-CAT--Aa-CTA--------------------------------------

-------------------------------TTAGtattgaaG-AG----------T-TG

C-A---T-----CTA--------TTa--------------G-T-A-------TT--Gaag

agtcacaactattaa---

-------------------------------T---G--T--CTA----------------

---T---------ATAT---

-----------T-G-TA-Tg------Aatctcataatgaaa-----------A-------

--------------------AGTGTg----------AGag--------------TA----

---------------C-C--A-TTtt-----Ca----------Ta---T---GTa-TTg-

-Ta---Ttctaccaaattagagagtgtgcaacctaaatc---------------------

-----C--TT-TT---Caattctattctctatttctgccaaat-----------------

--------------------T---T-----------------------------------

--C--CAA

CA

>COPIA-32_SB_LTR

TGTA-GGCctc------------------------TAA----------------------

----T-Tgt---T--GC----A---Gg--------A-G--Tctagtaagtttc-------

----G--T--A-TTA--G-------TA--G-AG-TCcag---GGTA---T---T-TGgtg

a---

------------------------T---AGatcg--T-AG-----------------C-T
G-A--A--GT-CAT--Gc-TTG--------------------------------------

--G-AT----------A-CG
G-Tg--TA---TTGGa-------GTcgga-----------G-T-C-------TG--Taaa
caccacgtcg--
-------------------------------T---G--C--C--gtccatggcgcatgag
acgcgcgtcgcagggaaacggcgtagcgtaccaggtctgtacgacgtgggaggggagtct
catgag--
---T---------ATAAAtact--------------------------------------
-----------T-G-AA-T-------Aaagatgtcatgag------------G-------
--------------------GCTGCgg---------TTggg-------------CAgca-
---------------A-C--A-TAat-----Ctctcttgcc--Tg---T---GT--TC--
-T----T---

-----G--TT-TT---Cttctag-------------------------------------
--------------------T---Tttcaatctactcgctggtagtcacgatcaccgcga
cgccgacgcgttctgcgttcgtcgttccaatctgatcagtcgtgatcaggct--C--CAA
CA
>COPIA-87_SB_LTR
TGTT-GCA---------------------------CAA----------------------

----G-Tt----Ta-GT------------------T-T--Cctag---------------
----T--T--T-GAT--T-------TA--T-AA-TA------TGCA---T---T-AGcta
ttgta---

------------------------T---AG--------GG-CAga------------T-C
G----A--GT-CTTa-Gg-TG----TA--------Caagaactcggtcaggctctagagt
ccggtctgtccgcgtcacgccgcgcacggcgtacatggcgttcccgctgagcaacggtgt
aacgcacctgttcgggtgacgcggagtcggaTC---------G-TG----------T-TG
-------A---TCCAg-------GT---------------C-T-A-------TA--Ttt-

-------------------------------A---G--GcaCTA----------------

---T---------AAATAca--
-----------T-G-AA-T-------Aaaggaccaggca-------------A-------
--------------------GCTGCacg--------TTttgg------------CAgcg-
---------------A-C--A-AT-------C-----------Tc---T---GTc-TT--
-C----Tcgcgagtttttacgttctagc--------------------------------

-----C--AG-TT---Cacg--
--------------------T---Tctcgccgcgatcaccgcgtcgacgacttcgccgtc
gtcgtgcccaccgctcgctgttctagct--------------------------C--CAA
CA
>COPIA-49_SB_LTR
TGTTaAGT---------------------------TAA----------------------

----G-Tc----T----------------------T-T--Ttcattcctaggtgcatgag
tcaaG--T--T-GTAa-Tccag---TT----AT-TAgc----TGCA---T---T-AGtta
agatttagcgcg--

------------------------T---AGaacct-G-AG-GAttgg----------T-T
A-G--A--GA-TAT--Gc-CTA----Accatc---T------------------------

-------------------------------TTAG-------G-C-------------CG
C-G---TA---TCTAgg------GT---------------G-T-G-------TG--T---

-------------------------------G---G--T--C--accgccatcgcacgtt
cgtggtgcatgtcgtacaggccaagaaaggatctgtacaacgattgtgtggtggcggggg
acgggctccaagcccatgtatccatgtg--------------------------------
---T---------ATATAaagcatga----------------------------------
-----------A-G-AA-A-------Accctgaaa-----------------A-------
--------------------GCTGCggctg------TTgag-------------CAgcg-
---------------A-A--A-ATa------T-----------Cg---T---GTc-TCg-
-T----Tccaatctaatctgtgtgcgtgcgtggtattcctggtcatcgtgtaggcacgcc
gccggc--
-----G--CG-TT---Ccaggagctagcaggccg--------------------------
--------------------T---Ccagct------------------------------
--C--CAA
CA
>COPIA-97_SB_LTR
TGTA-GCCg--------------------------TAA----------------------

```
------------------------------------------------------------
------------------------------------------------------------
------------------------------------------------------------
------------------------------------------------------------
------------------------------------------------------------
------------------------------------------------------------
------------------------------------------------------------
----G-Ccc---T--GC------------------T-T--Cccaggcacat---------
----T--TagT-GTA--T-------TT--A-AG-TT------TTTAg--T---T-AAtct
atgtcattaagtcc----------------------------------------------
------------------------------------------------------------
------------------------T---TGgg----T-AG-CC--------------C-A
A-G--A--GT-TGT--G--CGGg--TA--------C------------------------
------------------------------------------------------------
-------------------------------CCAGgca----G-CTat--------T-CT
G-Tc--TA---GTTAaa------GC---------------T-C-T-------TG--Tgag
ctgtatgtaatcaatgctatgtctgcct--------------------------------
-------------------------------T---Tc-C--CTA----------------
------------------------------------------------------------
------------------------------------------------------------
---T---------ATATAt-----------------------------------------
-----------T-G-AA-Gg------Agatgcctggggtc------------A-------
--------------------GTTGTc----------T-----------------CAagg-
---------------C-T--A-AT-------Ca----------T---------T--TT--
-C----T-----------------------------------------------------
------------------------------------------------------------
-----C--TG-TT-----------------------------------------------
---------------------accT-----------------------------------
------------------------------------------------------C--CAA
```

CA

>GMCOPIA11_LTR

TGTT-GAAata------------------------TAA----------------------

----A-Ct----T--GA------------------T-T--Tgggcctaaat---------
----T--A--A-TTA--T-------TT--G-GT-TCct----TGGAc--T---T-AGtta
ttttgggcttaagtaattatgggtcatgtttc----------------------------

------------------------T---AG--------AG-AAtt------------C-T
-----T--GT-AGT--Gt-TTG----G--------Agtgtctagatatttcttatggttk
taatattctctagaatactctttggatct-------------------------------
-------------------------------CTAGa------G-TT----------G-AG
A-Ac--TC---TCTAgaa-----TTa--------------G-T-G-------TG--Tcta
gagttctccttag---
-------------------------------A---G--Ta-GTA----------------

---T---------AAATAgaga--------------------------------------
-----------T-G-TA-Atcct---Acac----------------------A-------
--------------------TTTGTa----------TCaag-------------CA----
---------------A-A--A-ATa------Caaa-----------------GT--TC--
-Tc---Tcctccataaagaattctccttcctatcaagtttctattcaaagtctccaatat
tcctaaaca---

-----C--TT-TT---Cctaaacataaaaagccttat-----------------------

--------------------T---T-----------------------------------

--C--CAA

CA

>COPIA-11_SB_LTR

TGTA-GAAgt-------------------------TAA----------------------

----GtCc----T--GCtcccA---G---------T-T--Atcacttagttatctgt---

----G--C--T-TAA--T-------TA--T-GT-TTc-----TGC--------T-AT---

------------------------Ta--AGgcc---T-TG-GGcagca---------C-A

A-G--T--GAcTGT--Gc-AAG---TA--------C------------------------

-------------------------------CCAG-------G-C-------------AG

--T---TA---GCTGt-------TTa--------------G-T-Taa-----TG--Ctct

aatg--

-------------------------------A---G--C--C--atcaatgtaatcaatg

tgctacttatctcc--

---T---------ATATA--

-----------T-G-AA-Ag------Agcagtcgcat---------------G-------

--------------------GGTGTg----------TTatcc------------CAggct

ga-------------A-C--A-AAaca----C-----------Tc---T---GTa-CC--

-T--

--C--CAA

CA

>COPIA-71_SB_LTR

TGTT-AGC---------------------------TAA----------------------

----A-Ttat--T--GT----A---G---------T-T--Agatatatagaaac------

----T--T--T-GTT--C-------AA--T-TG-TCa-----TCTA---G---A-GTccc

ggtagagg--

------------------------T---TGtgcgt-G-AG-CA-----------------

--G--A--GG-CGT--Gc-TTG--------------ttcacgacacgatcgagttgtgtc

ggtttgtgccgcgccatgacacgagctgcagcgcgtggtgcgcacgccgcaggaacggtg

tcgtacacggctctgatcgtg----------GC---------G-CGg---------T-GG

G-A---TA----CTC--------GGag-------------G-C-A-------TG--Ttcc

gagatctcgtcatgtacc--

--------------------------------------C--CTA----------------

---T---------ATATA--
-----------T-A-AA-T-------A-------------------------Aagtggac
cgaacaac------------GCTGCg----------ATatgagg----------CAgca-
---------------A-C--A-TAtt-----Cattt-------Ta---Tc--GT--TT--
-T----Tgtctctgtgaactcgtcgtagactcgccgcggtcaccagc-------------

-----G--TG-AT---Ca--
--------------------T---Cgccggcgtccagtattggtgcagggcagagcctac
g---C--CAA
CA
>COPIA5-PTR_LTR
TG--

------------T--GT--------------------T--T-------------------
----G--T--T-GCA--T-------TA--A-AC-TCaa----TCTA---G---A-AGgtc
aaggaagacaaccaccac--

------------------------T---TG------T-AG-CCac------------C-A
G-C--T--GT-CATc-GccTAG--------------ccaccacttgcaatcgccagccgc
cagccaccagctgtatgccgccagccgccttcacac------------------------
-------------------------------TT---------GaAGg---------T-TG
A-At--TA---TCTT--------GT-----------------T-T-------TGaa----

-------------------------------T---TcaT--GTA----------------

---T---------AAATAggtacctaagtggatgctattctgtgtggaaagagaggaaag
aaacactagaaA-G-AA-Agagag--A-------------------------G-------
--------------------GGAGTgta--------TT----------------------
-----------------C--A-AAg------C-----------Ttt--T---GT--TTaa
-Tc---Tttgtaagctttttattgttgaaataaaacagt---------------------

-----G--TG-TT---Ttataccctctgaatgtttcaaagccaccaccagtggtttc---
--------------------T---C-----------------------------------
--C--CAC
CA
>COPIA-94_SB_LTR
TGTT-AGC---------------------------TAA----------------------

----A-Tc----T--GT-----------------T-T--Tagataagtttgg-------
----T--T--T-GGA--Tctcg---TA--G-AG-TCc-----AGT-c--G---A-GT---

------------------------gT---AGgagatcG-AG-CAttc-----------C-T
G-A--A--GG-CAT--Gc-TTG--------------gggacgactcaa------------

-------------------------------TC---------G-AG----------T-TG

G-Ag--TC---TGTCcgc-----GTcgggatacaactgctG-C-Acg-----TG--G---

-------------------------------T---G--T--C--cgtcgctcgaacgacg

tcgcacacggatgtaacttggcgcgggggataagcggagcctgttccgtgatctccat--

---T---------ATAAAtaa---------------------------------------

-----------G-G-AA-T-------A-------------------------Aacatgtt

ctaaaac-------------GCTGCaacg-------TTgggcagcg--------CAaa--

---------------A-C--A-TAt------C-----------T----T---GT--TTg-

-T----Ttcattgtgatttccaatctgctaacgtcgaactcgtggtcaccacgccggcta

gtcgccgtcgcggacatagacatatttata------------------------------

-----T--CG-AT---Ccttgg--------------------------------------

--------------------T---T-----------------------------------

--C--TAT

CA

>COPIA-109_SB_LTR

TGTTaAGTtcgtttagatttcctttcatg------TAA----------------------

----T-Cc----T--GT----T---A---------T-G--Aacaa---------------

----T--C--T-TTA--G-------TT--T-GT-TT------GGT--------A-AGtcg

catagaatct--

```
------------------------T---AG---------G-CAat------------C-T
A-G--A--GA-TAT--Gc-ATG----G--------A------------------------
------------------------------------------------------------
-------------------------------TT---------G-CGc---------T-AG
G-A---CC---GC-Gcac-----TG---------------G-T-T-------TG--Ttac
cccgtcaaggcgtcgtctgcatgtcccacctgcgttccggaaataacgggcgatgggtgg
gggcgccgtagcgacggggtggaaggcctcgA---G--C--CTA----------------
------------------------------------------------------------
------------------------------------------------------------
---T---------ATATAc-----------------------------------------
-----------T-G-AAcA-------A-------------------------Aagctaaa
ggaatggtttac--------GCCGTg----------TTggg-------------CA----
---------------A-Ag-A-TCgt-----Ctt---------Tg---T---GT--TC--
-T----Ttcagctcacgctctagggttctagtcgccacgatcaccacgtgcaggtgccgc
gccgtcgccggacttgctagcgc-------------------------------------
-----G--AG-TT---Cgg-----------------------------------------
--------------------T---T-----------------------------------
------------------------------------------------------C--CAA
CA
>CPSC2_LTR
TGAT-AGCtttatccaaa-----------------TAA----------------------
------------------------------------------------------------
------------------------------------------------------------
------------------------------------------------------------
------------------------------------------------------------
------------------------------------------------------------
------------------------------------------------------------
------------------------------------------------------------
----T-Ct----T--GT--------------------G--Tgcgaaagctagtt------
```

----T--T--T-TCA--G-------TT--GcAT-TTt-----TTTAac-T---T-AGcca
ttgcattttgtaa---

------------------------T---AGca----T-TG-GAttc-----------C-T
G-G--T--TG-CATgcGc-TGGc--TG--------Aagattagcaaactcgg--------

-------------------------------TTA--------G-TGt---------T-AG
T-Ta--TC---GTTT--------TT---------------C-C-G-------TT--T---

-------------------------------T---T--T--C--ggtagtagaagagatc
gagcagggatcggatcgtgggatggcacggtcagtcgttgatccggcaagatcgcgcatg
taacagactcttttgtggaggtgaa-----------------------------------
---T---------AAAAGga--
-----------A-G-AA-A-------Aaccccagaaa---------------A-------
--------------------GCTGCgaag-------TTttt-------------CAgca-
---------------C-C--A-AAa------C-----------Tc---T---GT--TT--
-T----Tgctctgtttttctatgttgtcgtattttcgtcagatactgttcatcgttctga
actactcagacaagctaggatagccacaatctcgcgt-----------------------
-----G--TG-TT---Cagagtg-------------------------------------
--------------------T---Tctag-------------------------------
--C--TAA
CA
>hmmcons
TGTT-AGA---------------------------TAA----------------------

----T-C-----T--GT----T---G---------T-T--T-------------------

----T--T--T-GTA--T-------TT--T-AG-TC------TGTA---T---T-AG---

------------------------T---AG------T-TG-CA--------------C-T

G-G--A--GT-CAT--G--TTG---TA--------A------------------------

-------------------------------TTAG-------G-TG----------T-AG

T-A---TC---GCTG--------GT---------------G-T-G-------TG--T---

-------------------------------T---T--T--CTA----------------

---T---------ATATA--

-----------T-G-AA-A-------A-------------------------A-------

--------------------GCTGC-----------TT----------------CA----

---------------A-C--A-AA-------C-----------T----T---GT--TC--

-T----T---

-----G--TG-TT---C---

--------------------T---T-----------------------------------

--C--CAA

CA

…………………………

Sushi_150

>GYPSY70_LTR_DR

```
TGTCAG-A--a-C-----Ccgg----G----------TTTgaa-----------------
--------C---T----T--TGccc--TcTTTc-T-TTT-Ctgacagcgttttctatctt
tccttactgacaacattctaatttgtctccaaattgctgtgtaactttactcagctgatc
gtcttttacaattagtagcctcaca-----------------------------------
------------------------------------------------------------
------------------------------------------------------------
------------------------------------------------------------
-----------------TG-------TTa-CTg-C-CAGcTGCCTT------TCATttct
gctaattgctgctc----CT-A---TAT--------CT---GAgggtgTTTCGCt-T-C-
---TcCtT-TTG-TC-A-GAgTgttggatttcttacagccttcttgtcggtctgttgtat
tatcattgtgttctctgtccagttccctggatccatcccggatttggtctgccctactgg
agagctgatcttttgcgttgcctttgcccttctcctgcattaccaggcctggaccaaaaa
ctaactttattttttgattgaaatttagcccactcctgatttt-----------------
------------------------------------------------------------
------------------------------------------------------------
------------------------------------------------------------
--------------------------------------------------TCTT--TTtc
atgtggtgctactaat-----------------T--T-TG-T------T--Gg-TA--T-
--T-T--TT-----------CCTgtactgttgaacattgcctgtggtccagttttcagtt
tattttttactaaagttcaagatctgctctgggatctcagcaagcgctgagtcactttc-
------------------------------------------------------------
------------------------------------------------------------
------------------------------------------------------------
------------------------------------------------------------
------------------------------------------------------------
------------------------------------T-T---T--T-T-Gct--------
---------------------------T---ACTT-T-------AT---TAaaaaaccct
a------------------------------------T---Tga-ATTGA-Ac--TC--C
Tctg----------------------------------CTTTT--Gg---G---T-----
```

-Cctttatacagacaca---
---C----
CTGACA
>GYPSY72_LTR_DR
TGTCAG-GaCa-C-----Tggctt--G----------ATC--------------------
--------T---TtctcT--TCtacc-TcTTCc-T-GCT-Tttcattcttgcagcgtgca
ctcagctgatcattattacgaatcagtgcgcacacggcttgctgcacctgttactggtct
ctgttaatttccttgctatttaagctctcctttcccactttctggtcgctagtgtgtcc
tgtcattattcaggctatctcgctgcgttatct---------------------------

-----------------TG---A---TTc-CTg-T-CA-tTGTCAT------GCATtact
tcatgttctgatt-----CT-G---TTT--Tt----CT---CTt----CTGCCC--A-G-
---T-T-T-TTGcTC-G-GT-T--------------------------------------

--GCCT--GTgt
---------------------------------T--T-TT-Tc-----T--G--TT--T-
--C-A--TT-Ttggat----TCTccccggccagccacctaccttcgacttcgcctgtccg
acgctgaacttgactttcctccgttatcccatctgcctcccgatcgatgcttgcctgcct
gacctctctcacgcccgctgatttggacactgttgccgagttgcctttcgctcccaccga
gcga--

------------------------------------T-T---TcaT-T-T----------
---------------------------Tc--ATTT-G-------AG---TCacgagcctt
ggctcgtcatttgttttgagaag--------------T---T---AATAA-Aa--TC--C
Ttttgtgttaacagcatttgcgtttggatc--------CGTCT-------C---T-----
-C--
---Cgggt
GTGACA
>GYPSY76_LTR_DR
TGTCAT-A-Tt-C-----T-------G----------AGC--------------------
--------T---A----T---------TaTTT----GTT-Ttctccacaagatgtcgcca
ttttccccagtgtttctgtttgtggttcatttgtgt------------------------

-----------------TA---A---TTt-GGt-T-CAGcTGTGTC------TTGTtaag
actagc------------CT-A---TATA-Tt----CTa--CC-----CTCTTG--T-Ct
---T-C-AcTTG-TC-A-CT-Tggttttgagtttgatgtgctaccgctgttgccgtaag

--TCCTc-TTgt
tat------------------------------TacC-TT-T------T--G--TA--T-
--T-T--TT-Ttg-------GCTatagtaccagttgctctacggagaactttatgtttgt
taactgcttagcttttggagttcctatgtttttgcctggcggct----------------

------------------------------------T-T---T--TaT-T----------
---------------------------GatgATTT-Tgga----TT---TC---------
-------------------------------------C---Tt--AATAA-Ac--AC--C
Tttgtttgaactagac----------------------TTTGTg-C----C---T-----
-Tagcaattgggttcaccaactctccctgtggctcatcgttaaaccactggtctactaca
ccagcgacccgggttcgagccttgggct---------------------------C----
ATCACA
>GYPSY107_LTR_DR
TGTCAG-G--a-C-----TcctggctG----------ATCggctgg--------------
--------T---T----CtaTC-----TgTTC--T-GTT-Tgtattagccacatgttcct
ttgtttgtgcttctgattgtttctctccacctcctcgttctcataattttagt-------

-----------------TG---A---TTtgCT--T-CACcTGTCTT------TGCTtatt
ttctcctcattagctcccCT-A---TTTA-Tt----CT---CTggg--TTTCTGc-T-Gt
---T-C-A-GTG-CC-A-GA-Ttgttgtttgcttctgaccctgcaacatcaactataggt
aaacttaatgtttttagttaacctttttctgtttgactccaggactctctccctaccttc
ccagcctttctgtgtagtccctggcttgctcgaagtttttgctcttgtgctgcctgagtt
tttgatgtcctggacactggcgtggttctgacaattacacctgcctctgtttctactggc
cagtctatttttttgttcctggttatttccagtcattctcttcccctccatcaaagtctgc
aagtatttttttttgtcatttttttgttgttcatttttttttggactataatattttttgtt
ctttcttgatcaggctgagaggactgcttgctgaagagcattt-----------------
--TTTT--TTcc

ccatttgtcgaactgagg---------------T--T-TT-Tg-----T--G--CA--T-

--T-T--TT-C---------CCTtgagactgagacattgcctttagaagattt-------

------------------------------------T-T---T--T-T-T----------

---------------------------Tca-ATTT-Tgca----TTc--TGg--------

-------------------------------------C---T---AATGA-ActgAT--C

Tgttagaa------------------------------TTTTTg-C----C---T-----

-Tccacaataaatcattgtcttcacctgcatttgggtttttctgtttactggc-------

---C----

TTGACA

>GYPSY105_LTR_DR

TGTCAC-AaT--C-----Tcggtct-G----------ATCac------------------

--------Cgg-T----T--TGa----TgTTTg-T-GTT-Actttttccctttcactcgt

attgttttgatcactcataccctgctttgtttacctctcgcttccccacccatctgctct

attactttccattagtctatcaaac-----------------------------------

-----------------TA---A---TTt-CAc-T-CACcTGG-TT------TCCTtgat

ttctcccc----------CT-A---TTTA-Tcc---CT---CTg----TTGATCg-TaGt

---T-C-T-GTG-TC-A-GA-Tcgtagtttttccccttccggcatgtctggcgatctcct

ggtctagtcctgttccttgtatatcctgtgagttttgtcgtgtctgatgtca--------

--TCCTg-TTga

---------------------------------T--T-TT-CctaaagT--G--TT--T-

--T-T--TC-Tccgtctg--CCTtcaagcctgttcccgtctgtttttgctttgccgccca

tctgactctccgtccctatcctgccggccctcaacactctctcgccggcttccaagcctc

agtcttcctcgtggccattctgtcccagtcagcggcggccggattcatcgccccctgtcg

ggtatcagggtattgcaccctcttgaagactgttcatctaacctctccggagtagtttta

tttaatcgtttttggagactttatttctcagttttgagtttttgtgaactttgggctttt

ttgccaactccattacggaggaatttttagtttccttttttgaagattg-----------

------------------------------------T-T---T--TgT-Tgac-------

---------------------------T---ATTT-T-------TTt--TGccccttgag

tttgtgggc----------------------------T---Tc--AATAA-A---CT--T

Tgctgagacctgcatctgtgtctgaaccatcttc----TCTCT--Ca---C---Tcagac

-C--

---T----

CTGACA

>GYPSY62_LTR_DR

TGTCAC-A-Ga-C-----Tc------G----------GTC--------------------

--------C---Ca----------------------GTT-Aatcccctcaacaaccagca

gaggtcccgctcacccgactactgaagttttaagaactccattacccacaagaccccgcg

ggacct--

-----------------TG---A---TTa-CTg-TcCAGcTGAAAC------CCATacac

acaaacg-----------CT-----TATA-Agcc--CTt--CT-----CGCCTG--T-Ca

---TtC-T-GTG-CGaA-GTcTtgttttgctctgcattcaattccaagcgttttccctac
attatctgactgatttctgttgccgacccggactgtttgtttacctgtgattctatgccg
cctgcccagatcctt---

--GCCT--GTat
cc-------------------------------T--C-GTaTa-----T--GtaTC--T-
--T-T--GC-Ta--------CCTgcctacaactctgcttgtgacccgactttgagtgagt
tccctgatctgatcttctcgtttgtgaaacgtacat------------------------

------------------------------------T-Tgc-T--TcT-T----------
---------------------------T---ATTT-G-------TGttgTG---------
-------------------------------------T---Tc--AATAA-Ag--ACg-C
Tgcaaatggatcccactt--------------------CGTCTgaCt---C---T-----
-T--
---C----
ATCACA
>GYPSY1_LTR_GA
TGTTAT-G------------------------------TC--------------------
--------CagcC----C--TGag---TaTTA--A-GTT-Aagttggcgggctatttata
attcttcggttatgtgttattgtttactcacctctcgtttcagattcctcactccctccc
tcaggtgtgtgggttccacctgggtgattgtcagcccctccc------------------

-----------------TG---A---TTg-TT--TgCACcTGGCCC------TCATcacc

cggt--------------GT-A---TTTA-Gt----CT---GTggg--TTCCTG--T-Cc

---T-CtG-TTG-CC-A-GT-Tcgtctttgatacaatgttcctagcgttccagcatttac

tcctgatagcctccctgttaccgacccct-------------------------------

--GCCTggTTaa

cgcgtcacg------------------------T--C-T--C------T--G--CC--T-

--T-T--TC-----------CCTgccggataccttgcctggtgatcgactgcctgcccgt

gtaccgaccttggaccggaacgtttacgacctttgcacctacctgcctcctgtcgaattc

ttacacccgtgtttgtactgccatccaaacggtaaaattgg-------------------

------------------------------------T-T---T--AcT-Gcaac------

---------------------------T---ACTT-G-------TG---TCtccgagtcg

tg-----------------------------------C---A---ATTGA-A---CC--C

Acacc---------------------------------CTTGT-------C---Tgcac-

-C--

---C----

CTTACA

>GYPSY4_LTR_AO

TGTTAC-A-Ga-Ccc---Tt------G-----------TC--------------------

--------T---Ag---C--GCa----Cc--------TT-Atcgaaggcggacacacaag
gcagaataacgataggatgacgatggccgattcctgagtggaaggcacccgacgaatcgt
ttatctagtagttattcggaaagggctacggacgccgataccagctaccacaacaaagtc
acgtgcatcgtagcagtgacacgaccaaacgtatcagcgacacgacaagctcgtagcact
gacaaga---

-----------------TAc--A---TCg-TA--T-CAC-TGACACgaagagTCGTagca
gtgacacgacgggcaaggAT-A---TATA-AggacaCT---CA-----TTTATGt-A-Ct
---T-A-A-GT--TT-A-GT-Tcttcgtgatcaatacacatggttactacagagttacct
ttagtagcaagaccagtctcaactcgttcgcactcttctaactattaagtatacgtactt
aataggcctgg---

--CCCT--TTgg
actcgagtctagtatcgacggcag---------T--C-TT-G------T--G--TCga--
---cT--GCaTc--------CCTgtgacgacggcagtctagtatcgacggcagtctagtg
tcgacggaagcctcataacgactcccgtcttccccagaccgcaacccaacagcgacggag
gtcaaagaacgac---

------------------------------------T-Tcag------------------
--G---------
-------------------------------------T---T---AGTAA-----CC--C
Tcgaaa--------------------------------CGTGG-----------T-----
-Ccgctaactggtctcagacttgacgacgaagccccggg---------------------

---C----

GTAACA

>CGRET_LTR

TGTTAC-G--a---

--------C---C----C------------------GTT-Atgcaggataacagttaacc

gacccacttgggcatcgggcggctagaacaggacatttgtactgcaggatcacgggaaca

acagggactcactcggaaacggaagaactatggtacaagtcacgtgatgataaggaatga

gcatagataagggatccgcaatgagcgctcatcgggtatcggaatgagcgctcatcccac

ttcagaatgagcgctcatagtgaccaagggtacagcgctcatgacaataagggccattca

gttaggaagacttgtatatagtatgacctgagcccggctcaagatcagactcccatagct

cttcagcaatcaacttgTG---A---TTa-TCc-T-CAG-AGATAC--------------

-------------------------TTTC-Ag----CT---CAc----CTTCCC--T-Gc

gaaTtA-C-TTG-TTgA-GTcTaagactgatcacctta----------------------

--GCCT--CTga

agac-------------------------------------T------T--G--AT--C-

--T-A---C-Tgcag-----CCTacgaccaacgaccacgaccccagtgtac---------

------------------------------------T-G---T--T-T-Aaacgtcaatc

ccc------------------------A---ACTTc-------------TG---------
-------------------------------------T---T---AGCAG-A---CC--C
Tac-----------------------------------TGTCG--GatagC---Tga---
-C--
---C----
GTAACA
>GYPSY5_LTR_DR
TGTTGT-A--a-A-----Tgagggc-G----------ATGccaa----------------
--------C---C----C--TC-----T------T-GTG-Caccaccagagggaaccatc
gccagaattctgattcgactcacggactcaaaatcccataagccctgctacctggcac--

-----------------TG---A---TTa-CGg-TcCAGgTGCAAC------TCATcagc
tctcgt------------GT-A---TATA-Tac---CG---CA-----CTCACGc-T-Cc
gg-TtC-G-TTG-CGaA-GTcTtgatttgcctggctgtcatttctgagcgttccatactc
cctgcttcggactgatctgtgtttctgaccctgtgcttgttctacgattacgaaagacat
ctgcctgcccctgatctccagcctgttattctgaccagtaagatatccgcct--------

--GCCT--TTga
act------------------------------T--T-TG-Cc-----T--G--TC--Cc
acG-T--TC-Tgtct-----CCTggattgcccctttgtgt--------------------

------------------------------------T-Tg--T--T-T-G----------
---------------------------T---ATGT-G-------TGc--TC---------
-------------------------------------T---T---AATAA-Ag--CTtgC
Aaatggattcaatgc-----------------------CTTCT--Ga---C---Tca---
-T--
---C----
ACAACA
>GYPSY89_LTR_DR
TGTAAT-A-T--C-----Tca-----G----------CAC--------------------
--------T---T----Ta-TCg----Ca-----------Tcctccaacccaccaggggg
tgcacatgttgccctatactcctcaacattagatggcagtaatgactcttgttcctctaa
actctcctgtaatcagcg--

-----------------TA---Acca--c-CA--C-CACcTGTTCT------TTGCt---
------------------CT-A---TATA-Aga---CTg--CTt----TGTATGc-T-Gc
---T-C-T-ATGtTC-A-GT-Cctgaactaaacctcttgctgccagttaagttgttctca
agactttttgcttgttctcaagacttgtttgctattttgctgtttgattgttttgagacc
ctcgtgtctattttattttgtatgtattttcaggaagctcggctt---------------

--TCCTa-TT--
---------------------------------T--T-TG-T------T--G--TA--C-

--T-T--TT-Caa-------CCTtgctgtagagagatattatctctcc------------

------------------------------------T-T---T--T-T-G----------
---------------------------TcaaATTTa--------------C---------
-------------------------------------C---Tc--AGTAA-Ag--AC--T
AtattattttttttggaaattcatcagttgtggactcacTTTGT--Tc---C---T-----
-Cctctgctcttgggccaccagctgctgacttagctcatctagtaagatctactgctaac
cacaccgcagacccgggttcgatccccactcagat--------------------C----
ATCACA
>GYPSY96_LTR_DR
TGTCAC-A-Tc-C-----Tcagtct-G----------ATCatgctgt-------------
--------C---Tg---T--TCcc---CaTTTc-T-GTT-Tacttctggtgagcgcacgt
gaatgtttattttgatcgctccccgtatctctcctccctctcgttaccctatttaccatc
tattaagat---

-----------------TG---A---TTttCAc-T-CACcTGCACC------CTATcagt
gattcctctcgtgtc---CT-A---TTTA-Ttc---CC---CTc----CTTGTG--T-Gt
ag-TcC-A-GTG-CT-A-GA-Ttgttgttttgcgagcgcgcccacgccgagccgctcgcg
tctgatctcgtcttgtctcctgtcgtggctctcctgtcctttccaactctgtcttgttgt
gctgctccacgccagaaccatcttcttgcactttgcctctccttaacctcctctgttccc
aggccggactgcttcaacctctcggagcccatcggcttcaactgactgccggcttcctct
ccctcgctcacgctccacttccacattcccctcacggcaacgtaccagcacgccccctgc

cgggtaacagtatcatagacgctattgtttgcatttatttttggatcaccccggctgtct

tttgctttctctttttttgaagattgtgaactgggcgcccccttttggatttatttggtga

caggacccggtctctgttgtcaaggacttaacagcgccctcttctgggagTCTT--TTtt

tatgcagt-------------------------T--T-TT-T------T--G--TT--T-

--T-T--GT-Ttttct----CCTattttttctgcaaacgcccagttggatttgatttgca

ccc---

-------------------------------------T-T---T--T-A-G---------

---------------------------GgtaATTT-T-------TTga-TC---------

-------------------------------------A---Ag--AATAA-Aaa-CT--C

Ttaacctgcacttgtgtccgcc----------------TGTGT-----------T-----

-C--

---C----

CTGACA

>GYPSY103_LTR_DR

TGTCAC-AcCc-C-----Tg------G----------TTTgatcacgccgc---------

--------C---T----T--TG-------TTTa-T-GTTgTcatgtcattagcacgcgtg

gatgttttgttttggtctgtcaccttgtgctcaacgtaatattcctccctcccccttgtt

accttatttactattatcatgatt------------------------------------

-----------------TA---A---TTa-ATc-T-CACcTGGATT------CAGTtatg

ctccttccc---------CT-A---TTTAgTtcc--CT---CT-----CGTGTG--T-Ca

---TcC-T-GTG-TC-A-GA-Tcgttgtgtgtcagtccgtgtaaacagtcttccgagttg

tctctccagtcctgtcgtgttggttggtgttatcccttccagttgtgtctcgcctggccg

tcatcttacttctagcgctctgtccttcttgtgtgtgttccattttggctcatatccctc

tgttctcaggcaccgtcatcccctccgcactgagttctccgacatcacctgccgtccgac

ttcccctctccctcgctcgctctcgtcggtcacatctaaaacagccgggccatagtgcgc

cctctaccggaggagtttgtaaggacattcttttctcattatatttttgctgtcttaacc

ccg---

--GCCT--TT--

------------------------------------T-TG-T------T--G--TT--T-

--T-T--TC-T---------CCTtgtgtgcagcgcgccactggccgagagactctgtttt

ttgaccctcggaaatttttatttttctcttttcaaacccggtaacggaggaggtcctaaa

gactatttatttgatc--

------------------------------------T-T---T--T-T-G----------

---------------------------G---ATTT-T-------TG---TGttatttttt

ggctgttattttgtggccctgactacagtctttgctgT---Tc--ATTAA-A---AT--C

Tttaatttgaacctgcatt-------------------TGTGT--C----C---T-----

-Tccagtcccatt---

---C----

CTGACA

>GYPSY88_LTR_DR

TGTCAG-G--g-T-----Ttt-----G-----------CC--------------------

--------C---T----T--TGt----CaTTTt-A-GCT-Tttcctgtaatgcttttttt

tgtccactagatgtcgtcatttagtagttttctatttggtatccgtaatcattg------

-----------------TC---A---TTg-ATt-T-CACaTGTGCC------TTGTttag
tttctt------------GT-A---TTTA-AgtagcCT---CAgt---TTCATC--T-G-
---TaC-T-TTGcTC-A-GT-Tattgtggattcaagcctgtcaaccactgtgagttactt
tttgtaaa--

--GCCTc-TTaa
ttataactctaagctctttaatgacttata---T--C-TT-T------T--G--TG--T-
--T-T--GT-Tgagttca--ACTgttcaggtgtttttgcctactttttgagttttgtatt
tttggatattggaggactctgttttttgcctctctttacttttatcac------------

------------------------------------T-T---A--T-T-Gaa--------
---------------------------G---ATTT-T-------TT---TG---------
-------------------------------------TcaaT---AATAA-AtttCCa-C
Tgagaagcaagttgcc----------------------TTTCT--Tc---G---T-----
-Ctgggttcttcatctcccagtagctcagtagggaagtcattcacctctcatgccagaga
cccgggttctatccccgat------------------------------------C----
CTGACA
>MARY1_LTR
TGTAAG-GgGa-C-----Tagggg--G----------GTG--------------------
--------C---C----C--TG-----TgTGT--T-TTT-Cctattttttatgttcgtcc

catatttttccccacctcatttccttctattcttcattttcta-----------------

-----------------TG---A---TTt-TT--C-CA---GA-TT------TTATta--
------------------CTaA---TTT--Tcc---CTtg-CT-----ATCATGttT-G-
---T-A-T-ATG-TTgA-CAcTtttatatttagaggcggagtaccccttccgacacatac
cccttggtatgatgtcattcggcggccttgatccgcctatctcc----------------

--GCCT------
---------------------------------T--T-TT-C------T--A--TT--T-
--T-TagGT-T---

-------------------------------------aT---A--T-T-T----------
---------------------------G---ATTT-T-------TGtcgTC---------
-------------------------------------T---Tc--A----cAt--ATa-C
Tgggcatcagcaaggggatccttagagt----------TTTCT-------C---T-----
-Tcggatttccctttgctaggtgcgcccccaatccctgtattatgtagcataattttaac
tttggatttccctttgctagaggctcaggttgc----------------------C----

CTTATA

>GYPSY104_LTR_DR

TGTCAT-G-Tc-C-----Tcg-----G----------TTTgatcatctgggccactgggt

g-------T---Tg---T--TGt----TgTTT--T-GTT-Tgtattatctatgtgccttt

ccctagcatgttttgttttgatcctccgccacatgccttctgtttactctctaattccac

gcccatcctcctgtttaagctattacgttattaagt------------------------

-----------------TA---A---TGt-CAc-T-CACcTGT-TC------TTCTtgat

ttctccct----------CT-A---TTTA-Ttc---CTt--CC-----CTTAGCgcT-G-

---TcC-T-GTG-TC-A-GA-Ttgttgtttgcccttctcgtgtttccctcgatctcccgg

tcctgtccagtcagcctttttcctgttgtatctgaatcccggtctacatctttgattttg

cctttttctgctaccaggcccgttcccgtttagtttcattcctgctgctcgcctgtctct

tctctccgcttcagcgatccattttccggcctcctccagccccgcagcgctctctcttcc

tcgcgggtgtcgcggcccaggaaacgcgacccagcagtatcgccccctgccggcggtacc

tattattacaccctctgggagaagcctgtcctctctacgaagaggtttttgttccttgtt

ttttttgttggatttttcggacatttttgttacattttttt-------------------

--CCCT--TTtt

tctgactttag----------------------T--T-TT-C------TaaG--AC--T-

--T-T--TT-T---------CCTctacggagagtttaagtttaag---------------

------------------------------------T-T---T--T-T-Tccctctg---

---------------------------G---ATGT-T-------TT---TGagaattctc

catagtgaag---------------------------C---T---ATTGA-Ac--AT--C

Tta-----------------------------------TTTTC--Ct---C---T-----

-Tcggagctgtttttgttgaagaaccttttcccatctgagtttgctattttgcttatat

ttgaataaactgtgaaggacctgcatttgtgtcttctgtttgccttgatcagatcC----

CTGACA

>GYPSY-115_LTR_DR

TGTCAC-G--g-T-----Ttatg---G----------ATCtgtgtg--------------

--------T---Tc---C--TC-----C-TGTa-T-GTT-Atgtatcatgccttgtgtgt

tgtcatgtgtgagggtgtgtgtttgtttaccttttgtgttgacagcgtgctaggatcagc

tgatgtcatcagctgactta--

-----------------TC---A---T-c-CT--C-CAGcTGAACC------TCATttgg

ctgg--------------CT-A---TATA-A-----TT---C------CTCCTG--T-Gg

---T-A-T-GTG-CT-G-GT-Tatcagtccgt----------------------------

--TGTT--GTcc

---------------------------------T--T-GT-T------T--G--TC--C-

--T-T--GT-T---------CCTgactgttgtctcccagtatcctccgctgtctgtttga

tgtcwtcgttgtggcctgtggctctctt--------------------------------

------------------------------------T-Tg--T--T-T-T----------
---------------------------Ga--ACTT-Tcg-----TG---TGtcctctg--
-------------------------------------T---Tc--ATTAA-Aa--CA--C
Tcgcatttggatcctc----------------------CTTGT--Ctaa-Ga--T-----
-T--
---C----
GTGACA
>GYPSY-170_LTR_DR
TGTCAT-GgT--C-----T-------G-----------TCacctgca-------------
--------Tg--T----Tg-TCacagtCaTTCg-T-GTT-Tgttttctcgtagcacatgg
cattgttttgacagcgcgccatgtgctccactgcctcactccacccacttatcaggttac
tcattagatcattatt--

-----------------TC---A---TTa-TT--CgCACcTGTCTC------TAGTctta
gccttatgagtctcc---CT-A---TTTA-T------T---CT-----CCTCTC--T-G-
---T-C-A-TTG-TCt-tGTaCcgggtcgttagtgtgtttacctcacgcggtggcctctt
agatcgtgtcctgtcttgtatccatttttgactccagtgccagttttttttgatttc---

--GATT--TTgt
ggtttggac------------------------Tg-T-TT-Cc-----T--G--TT--T-
--T-T--TC-C---------CCTtttatcagatcgccctcgctgctatcagttgtggcag

acgcttccctctccacttcctgcttcagccttttctatcccgcccaccagcgatcttctc

cctctctggccgccttgtatcccgcccgggcgctttcacttcggcggctgatactgctgt

gtttcccgtttgactggagagttggaccaccgtgcgcctcctgctggaagcactttatat

tgcccttcacctgagttacccatttcctgttttttgactgagagttggactgccgtgcgc

ctcctgctggaagcacttagtattgcctgtaattataccttccgtttttgcccctttttg

actggcgactttggatcgctgagcgccctctgttgaactttctttatttattttttgcca

gtattttgtgctttttctgtggcatttacagcacccT-G---T--GcT-G----------

---------------------------Aga-ATTT-T-------TTgt-TGtataatatc

ccctctgcaggccact---------------------T---TagtAATAA-Ata-ATt-C

Tg------------------------------------TTTTT--Cc---C---Tg----

-Catttgattcctcctgattttttatactgacacttg-----------------------

---T----

ATGACA

>GYPSY145_LTR_DR

TGTTTT-G-Cc-C-----C-------Gcataca----ATT--------------------

--------C---Taag-Cg-TT-----T-TTCtcT-GTT-Agtgatctgattctgtgttt

tgaccccagactgtttttcccgtttcct--------------------------------

-----------------TG---A---TTg-TTgaTgCCGcTGCCTT------TTGTgacc

ctctg-------------CT--cagTTTA-A-----CG---GAac---ATTCTCtcT-Gc

---T-G-T-ATG-CCg-cCTgTctcgaccaatcgcctgcccacggactctgagttaagtt

tctccttcgcca--

--TCCTtgTT--
---------------------------------Tg-T-TG-T------T--G--TC--T-
--G-A--TC-Ctg-------CCTgtacgaccatcc-------------------------

------------------------------------Tg----------------------
---------------------------------TT-G-------TGat-TCaacaaagct
g------------------------------------C---A---AATGG-A---TC--C
Tcaa----------------------------------TCTCC--CgacgC---T-----
-C--
---C----
TTTACA
>GYPSY164_LTR_DR
TGTCACcG-TacC-----Ttgt----G---------TTC--------------------
--------T---Ca---C--TC-----TcTTC--A-GTT-Ctatgttacagattaccttc
agtcacctgcttttctagtattcacaggtcatcggag-----------------------

-----------------TG---A---TTtgCT--C-CACcTGTTTC------TCATccct
ttgtta------------CT-A---TTTA-Tag---CTtgtCA-----TTTCCCt-T-Ca
---T-G-T-TTG-TC-A-GT-C--------------------------------------

--TGTT--GT--
---------------------------------Tg-T-TG-G------T--G--TG--T-
--G-T--GTaTaacgg----TCTgttactcaccccatatttgtgtccacggctgccagtg
aacgagtctcccgttgtttctggaatgttttccacggcactcccctggcactcacccgat
tgggtt--

------------------------------------T-T---T--TgT-T----------
---------------------------T---ATTT-GcaaagacTT---TCtcc------
-------------------------------------T---T---AATAA-A---CA--C
Tttgtgttctgcacttgagttcactc------------CTTCT--T----C---Tac---
-C--
---C----
GTGACA
>GYPSY159_LTR_DR
TGTTGT-G-Aa--------------------------ATC--------------------
--------Cac-T----Ca-TGaa---CgTTCc-TcGTG-Tgaccaccagagggagccat
cgcctgagttctaatctccccaaggctcgactgggac-----------------------

-----------------TG---A---TTa-CA--TaCAGcTGAAAC------CCATttca

gactct------------CT-A---TATA-Aa----CG---CA-----CACATCctT-Ca

c--T-C-G-GTG-TGaA-GT-Tttgattagccccggccatcatctctgagcgtta-----

--CCTT--GT--

---------------------------------Tg-T-TTaT------T--G---C--C-

--T-A--TC-TgtgtatcgaCCTggactgtcttaccgtttctgaagctatctgcctgcct

tgaccactgcatagttatattgattccttctgccagccgcctgcctagaatctctgcctg

tctctcgttaacgatcactctctcctagccccgacccacgcctgctcttcgactattcta

ctggaaagttcaa---

------------------------------------T-T---Tc-TaTcT----------

---------------------------G---ATGT-T-------TG---TGagtttccgg

ctgtgatatcttactg---------------------T---T---ATTAA-A--------

---------------------------------------GTGTg----------------

----CA

>GYPSY69_LTR_DR

TGTAAC-A-Aa-CagaacT-------GacaaccactcATCcaacctgcagagggagccct

caccggaaT---T----C--TGa----C-TGTc-A-GTT-Cttcgttggttacttcctgt

ttggtggccatattaacctggccacaccaaacactcaa----------------------

-----------------TGcgaA---GTa-TTg-C-CA---GTATC------TGCTgcct
taccaagc----------GT-A---TTT--Tcc---CT---GT-----TTGATC--T-G-
---C-CtT-TTG-TT-AtGA--

---CCT--TTga
gcctgttctttttgactactgattttggaagacTg-T-TT-T------T--G---C--C-
--T-T--GT-Ttg-------CCTtggtggacactctgtttgttactttgaacctgcctgc
tttttgactacgatttagcctag-------------------------------------

------------------------------------T-G---T--T-T-T----------
---------------------------Gg--ATTT-G-------TT---TActattta--
-------------------------------------C---T---ATTAA-Ac--TT--C
Ttgcatttggatcccatc--------------------TGTTT--C----CatgT-----
-Cgtcaccaccgccat--
---T----
GTCACA

```
>GYPSY138_LTR_DR
TGTAAC-G--g-T-----Ta------G----------TAC--------------------
--------TcgaT----T--TCc----TcTGCatT-GTG-Tgtagtagagatg-------
------------------------------------------------------------
------------------------------------------------------------
------------------------------------------------------------
------------------------------------------------------------
------------------------------------------------------------
-----------------AG---A---TT------------TGACAC------CCA-----
------------------------------A------T---CA-----CGCCTCa-Aa--
--------------C-A-GA-T--------------------------------------
------------------------------------------------------------
------------------------------------------------------------
------------------------------------------------------------
------------------------------------------------------------
------------------------------------------------------------
------------------------------------------------------------
--------------------------------------------------CACT--TT--
-------------------------------------aTT-C------T--G--------
------------------------------------------------------------
------------------------------------------------------------
------------------------------------------------------------
------------------------------------------------------------
------------------------------------------------------------
------------------------------------------------------------
------------------------------------------------------------
------------------------------------------------------------
--------------------------------------------------Gcaaa-----
-------------------------------------C---Ac--AATGA-Aa--ACa-C
```

Agtgatcagtcccagac---------------------AGTCC-----------------

--AACA
>GYPSY64_LTR_DR
TGTCAG-G-Tc-C-----Tctgtct-G----------ATCac------------------
--------C---Tggg-T--TC------gTTTg-T-GTT-Ttgcacttgtttacatgtat
ttcagcacgtgtggtgttttgatcgctctctcattacatacttgctttgacacatctcct
cccatcttcccactcatccctattattcaattaaac------------------------

-----------------CA---A---TTt-CAc-T-CACcTGTCTTc-----CCGTaatt
tcccct------------CT-A---TTTA-Aag---CG---CT-----CTCATGt-T-G-
---T-CaT-TTG-TC-A-GA-Tcgttgtcgtttggttgtggtcagcgtgcactgtttcac
tagtccagtccagtcagttcctgttgtctcgagcccgtcttctggttggtttt-------

---TCCT--TTta
ttt------------------------------Ta-T-TT-C------T--T--CA--T-
--T-T--TT-Tgacc-----CCTctctgtcatctgcaggacctgtttctgttttgtttat
ccgccgtggttggccgattgtctccgatccccggcttcaatattccctcccgtcacctgc
tccactcttcttctccctctcggctctgccgcccgccggatccagtataccgctgtatcg
ccccctgccgagtgaacttgaaattacaccctgaaagacattttgtcccttctacggagc
ggtttctgttgacagtttttttagctctttcttctgaacaaccttgaagtttgttttagt
ttcattttgttaatttatttgcctgagtccctttaaagatcccagtaataattatatcc

tcttcggagcagtttttgttgaactttttatttttataaa--------------------

------------------------------------T-T---T--T-T-Gattagaattt

ttctccctgtgcttaagcccaatagggTc--ATTT-T-------TG---TCa--------

-------------------------------------C---T---AATAA-A---TT--C

Tgaaagagacctgcact---------------------TGTGT--Cc---G---Ttctgt

tT--

---C----

CTGACA

>hmmcons

TGTCAC-G-T--C-----T-------G----------TTC--------------------

--------T---T----C--TC-----T-TTT--T-GTT-T-------------------

-----------------TG---A---TT--CT--T-CAC-TGTCTC------TCAT----

------------------CT-A---TTTA-T-----CT---CT-----CTCCTG--T-G-

---T-C-T-TTG-TC-A-GT-T--------------------------------------

--GCCT--TT--

---------------------------------T--T-TT-T------T--G--TT--T-

--T-T--TT-T---------CCT-------------------------------------

------------------------------------T-T---T--T-T-T----------

---------------------------T---ATTT-T-------TG---TG---------

-------------------------------------T---T---AATAA-A---CT--C

T-------------------------------------TTTCT--C----C---T-----

-C--

---C----

CTGACA

………………………

Zam_150

>GYPSY43_LTR_AG

AGTAATAG-------------ATAgTGtGtttattattatatgttt--------------

----------------------------------ACAT--ataaaacgttca--------

------------------------------------AtCA-CACCTCACc----ATc---

-------A--------tA---------TAAAAAC-C-TTATtcAACT-Gacgt-------

-GTTGCGctgT-------------C--AGTTT-G-AtGA--------TaA-Ac--GCAGG

------Ctcc-------TCA--aT----------AAag--TC------------------

-------------------------------------ATTa-TTATTccg--AT---CGT

TA----AAg--------------AGAa---AGgacacaaac--A-CAAc------Accc-

------------------ACGA-------CTtg---------------------------

----------------------T-A-A-T-T--

>TED_LTR

TGTTAGGT-Atggagcctta---A-TG-G-------------------------------

----------------------------------ATAT--catcgacgctgcatttcctg

ttattgtccgccagctgcatagaaactgtctgaatgacgtaa------------------

------------------------------------AtC-----GTCATg----AAccgc

tg-----AtGtAGC-G-Aaatt-----TGTAATT-AgTTATt-AACT-Caaaattg----

-TATGCA---Ttcc-----------t-ATTTC-T-AaTA--------TcG-A---GTAGG

tctcac-----------GCA--tT----------AA----TT------------------

-------------------------------------ATTg-TAATTctt--AT---AAG

TA-AtAAAtt-------------AGCaTtTAAa----------A-TCA-------Ttttt

ggtTT-TTTTTctatCTGCCGT-------CTgcagtatacg-------------------

----------------------T-A-A-T-T--

>GYPSY41_LTR_AG

AGTTACGT-Agaccg-----AATA-----gctgagtccacgtaataagaagccaacacgt

gg--------------------------------ACACGCgcaccgagcaacgaccgcat

caccggaacacgcgcgcat---

------------------------------------AgCAaCATGACGCgc---AT----

--------aGcAACgGgAaccggcacgTGGACACgC-GCAT--ACCG-Ga----------

-TATGCA----gagtcag-------c-AGCTA-T-AgGG--------TaG-AatcGAAGG

------CtaggtgcattGTAGAaTttaagaaataAG----TT------------------

-------------------------------------AGTtgTAATTtgg--ATc--AGC

TC----AGtgtaagaagcgcttgCGC-TtAAGca---------A-TAA-------Agt--

---TT-TTTTTt---ATGAAAC-------GTgaagccttgcactta--------------

----------------------T-A-A-T-T--

>GYPSY40_LTR_AG

AGTTATAT-Gcgtgg-----AACA-----caa----------------------------

----------------------------------ATAC--aggtgcgacatcactgcaac

agcctcgcacc---

--------------------------------------CAtGCCGATACg----AT----

--------aGgAGCcG-A---------T-----T-C---------CG-T-----------

---CGCG---T-------------CagATCGG-T-A-TC--------AgC-At--CCAAG

------Cgataagcac-GCA--aT----------AA----TC------------------

-------------------------------------ATT------------TT---AAA

TA----AA--------------AGC-TgTGG-----------------------------

---TTc------------CATA-------CTca---------------------------

----------------------T-A-A-C-T--

>GYPSY39_LTR_AG

AGTTATGT-A----------CACA-TGcG-------------------------------

----------------------------------CTACGCgctatgatacaggtgctgag

taagaaaacggtcgcaagcgagccatcgatgttactccacgcgcggagtcaagttccaac

gggaactccactggaagcaggttcccacg-------------------------------

------------------------------------AcCA-GGTGTGGTgtcgtATgttc

ag-----AcGgACC-G-Aggcaaca--TGATCAT-C---ATa-AACG-Tg----------

-GACGAC----aacgtgaccggca-a-ACCTGg-c-aGC--------Ac-c----GAAAA

------Ca---------CCAGCcT----------AG------ctaaatagtaccgaatcc

agaagttagcttt------------------------AGTc-TTAGT-----TTagcAGT

TC-GcAAA-------------------TaAAGatcccc-----AgTAA-------Tgtt-

---TT-TTTTT-------AAAA-------CTtactccgggctatcgtaaaca--------

----------------------T-A-A-T-T--

>GYPSY42_LTR_AG

AATTATGT---------------------ggtataaccattattaaccttggtactggta

taacggtg--------------------------ATACGCgtggctgtagtggcagcttt

gt--

--------------------------------------GAcGGTGACATtcg--ATcg--
-------A-G-AGAcG-A---------T-------C-TGAT--AACT-Ctc---------
-TTT-------ttccgt--------t-ATCTCc-c--GAgcagtaca--G-AcgtCTAGG
------A----------CGAGA-T----------AGgtttTT------------------
-------------------------------------TTTc-TTATTtttttAT---AGT
TA--gGAGtt-------------AGGaTtTAGgttatgttagaA-TAAgttagttTa---
---TT-TTTTTgtaaATAAAAA-------CT-----------------------------

----------------------T-A-A-T-T--
>PIFO_LTR
AGTTAC--cAcagtcac---CACA-----ccccctaaaccc-------------------
----------------------------------CCACGCctacaccactgaacacatcg
acgcccatgggcactccggtacaacgaaccgggaaacgaataaacaaatgggtcaacaat
gtatctacaatgtatcgacatccggccaaaatgctgacactaacatcagcagaccagacg
gcaagaatgccgatgcagcataagaacctagcata-AcC-----CTTATat---AT----
-------AaG-AAC--tA---------TGTACTT-A-----------g-a----------
-TTTGTA----ggc-----------t--TCTT-G-A-GA--------AgA-Ac--GAAAG
------Aaat------------aT----------AGaa--TC------------------
-------------------------------------ACTctTAAGT-----TTg--AAC
CC----AA---------------AGCgTgAAG----------------------------
---TT-GTGTTa---CTGAATC-------CCatacaagtgcctgaacaaaatctcatgca
aagtgccgacagccgactataagcgaaccacccaacatctttactgctgttatattcacc
tcccatcattcggcccgaatggT-A-A-C-T--
>GYPSY8_LTR_DYA
TG--ACGG------------CACA-----agcatgccttcacctcccccgaaacccccac
gggcagcgacctacatatgagacactcaacccggACACACaccgcagcaaagtcatcaac
ccacactggatcagcagagt--

--------------------------------------CAg---GATTCcc---AAgaat

ccaaccgAaGcACTgGaA---------GGGGCAT-C---------CG-Ggtgaaaagtgc

cGACGCA----agg-----------g-GTTTCg-c-aGA--------AcA-Aaa-GAA--

------Ttgtacg----CTAGGcT----------AA------gaggaaaatttattcaaa

aaataaaatcattctgtcaccgaacttcgaacgagtcACTt-TAA-------AT---ACT

TA----AAatcctcctt------GGCtTaAAG-----------A-TAA-------Aa---

------------------CAGAacagatcCT-----------------------------

-----------------------c-g-a-c-ca

>hmmcons

AGTTATGT-A----------CACA-TG-G-------------------------------

----------------------------------ACACGC--------------------

-----------------------------------TA-CA-GGTGACAC-----AT----

-------A-G-AGC-G-A---------TGAACAT-C-TTAT--AACG-G-----------

-TATGCA---T-------------C--ATCTC-T-A-GA--------A-A-A---GAAGG

------A----------CCAGA-T----------AA----TT------------------

-------------------------------------ATT--TAATT-----TT---AGT

TAAA-AAA---------------AGC-T-AAG-----------A-TAA-------T----

---TT-TTTTT----ATGAAAA-------CT-----------------------------

----------------------T-A-A-T-T--
